# Ethnic and socioeconomic differences in SARS-CoV-2 infection: prospective cohort study using UK Biobank

**DOI:** 10.1186/s12916-020-01640-8

**Published:** 2020-05-29

**Authors:** Claire L. Niedzwiedz, Catherine A. O’Donnell, Bhautesh Dinesh Jani, Evangelia Demou, Frederick K. Ho, Carlos Celis-Morales, Barbara I. Nicholl, Frances S. Mair, Paul Welsh, Naveed Sattar, Jill P. Pell, S. Vittal Katikireddi

**Affiliations:** 1 Institute of Health & Wellbeing, College of Medical, Veterinary and Life Sciences, University of Glasgow, Glasgow, G12 8RZ UK; 2 General Practice and Primary Care, Institute of Health and Wellbeing, College of Medical, Veterinary and Life Sciences, University of Glasgow, 1 Horselethill Road, Glasgow, G12 9LX UK; 3 MRC/CSO Social & Public Health Sciences Unit, University of Glasgow, Top floor, 200 Renfield Street, Glasgow, G2 3QB UK; 4 BHF Glasgow Cardiovascular Research Centre, Institute of Cardiovascular and Medical Sciences, College of Medical, Veterinary and Life Sciences, University of Glasgow, Glasgow, G12 8TA UK; 5 Public Health Scotland, Meridian Court, 5 Cadogan Street, Glasgow, G2 6QE UK

**Keywords:** Ethnicity, Inequality, Coronavirus, COVID-19, SARS-CoV-2, Health inequality, Infectious disease, Social factors, Pandemic

## Abstract

**Background:**

Understanding of the role of ethnicity and socioeconomic position in the risk of developing SARS-CoV-2 infection is limited. We investigated this in the UK Biobank study.

**Methods:**

The UK Biobank study recruited 40–70-year-olds in 2006–2010 from the general population, collecting information about self-defined ethnicity and socioeconomic variables (including area-level socioeconomic deprivation and educational attainment). SARS-CoV-2 test results from Public Health England were linked to baseline UK Biobank data. Poisson regression with robust standard errors was used to assess risk ratios (RRs) between the exposures and dichotomous variables for being tested, having a positive test and testing positive in hospital. We also investigated whether ethnicity and socioeconomic position were associated with having a positive test amongst those tested. We adjusted for covariates including age, sex, social variables (including healthcare work and household size), behavioural risk factors and baseline health.

**Results:**

Amongst 392,116 participants in England, 2658 had been tested for SARS-CoV-2 and 948 tested positive (726 in hospital) between 16 March and 3 May 2020. Black and south Asian groups were more likely to test positive (RR 3.35 (95% CI 2.48–4.53) and RR 2.42 (95% CI 1.75–3.36) respectively), with Pakistani ethnicity at highest risk within the south Asian group (RR 3.24 (95% CI 1.73–6.07)). These ethnic groups were more likely to be hospital cases compared to the white British. Adjustment for baseline health and behavioural risk factors led to little change, with only modest attenuation when accounting for socioeconomic variables. Socioeconomic deprivation and having no qualifications were consistently associated with a higher risk of confirmed infection (RR 2.19 for most deprived quartile vs least (95% CI 1.80–2.66) and RR 2.00 for no qualifications vs degree (95% CI 1.66–2.42)).

**Conclusions:**

Some minority ethnic groups have a higher risk of confirmed SARS-CoV-2 infection in the UK Biobank study, which was not accounted for by differences in socioeconomic conditions, baseline self-reported health or behavioural risk factors. An urgent response to addressing these elevated risks is required.

## Background

The severe acute respiratory syndrome coronavirus-2 (SARS-CoV-2) and its resulting disease (COVID-19) are spreading rapidly worldwide [1]. A better understanding of the predictors of developing infection is essential for health service planning (e.g. ensuring adequate facilities for those most at risk), targeting prevention efforts (e.g. targeted shielding or surveillance) and informing future modelling efforts. Age, male sex and pre-existing medical conditions are established predictors of adverse COVID-19 outcomes, as is excess adiposity [2], but the role of social determinants is poorly understood [3, 4].

Ethnicity and socioeconomic position strongly influence health outcomes for both infectious and non-communicable diseases. Previous pandemics have often disproportionately impacted ethnic minorities and socioeconomically disadvantaged populations [5, 6]. Early evidence suggests that the same may be occurring in the current SARS-CoV-2 pandemic but empirical research remains highly limited [7]. It is highly plausible that infection risk will vary across these social groups. For example, socioeconomic disadvantage is linked to living in overcrowded housing. Similarly, Bangladeshi, Indian and Chinese households are more likely to live in intergenerational households (e.g. with children, parents and grandparents) [8], which has been hypothesised to increase transmission [9].

Establishing the risk of developing infection across different social groups is challenging. A major issue is that information about ethnicity and socioeconomic position are often not well collected within routine health data. Furthermore, the size of the different social groups in the general population is also often not accurately known [10]. The ideal approach to estimating infection risk across different social groups is to analyse data from a cohort study, but most existing cohort studies which include detailed information about ethnicity and socioeconomic position are subject to long delays in data being available for analysis and are too small to provide useful estimates of infection risk.

The UK Biobank study has carried out data linkage between its study participants and SARS-CoV-2 test results held by Public Health England. We therefore aimed to investigate the relationship between ethnicity, socioeconomic position and the risk of having confirmed SARS-CoV-2 infection in the population-based UK Biobank study.

## Methods

### Study design and participants

Data were obtained from UK Biobank (https://www.ukbiobank.ac.uk/), with the methods described in detail previously [11]. In brief, over 502,000 community-dwelling individuals largely aged 40 to 70 years were recruited to the study during 2006 to 2010. Participants attended one of 22 assessment centres across England, Scotland and Wales. Data were collected on a range of topics including social and demographic factors, health and behavioural risk factors, using standardised questionnaires administered by trained interviewers and self-completion by computer.

Results of SARS-CoV-2 tests for UK Biobank participants, including confirmed cases, were provided by the Public Health England (PHE) microbiology database Second Generation Surveillance System and linked to UK Biobank baseline data [12]. Data provided by PHE included the specimen date, specimen type (e.g. upper respiratory tract), laboratory, origin (whether there was evidence from microbiological record that the participant was an inpatient or not) and result (positive or negative). Data were available for the period 16 March 2020 to 3 May 2020.

Since data on test results were only available for England, we restricted the study population to people who attended UK Biobank baseline assessment centres in England. Participants who were identified as having died prior to 31 January 2018 from the linked mortality records provided by the NHS Information Centre (*N* = 17,632) and those who requested to withdraw from the study prior to February 2020 (*N* = 30) were also excluded from the analysis. In addition to the analyses of the overall population, we also investigated positive test results amongst those who had been tested only. This allowed us to investigate the potential for bias due to differential testing between ethnic and socioeconomic groups. UK Biobank received ethical approval from the NHS National Research Ethics Service North West (11/NW/0382; 16/NW/0274).

### Assessment of ethnicity and socioeconomic position

All exposures were derived from the baseline assessment centre data collection. Ethnicity was self-reported and categorised into white British, white Irish, other white background, south Asian, black (Caribbean or African), Chinese, mixed or others. As more data became available, we also used more refined groupings, separating south Asian into Indian, Pakistani or other south Asians (including Bangladeshi) and black into Carribean, African or other black. Due to small numbers, analyses of the Chinese, mixed and other black groups were limited. In line with previous research, we also do not report results for the other group due to problems with interpretation of this highly heterogenous group [13].

Socioeconomic position was assessed using two different measures recorded at the baseline visit. Area-level socioeconomic deprivation was assessed by the Townsend index (including measures of unemployment, non-car ownership, non-home ownership and household overcrowding), corresponding to the output area in which the respondent’s home postcode was recorded [14]. Quartiles were derived from the index, where the lowest quartile represents the most advantaged and the highest the least advantaged. Highest education level is a proxy measure for socioeconomic position and usually remains stable throughout the adult life course. It was assessed as (1) university or college degree; (2) A levels or equivalent; (3) O levels, General Certificate of Secondary Education (GCSE), vocational Certificate of Secondary Education (CSE) or equivalent; (4) others (e.g. National Vocational Qualifications or other professional qualifications); or (5) none of the above [15].

### Ascertainment of SARS-CoV-2 outcomes

We defined our primary outcome as having a positive test within the Public Health England database available through linkage [12]. This reflects confirmed infection but does not include symptomatic individuals who have not presented to the health service or not been tested, or asymptomatic cases. Some systemic differences exist in testing threshold. For example, healthcare workers may be more likely to be tested and therefore observed differences may reflect differences in testing practices. To investigate whether differential ascertainment was biasing our results, we studied three further outcomes. We identified positive cases that had their test taken while attending hospital (i.e. either emergency departments or as inpatients—hereafter referred to as hospital cases). This group is likely to reflect more severe illness and therefore is less likely to be subject to ascertainment bias. In addition, we investigated outcomes related to testing practice by assessing the risk of being tested in the overall population and testing positive amongst only those who had been tested. Higher levels of confirmed SARS-CoV-2 infection could arise from higher rates of testing amongst some population subgroups [12]. However, if this were to occur, the likelihood of having a positive test would be lower amongst groups experiencing high rates of testing.

### Potential confounders and mediators

Age group (5-year age bands), sex and assessment centre were included as potential confounding variables in all statistical models. Country of birth (UK and Ireland) versus elsewhere was also included, given its influence on cultural practices [16]. We also included several variables which could reflect potential confounding or mediation.

Baseline health status was assessed using self-reported longstanding illness, disability or infirmity (yes or no), self-reported health status (excellent, good, fair, poor) and the number of chronic health conditions self-reported from a pre-defined list of 43 conditions and top-coded at 4 or more, based on a previously published approach [17]. Behavioural factors included smoking (never, previous, current), body mass index (BMI) (weight/height^2^ derived from physical measurements and classified into underweight, normal weight, overweight, obese) and alcohol consumption (categorised into daily or almost daily, 3–4 times a week, once or twice a week, 1–3 times per month, special occasions, former drinker or never).

Other social variables were also considered. Employment status distinguished those in paid employment or self-employment, retired, looking after home and/or family, unable to work because of sickness or disability, unemployment or others. For those in work, manual versus non-manual occupation was assessed by asking participants to report whether their job involved heavy manual or physical work (never/rarely/sometimes versus usually/always). Participants were asked about the title of their current or most recent job at baseline and these were converted to the Standard Occupational Classification (SOC 2000 [18]) by UK Biobank. Healthcare (and related) workers were identified from the SOC 2000 codes 22 (Health Professionals), 32 (Health and Social Welfare Associate Professionals), 118 (Health and Social Services Managers), 611 (Healthcare and Related Personal Services), 9221 (Hospital porters) and 4211 (Medical Secretaries). Housing tenure was categorised into owner-occupier or renter/other (including those who lived in accommodation rent free, in a care home or sheltered accommodation). Urban/rural status was derived from data on the home area population density; UK Biobank combined each participant’s home postcode with data generated from the 2001 census from the Office of National Statistics. The number of people within a household was categorised into four groups: single person, two people, three people or four or more people (which included those living in institutions, such as care homes).

### Statistical analyses

The association between the exposures (ethnicity and socioeconomic position) and the outcomes of interest (confirmed infection, hospital case, being tested and having a positive test amongst those tested) was explored using Poisson regression. Poisson regression was preferred over logistic regression to allow relative risks to be presented, rather than odds ratios which are often misinterpreted [19]. Robust standard errors were used to ensure accurate estimation of 95% confidence intervals and *p* values. Missing data were excluded from the analysis via listwise deletion. Statistical analysis was conducted using Stata/MP 15.1.

To investigate ethnic differences, we initially adjusted for age, sex and assessment centre (model 1) and then added country of birth (model 2). Subsequent models additionally adjusted for variables which we hypothesised were likely to be at least partially mediating rather than confounding variables. Model 3 adjusted for model 2 variables and for being a healthcare worker. Model 4 additionally adjusted for social variables (namely urbanicity, number of people per household, highest education level, socioeconomic deprivation, tenure status, employment status, manual work); model 5 was adjusted for model 2 plus health status variables (self-rated health, number of chronic conditions and longstanding illness or disability); model 6 was adjusted for model 2 plus behavioural risk factors (smoking, alcohol consumption and BMI); and model 7 was adjusted for all aforementioned covariates. In post hoc analyses, we also repeated the above with the more defined ethnic groups.

We followed a similar approach to explore the role of socioeconomic deprivation and education level. Model 1 was adjusted for age, sex and assessment centre; model 2 added ethnicity and country of birth; model 3 also adjusted for the social variables (as above); model 4 adjusted for model 2 plus health status variables; model 5 was adjusted for model 2 plus behavioural risk factors; and model 6 was adjusted for all previous covariates.

## Results

A total of 392,116 participants were included in the study (after excluding 36,109 (8.4%) people with missing data, Additional file Figure S1 for flowchart and Table S1 for patterns of missing data by ethnicity and socioeconomic position). Most of the baseline UK Biobank sample in England was white British, with the next largest groups being other white, white Irish and then south Asian and black (Table 1 and Additional file Table S2). Approximately one-third (32.9%) of the sample had a degree and 16.2% had no formal qualifications. In our sample, 2658 people had been tested for SARS-CoV-2 and 948 had at least one positive test (726 received a positive test in a hospital setting suggesting more severe illness) (see Additional file Table S3 for outcomes by ethnicity, socioeconomic deprivation and education level). The geometric mean number of tests performed per participant tested was 1.53 (95% CI 1.50–1.56).

**Table 1:** Description of the study population

	Number	Percentage
**Tested for SARS-CoV-2**		
No	389,458	99.3
Yes	2658	0.7
**Tested positive for SARS-CoV-2**		
No	391,168	99.8
Yes	948	0.2
**Tested positive for SARS-CoV-2 in hospital**		
No	391,390	99.8
Yes	726	0.2
**Age group at baseline**		
40–44	40,995	10.5
45–49	52,116	13.3
50–54	60,291	15.4
55–59	71,160	18.1
60–64	95,604	24.4
65–69	70,110	17.9
70+	1840	0.5
**Sex**		
Female	215,351	54.9
Male	176,765	45.1
**Ethnicity**		
White British	348,735	88.9
White Irish	9800	2.5
White Other	12,925	3.3
Mixed	2356	0.6
Indian	4571	1.2
Pakistani	1259	0.3
Other South Asian	1493	0.4
Black Caribbean	3669	0.9
Black African	2623	0.7
Black Other	103	0.0
Chinese	1153	0.3
Others	3429	0.9
**Country of birth**		
UK and Ireland	361,025	92.1
Elsewhere	31,091	7.9
**Number in household**		
1	69,862	17.8
2	183,777	46.9
3	62,934	16.0
4+	75,543	19.3
**Education level**		
College or university degree	128,890	32.9
A levels/AS levels	44,650	11.4
O levels/GCSEs/CSEs	108,648	27.7
Others	46,393	11.8
None of the above	63,535	16.2
**Deprivation quartile**		
Quartile 1 (most advantaged)	100,701	25.7
Quartile 2	99,838	25.5
Quartile 3	98,380	25.1
Quartile 4 (least advantaged)	93,197	23.8
**Housing tenure**		
Own	352,079	89.8
Rent/others	40,037	10.2
**Urban/rural**		
Urban	334,570	85.3
Rural	57,546	14.7
**Employment status**		
In paid employment or self-employed	230,190	58.7
Retired	128,613	32.8
Looking after home and/or family	10,956	2.8
Unable to work because of sickness or disability	11,111	2.8
Unemployed	6386	1.6
Others	4860	1.2
**Manual occupation**		
Non-manual	199,564	50.9
Manual	30,626	7.8
Not in employment	161,926	41.3
**Healthcare worker**		
No	204,254	52.1
Yes	25,936	6.6
Not in employment	161,926	41.3
**Long-standing illness, disability or infirmity**		
No	268,919	68.6
Yes	123,197	31.4
**Number of chronic conditions**		
0	147,943	37.7
1	130,034	33.2
2	69,222	17.7
3	28,957	7.4
4+	15,960	4.1
**Overall health rating**		
Excellent	65,560	16.7
Good	231,672	59.1
Fair	79,347	20.2
Poor	15,537	4.0
**BMI category**		
Underweight (< 18.5)	1928	0.5
Normal weight (18.5–24.9)	129,755	33.1
Overweight (25.0–29.9)	166,979	42.6
Obese (≥ 30.0)	93,454	23.8
**Smoking status**		
Never	217,297	55.4
Previous	136,482	34.8
Current	38,337	9.8
**Alcohol consumption**		
Daily or almost daily	81,567	20.8
Three or four times a week	92,308	23.5
Once or twice a week	100,956	25.7
One to three times a month	43,743	11.2
Special occasions only	43,916	11.2
Never (former drinker)	13,315	3.4
Never	16,311	4.2
**Total**	392,116	100.0

**Fig. 1:**
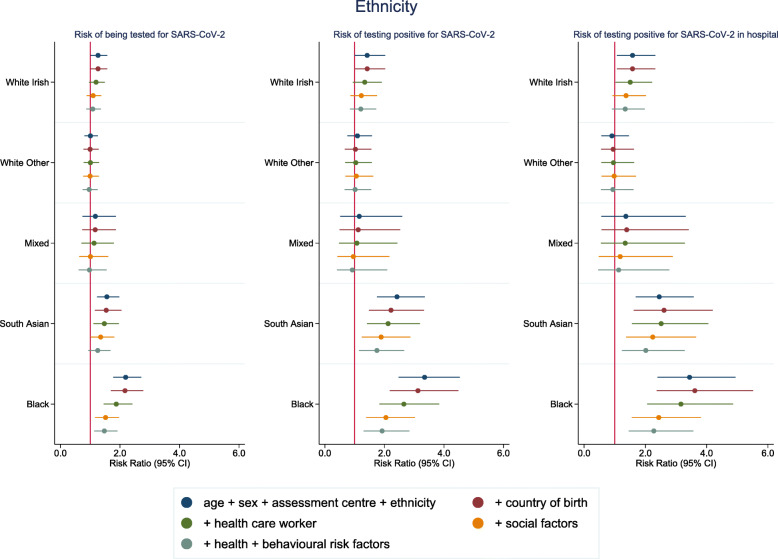
Risk ratios for associations between broad ethnicity groups (white British as the reference category) and SARS-CoV-2. Model 1: age, sex and assessment centre. Model 2: model 1 + country of birth. Model 3: model 2 + healthcare worker. Model 4: model 3 + social variables (urbanicity, number of people per household, highest education level, deprivation, tenure status, employment status, manual work). Model 5: model 4 + health status variables (self-rated health, number of chronic conditions and longstanding illness) + behavioural risk factors (smoking, alcohol consumption and BMI). Coefficients for the Chinese and other groups are not shown

In comparison to the white British majority ethnic group, several ethnic minority groups had a higher risk of testing positive for SARS-CoV-2 infection and also testing positive while attending hospital (Fig. 1 and Additional file: Tables S4 and S5). Black participants had the highest risk (RR 3.35 (95% CI 2.48–4.53)), with adjustment for the country of birth resulting in little attenuation (RR 3.13 (95% CI 2.18–4.48)); adjustment for a history of being a healthcare worker (RR 2.66 (95% CI 1.83–3.84)) and for social factors (including measures of socioeconomic position) did additionally attenuate the risk (RR 2.05 (95% CI 1.39–3.03)). South Asians also had an elevated risk of testing positive (RR 2.42 (95% CI 1.75–3.36) in model 1), with a broadly similar pattern of attenuation as for the black ethnic group. The white Irish group also had a marginally elevated risk of having a positive test (RR 1.42 (95% CI 1.00–2.03)) which attenuated with adjustment for social variables (RR 1.23 (95% CI 0.86–1.75). The Chinese group had imprecisely estimated risk ratios due to smaller numbers. The pattern of findings for hospital cases was similar (Additional file S5), suggesting that the higher testing rates amongst certain ethnic groups in the community were not skewing the results. Similarly, analyses of the likelihood of testing positive amongst those who had been tested were often higher or the same in these ethnic groups (Table 2 and Additional file S16), whereas a lower risk would have suggested differentially high testing.

**Table 2:** Risk ratios for testing positive for SARS-CoV-2 amongst those tested (*N* = 2658) in UK Biobank

	Model 1	Model 2	Model 3	Model 4	Model 5	Model 6
	RR [95% CI]	RR [95% CI]	RR [95% CI]	RR [95% CI]	RR [95% CI]	RR [95% CI]
**Ethnicity**						
White British (reference group)	1.000	1.000	1.000	1.000	1.000	1.000
White Irish	1.166 [0.877, 1.549]	1.165 [0.877, 1.548]	1.133 [0.853, 1.505]	1.169 [0.877, 1.556]	1.150 [0.866, 1.527]	1.132 [0.851, 1.507]
White Other	1.093 [0.814, 1.467]	1.034 [0.737, 1.452]	1.020 [0.718, 1.448]	1.045 [0.743, 1.470]	1.037 [0.739, 1.454]	1.020 [0.718, 1.449]
South Asian	1.490*** [1.189, 1.868]	1.384* [1.011, 1.894]	1.270 [0.917, 1.759]	1.382* [1.009, 1.892]	1.355 [0.974, 1.885]	1.279 [0.908, 1.802]
Black	1.489*** [1.215, 1.825]	1.405* [1.075, 1.836]	1.324* [1.011, 1.734]	1.388* [1.062, 1.813]	1.355* [1.031, 1.781]	1.289 [0.978, 1.699]
**Socioeconomic deprivation**						
Quartile 1 (most advantaged, reference group)	1.000	1.000	1.000	1.000	1.000	1.000
Quartile 2	1.035 [0.874, 1.224]	1.039 [0.878, 1.229]	1.023 [0.865, 1.210]	1.039 [0.878, 1.229]	1.035 [0.875, 1.225]	1.020 [0.862, 1.207]
Quartile 3	1.050 [0.894, 1.233]	1.039 [0.884, 1.220]	1.012 [0.861, 1.191]	1.041 [0.885, 1.223]	1.028 [0.875, 1.207]	1.011 [0.860, 1.190]
Quartile 4 (least advantaged)	1.209* [1.038, 1.408]	1.164 [0.997, 1.358]	1.135 [0.962, 1.340]	1.158 [0.989, 1.355]	1.133 [0.968, 1.326]	1.114 [0.943, 1.316]
**Education level**						
College or university degree (reference group)	1.000	1.000	1.000	1.000	1.000	1.000
A levels/AS levels or equivalent	1.049 [0.867, 1.270]	1.057 [0.873, 1.279]	1.060 [0.877, 1.282]	1.048 [0.866, 1.269]	1.045 [0.862, 1.265]	1.043 [0.862, 1.262]
O levels/GCSEs/CSEs or equivalent	1.121 [0.971, 1.295]	1.135 [0.982, 1.311]	1.131 [0.976, 1.311]	1.132 [0.979, 1.309]	1.093 [0.945, 1.263]	1.092 [0.942, 1.266]
Others	1.310** [1.111, 1.544]	1.301** [1.104, 1.533]	1.236* [1.045, 1.461]	1.306** [1.107, 1.541]	1.257** [1.066, 1.482]	1.207* [1.019, 1.428]
None of the above	1.227** [1.055, 1.428]	1.230** [1.057, 1.430]	1.210* [1.032, 1.419]	1.228** [1.053, 1.432]	1.188* [1.018, 1.386]	1.180* [1.005, 1.385]

Note: RRs shown are for the relationship between each variable shown and the risk of testing positive amongst those who have had a test. Coefficients for the Chinese, mixed and other groups and for the covariates included are not shown. *RR* risk ratio. 95% confidence intervals in brackets

Model 1: Adjusted for age, sex, assessment centre

Model 2: 1 + ethnicity, country of birth

Model 3: 2 + education level, household size, socioeconomic deprivation, housing tenure, urbanicity, employment status, manual occupation, healthcare worker

Model 4: 2 + longstanding illness/disability, number of chronic conditions, self-rated health

Model 5: 2 + body mass index, smoking status, alcohol consumption

Model 6: All of the above covariates

*p < 0.05, **p < 0.01, ***p < 0.001

**Fig. 2:**
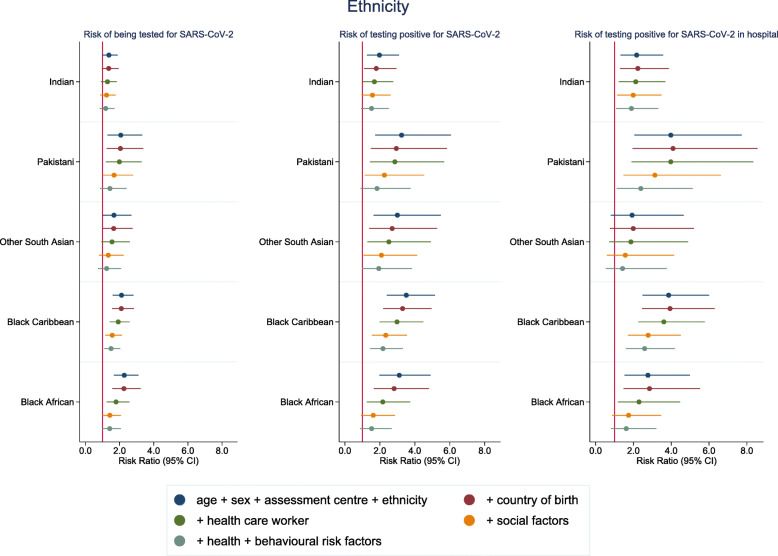
Risk ratios for associations between narrow ethnicity groups (white British as the reference category) and SARS-CoV-2. Model 1: age, sex and assessment centre. Model 2: model 1 + country of birth. Model 3: model 2 + healthcare worker. Model 4: model 3 + social variables (urbanicity, number of people per household, highest education level, deprivation, tenure status, employment status, manual work). Model 5: model 4 + health status variables (self-rated health, number of chronic conditions and longstanding illness) + behavioural risk factors (smoking, alcohol consumption and BMI). Coefficients for the white Irish, white other, mixed, Chinese, black other and other groups are not shown

When using a more detailed ethnicity classification within the south Asian and black groups, we observed important heterogeneity in the pattern of findings between the Indian group and other south Asian groups (Fig. 2 and Additional file Tables S7-S9). Compared to white British, risks were largest in the Pakistani group (RR 3.24 (95% CI 1.73–6.07)), followed by other south Asians (RR 3.00 (95% CI 1.64–5.49)) and were more modestly increased in the Indian group (RR 1.98 (95% CI 1.26–3.09)). There were less clear differences in the estimates for black Caribbeans and black Africans: RR 3.51 (95% CI 2.39–5.15) and RR 3.11 (95% CI 1.97–4.91) in initial models and RR 2.18 (95% CI 1.43–3.32) and RR 1.53 (95% CI 0.87–2.69) in fully adjusted models respectively.

**Fig. 3:**
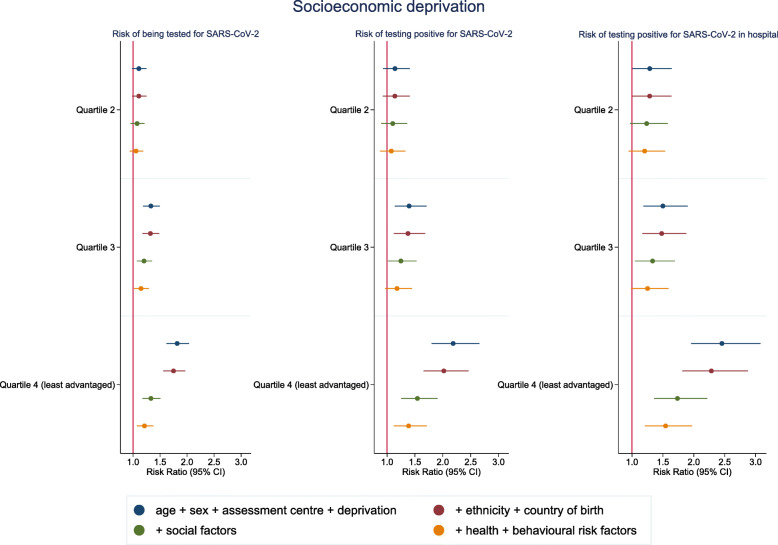
Risk ratios for associations between Townsend deprivation score quartile (most advantaged as reference category) and SARS-CoV-2. Model 1: age, sex and assessment centre. Model 2: model 1 + ethnicity + country of birth. Model 3: model 2 + social variables (healthcare worker, urbanicity, number of people per household, highest education level, tenure status, employment status, manual work). Model 4: model 3 + health status variables (self-rated health, number of chronic conditions and longstanding illness) + behavioural risk factors (smoking, alcohol consumption and BMI)

In comparison to the most socioeconomically advantaged quartile, living in a disadvantaged area (according to the Townsend deprivation score) was associated with a higher risk of confirmed infection, particularly for the most disadvantaged quartile (RR 2.19 (95% CI 1.80–2.66)) (Fig. 3 and Additional file: Table S10). Differences in ethnicity and country of birth, social factors, baseline health and behavioural risk factors all moderately attenuated the association in the most disadvantaged quartile. Socioeconomic deprivation was also associated with hospital cases (Additonal file: Table S11). While testing was again more likely, the risk of being diagnosed positive amongst those tested also tended to be higher, rather than lower (Table 2 and Additional file: Table S17).

**Fig. 4:**
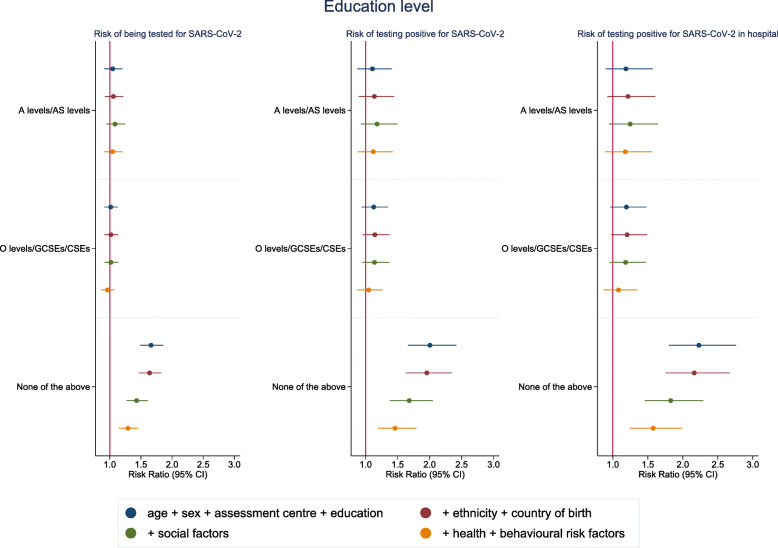
Risk ratios for associations between highest educational level (degree educated as reference category) and SARS-CoV-2. Model 1: age, sex and assessment centre. Model 2: model 1 + ethnicity + country of birth. Model 3: model 2 + social variables (healthcare worker, urbanicity, number of people per household, deprivation, tenure status, employment status, manual work). Model 4: model 3 + health status variables (self-rated health, number of chronic conditions and longstanding illness) + behavioural risk factors (smoking, alcohol consumption and BMI). Coefficient for the other groups are not shown

Analyses by education level also showed a higher risk of confirmed SARS-CoV-2 infection with the lowest level of education (RR 2.00 (95% CI 1.66–2.42) for no qualifications compared to degree level educated) (Fig. 4 and Additional file: Table S13). While adjustment for ethnicity and country of birth made little difference to the association, adjustment for social factors, baseline health and behavioural risk factors all attenuated the association somewhat (RR 1.46 (95% CI 1.19–1.79) in fully adjusted model). We again observed a similar pattern in hospital cases and found little evidence of increased testing amongst the less educated groups (Fig. 4 and Additional file Tables S14 and S18).

## Discussion

Several ethnic minority groups had a higher risk of both being diagnosed and testing positive in a hospital setting with laboratory-confirmed SARS-CoV-2 infection in the UK Biobank study. The black and south Asian groups were found to be at greatest risk, with Pakistani ethnicity at greatest risk within the south Asian group. Similarly, measures of socioeconomic disadvantage (area-based deprivation and lower education) were also associated with an increased risk of having confirmed infection and being a hospital case. For both ethnicity and socioeconomic position, we did not find evidence that these patterns were likely to be due to differential ascertainment, since although the likelihood of testing was increased, the likelihood of a positive test was, if anything, higher amongst ethnic minorities who had been tested. Ethnic differences in infection risk did not appear to be fully accounted for by differences in pre-existing health, behavioural risk factors or country of birth measured at baseline. Furthermore, socioeconomic differences appeared to make a moderate contribution to these ethnic differences.

Our study has several important strengths. First, by using a well-characterised cohort study, we can identify a clearly defined population at risk of experiencing SARS-CoV-2 infection. By combining data linkage with a large sample size, this has allowed us to provide empirical data from this pandemic in a timely fashion. Ethnicity was collected using self-report which is widely considered to be a gold standard approach [20], and the availability of a large dataset has allowed us to provide empirical data on this crucial policy priority in a timely fashion, including a more nuanced appreciation of the risks of infection within different members of the white majority population, as well as drilling down into more specific minority ethnic groups [21]. Our investigation of socioeconomic position has similarly benefited from being able to study different measures and assess the pattern of findings across these. The detailed data collected in this cohort has also allowed us to investigate the extent to which observed inequalities are potentially mediated by a wide range of factors, including behavioural risk factors, pre-existing health status and other social variables.

However, several potential limitations should be noted. Ascertainment bias is potentially problematic and could arise in several ways, including differential healthcare seeking, differential testing and differential prognosis. Even so, we have been unable to find any evidence to suggest that differential healthcare seeking or testing would explain the observed pattern of findings. Increased ascertainment amongst ethnic minorities would be expected to result in a lower proportion of confirmed cases amongst those tested whereas we observed the opposite. One possibility that remains is that some ethnic and socioeconomic groups have a poorer prognosis and are therefore more likely to be admitted to hospital and therefore to be tested [7]. However, if this were the case, the issue of more adverse outcomes amongst these groups remains concerning. Other limitations include the non-representativeness of the UK Biobank study population, potentially exacerbated by missing data, with those who were more advantaged being more likely to participate and ethnic minorities less well represented. There is therefore the potential that the findings in our study may not reflect the broader UK population [22, 23]. However, empirical research has found that this may not result in substantial bias in measures of association in the UK Biobank study [24]. Furthermore, estimates from other sources of inequalities in COVID-19 mortality show similar patterns of associations to our results [25, 26]. We have also been unable to fully exclude all deaths that occurred prior to the pandemic, due to lack of up-to-date linkage to mortality records at present. Our exposure data were collected some years ago, and it is therefore likely that pre-existing health, risk factors and some social variables have changed, although generally most risk factors track throughout life [27]. However, it is possible that management for chronic health conditions could have been differential across ethnic and socioeconomic groups [28] between baseline data collection and the pandemic period. Being a healthcare worker was also ascertained at baseline, although many who stopped employment in this area have now returned to work [29]. Lastly, due to sparse data, we have not explored the role of specific health conditions such as asthma, diabetes and high blood pressure, which have been shown to be associated with a higher risk of severe outcomes [3, 30] and are more prevalent amongst socioeconomically disadvantaged groups and some ethnic minority groups [31, 32]. However, these are likely to operate as mediators rather than confounders.

Administrative data from health services has recently suggested an increased risk of severe COVID-19 disease within ethnic minority groups. The UK’s Intensive Care National Audit & Research Centre (ICNARC) analysed data on 5578 patients admitted to critical care up to 16th April 2020 and found black and Asian people comprised a high proportion of total patients (11.2% and 14.9% respectively), although it was unclear whether these higher percentages were biased by most cases being initially seen in areas with high proportions of ethnic minority groups [33]. Similarly, data from the US Centers for Disease Control and Prevention also suggest a higher risk amongst black or African American people, but information on race was missing for approximately two-thirds of those diagnosed [34]. Analyses of administrative UK data have also suggested increased COVID-19 mortality in black and south Asian ethnic groups [26], which was only partly accounted for by socioeconomic differences [25]. However, the role of prior health and risk factors was not accounted for. Academic research on this topic has been limited to date. An ecological study of US counties has suggested that more socially vulnerable areas (which included greater numbers of people with socioeconomic disadvantage and ethnic minorities) were associated with higher COVID-19 case fatality rates [35]. Our study adds substantially to the evidence by finding that ethnicity appears to be an important predictor of laboratory-confirmed SARS-CoV-2 infection that is only partly attenuated by a large range of potential mediators (such as socioeconomic position), as well as addressing concerns about numerator-denominator bias.

Our results suggest there is an urgent need for further research on how SARS-CoV-2 infection affects different ethnic and socioeconomic groups. Our findings warrant replication in other datasets, ideally including representative samples and across different countries. As the pandemic evolves, there is a need to monitor infection and disease outcomes by ethnicity and socioeconomic position. However, data to allow this disaggregation is often not available—record linkage could potentially help address this gap, particularly in settings where administrative register data are available. Given the differences in health risks across occupational groups [36], understanding the risks that the full range of key workers experience is also required. Lastly, other social groups, such as homeless people, prisoners and undocumented migrants, experience severe disadvantage and research is necessary to study these highly vulnerable populations too [37, 38].

## Conclusions

The limited evidence available suggests that some ethnic minority groups, particularly black and south Asian people, are particularly vulnerable to the adverse consequences of COVID-19. Socioeconomic disadvantage and poorer pre-existing health do not explain all of this elevated risk. There is therefore a need to determine why this increased risk occurs. An immediate policy response is required to ensure the health system is responsive to the needs of ethnic minority groups. This should include ensuring that health and care workforces, which often rely on workers from minority ethnic populations, have access to the necessary personal protective equipment (PPE) to ensure they can work safely. Timely communication of guidelines to reduce the risk of being exposed to the virus is also required in a range of languages [39]. Previous evidence suggests ethnic minorities in the UK tend to receive reasonably equitable care in many, but not all, areas [40]. However, this is not the case in many other countries (such as the USA) where the adverse consequences of SARS-CoV-2 infection may be even worse. SARS-CoV-2 therefore has the potential to substantially exacerbate ethnic and socioeconomic inequalities in health [41], unless steps are taken to mitigate these inequalities. The data from this study may be helpful to inform allocation of more aggressive therapies in people with severe disease, or targeting preventative vaccination to at-risk groups, once evidence for such approaches becomes available.

## Supplementary information

**Additional file 1 : Figure S1**. Flowchart of study participants. **Table S1**. Missing data by ethnicity, socioeconomic deprivation and education level. **Table S2**. Description of the sample by ethnicity. **Table S3**. Description of SARS-CoV-2 test results within UK Biobank by ethnicity and socioeconomic position. **Table S4**. Ethnicity and risk of testing positive. **Table S5**. Ethnicity and risk of testing positive in hospital. **Table S6**. Ethnicity and risk of being tested. **Table S7**. Ethnicity (more defined groups) and risk of testing positive. **Table S8**. Ethnicity (more defined groups) and risk of testing positive in hospital. **Table S9**. Ethnicity (more defined groups) and risk of being tested. **Table S10**. Socioeconomic deprivation and risk of testing positive. **Table S11**. Socioeconomic deprivation and risk of testing positive in hospital. **Table S12**. Socioeconomic deprivation and risk of being tested. **Table S13**. Education level and risk of testing positive. **Table S14**. Education level and risk of testing positive in hospital. **Table S15**. Education level and risk of being tested. **Table S16**. Ethnicity and risk of testing positive amongst those tested. **Table S17**. Socioeconomic deprivation and risk of testing positive amongst those tested. **Table S18**. Education level and risk of testing positive amongst those tested.

## Data Availability

The data that support the findings of this study are available from UK Biobank (https://www.ukbiobank.ac.uk/), but restrictions apply to their availability. These data were used under licence for the current study and so are not publicly available. The data are available from the authors upon reasonable request and with permission of UK Biobank.

## Abbreviations

BMI: Body mass index
CI: Confidence interval
COVID-19: Coronavirus disease 2019
CSE: Certificate of Secondary Education
GCSE: General Certificate of Secondary Education
ICNARC: Intensive Care National Audit & Research Centre
NHS: National Health Service
PPE: Personal protective equipment
RR: Risk ratio
SARS-CoV-2: Severe acute respiratory syndrome coronavirus-2
SOC: Standard Occupational Classification
UK: United Kingdom
