# Supplementary material for: Ethnic and socioeconomic differences in SARS-CoV-2 infection: prospective cohort study using UK Biobank
Source: BMC Med. 2020 May 29;18:160. doi: 10.1186/s12916-020-01640-8 (PMC7255908; doi:10.1186/s12916-020-01640-8)
Supplement: Supplementary file 1 — Additional file 1 : Figure S1. Flowchart of study participants. Table S1. Missing data by ethnicity, socioeconomic deprivation and education level. Table S2. Description of the sample by ethnicity. Table S3. Description of SARS-CoV-2 test results within UK Biobank by ethnicity and socioeconomic position. Table S4. Ethnicity and risk of testing positive. Table S5. Ethnicity and risk of testing positive in hospital. Table S6. Ethnicity and risk of being tested. Table S7. Ethnicity (more defined groups) and risk of testing positive. Table S8. Ethnicity (more defined groups) and risk of testing positive in hospital. Table S9. Ethnicity (more defined groups) and risk of being tested. Table S10. Socioeconomic deprivation and risk of testing positive. Table S11. Socioeconomic deprivation and risk of testing positive in hospital. Table S12. Socioeconomic deprivation and risk of being tested. Table S13. Education level and risk of testing positive. Table S14. Education level and risk of testing positive in hospital. Table S15. Education level and risk of being tested. Table S16. Ethnicity and risk of testing positive amongst those tested. Table S17. Socioeconomic deprivation and risk of testing positive amongst those tested. Table S18. Education level and risk of testing positive amongst those tested. [file 12916_2020_1640_MOESM1_ESM.docx]

**Additional file 1**

**Figures:**

[Figure S1: Flowchart of study participants 2](file:///K:\Fellowship\COVID\UKB\Ethnicity_Update\11thMay2020\Additional%20file%201_update_11thMay20.docx#_Toc40102742)

**Tables:**

[Table S1: Missing data by ethnicity, socioeconomic deprivation and education level 3](#_Toc40102724)

[Table S2: Description of the sample by ethnicity 4](#_Toc40102725)

[Table S3: Description of SARS-CoV-2 test results within UK Biobank by ethnicity and socioeconomic position 8](#_Toc40102726)

[Table S4: Ethnicity and risk of testing positive 9](#_Toc40102727)

[Table S5: Ethnicity and risk of testing positive in hospital 14](#_Toc40102728)

[Table S6: Ethnicity and risk of being tested 19](#_Toc40102729)

[Table S7: Ethnicity (more defined groups) and risk of testing positive 24](#_Toc40102730)

[Table S8: Ethnicity (more defined groups) and risk of testing positive in hospital 29](#_Toc40102731)

[Table S9: Ethnicity (more defined groups) and risk of being tested 34](#_Toc40102732)

[Table S10: Socioeconomic deprivation and risk of testing positive 39](#_Toc40102733)

[Table S11: Socioeconomic deprivation and risk of testing positive in hospital 43](#_Toc40102734)

[Table S12: Socioeconomic deprivation and risk of being tested 47](#_Toc40102735)

[Table S13: Education level and risk of testing positive 51](#_Toc40102736)

[Table S14: Education level and risk of testing positive in hospital 55](#_Toc40102737)

[Table S15: Education level and risk of being tested 59](#_Toc40102738)

[Table S16: Ethnicity and risk of testing positive amongst those tested 63](#_Toc40102739)

[Table S17: Socioeconomic deprivation and risk of testing positive amongst those tested 68](#_Toc40102740)

[Table S18: Education level and risk of testing positive amongst those tested 72](#_Toc40102741)

**Note:**

Cells with 1.000 and no 95% confidence interval refer to reference categories.

Models containing health care worker status and manual occupation which do not show coefficients for those not in employment have been dropped due to collinearity. Number of observations (N) and percentages (%) and Risk Ratios (RR) and 95% confidence intervals (CI) presented in tables.

^*^ *p* < 0.05, ^**^ *p* < 0.01, ^***^ *p* < 0.001

Figure S1: Flowchart of study participants

UK Biobank participants at baseline
(n = 502,536)

Participants who have withdrawn
(n =30)

Eligible UK Biobank participants

(n = 502,506)

Participants who attended assessment centres in Scotland and Wales
(n = 56,649)

Eligible UK Biobank participants in England

(n = 445,857)

Participants known to have died up to 31 January 2018
(n = 17,632)

Eligible UK Biobank participants in England known to be alive

(n = 428,225)

Eligible UK Biobank participants in England known to be alive with complete data

(n =392,116)

Participants with missing data for any variable
(n =36,109)

Table S1: Missing data by ethnicity, socioeconomic deprivation and education level

|  | Not missing | | Missing | | Total | |
| --- | --- | --- | --- | --- | --- | --- |
| Ethnicity | **N** | **%** | **N** | **%** | **N** | **%** |
| White British | 348,735 | 92.9 | 26,631 | 7.1 | 375,366 | 100 |
| White Irish | 9,800 | 90.6 | 1,022 | 9.4 | 10,822 | 100 |
| White Other | 12,925 | 90.0 | 1,440 | 10.0 | 14,365 | 100 |
| Mixed | 2,356 | 88.5 | 305 | 11.5 | 2,661 | 100 |
| Indian | 4,571 | 81.4 | 1,047 | 18.6 | 5,618 | 100 |
| Pakistani | 1,259 | 75.3 | 413 | 24.7 | 1,672 | 100 |
| Other South Asian | 1,493 | 76.3 | 463 | 23.7 | 1,956 | 100 |
| Black Caribbean | 3,669 | 84.4 | 677 | 15.6 | 4,346 | 100 |
| Black African | 2,623 | 81.0 | 615 | 19.0 | 3,238 | 100 |
| Black Other | 103 | 72.5 | 39 | 27.5 | 142 | 100 |
| Chinese | 1,153 | 82.2 | 249 | 17.8 | 1,402 | 100 |
| Other | 3,429 | 81.6 | 775 | 18.4 | 4,204 | 100 |
| Missing |  |  | 2,433 | 100.0 | 2,433 | 100 |
| Total | 392,116 | 91.6 | 36,109 | 8.4 | 428,225 | 100 |
| Deprivation quartile |  |  |  |  |  |  |
| Quartile 1 (most advantaged) | 100,701 | 93.6 | 6,924 | 6.4 | 107,625 | 100 |
| Quartile 2 | 99,838 | 93.0 | 7,556 | 7.0 | 107,394 | 100 |
| Quartile 3 | 98,380 | 91.9 | 8,642 | 8.1 | 107,022 | 100 |
| Quartile 4 (least advantaged) | 93,197 | 88.2 | 12,486 | 11.8 | 105,683 | 100 |
| Missing |  |  | 501 | 100.0 | 501 | 100 |
| Total | 392,116 | 91.6 | 36,109 | 8.4 | 428,225 | 100 |
| Education level |  |  |  |  |  |  |
| College or University degree | 128,890 | 94.2 | 7,976 | 5.8 | 136,866 | 100 |
| A levels/AS levels | 44,650 | 94.2 | 2,731 | 5.8 | 47,381 | 100 |
| O levels/GCSEs/CSEs | 108,648 | 93.8 | 7,141 | 6.2 | 115,789 | 100 |
| Other | 46,393 | 93.4 | 3,296 | 6.6 | 49,689 | 100 |
| None of the above | 63,535 | 91.3 | 6,017 | 8.7 | 69,552 | 100 |
| Missing |  |  | 8,948 | 100.0 | 8,948 | 100 |
| Total | 392,116 | 91.6 | 36,109 | 8.4 | 428,225 | 100 |

**Note:** Model 1 for regression analyses for testing positive and testing positive as a hospital case were repeated for all available data (i.e. exposure, age, sex and assessment centre), rather than complete cases only. Findings were similar (not shown).

**Table S2:** **Description of the sample by ethnicity**

|  | White British | White British | White Irish | White Irish | White Other | White Other | Mixed | Mixed | South Asian | South Asian | Black | Black | Chinese | Chinese | Other | Other | Total | Total |
| --- | --- | --- | --- | --- | --- | --- | --- | --- | --- | --- | --- | --- | --- | --- | --- | --- | --- | --- |
|  | N | % | N | % | N | % | N | % | N | % | N | % | N | % | N | % | N | % |
| Age group |  |  |  |  |  |  |  |  |  |  |  |  |  |  |  |  |  |  |
| 40-44 | 33,707 | 82.2 | 1,043 | 2.5 | 2,074 | 5.1 | 555 | 1.4 | 1,408 | 3.4 | 1,349 | 3.3 | 194 | 0.5 | 665 | 1.6 | 40,995 | 100 |
| 45-49 | 44,112 | 84.6 | 1,420 | 2.7 | 2,130 | 4.1 | 568 | 1.1 | 1,375 | 2.6 | 1,556 | 3.0 | 256 | 0.5 | 699 | 1.3 | 52,116 | 100 |
| 50-54 | 52,539 | 87.1 | 1,626 | 2.7 | 2,147 | 3.6 | 425 | 0.7 | 1,339 | 2.2 | 1,326 | 2.2 | 227 | 0.4 | 662 | 1.1 | 60,291 | 100 |
| 55-59 | 63,700 | 89.5 | 1,845 | 2.6 | 2,354 | 3.3 | 336 | 0.5 | 1,208 | 1.7 | 878 | 1.2 | 241 | 0.3 | 598 | 0.8 | 71,160 | 100 |
| 60-64 | 88,423 | 92.5 | 2,076 | 2.2 | 2,441 | 2.6 | 282 | 0.3 | 1,082 | 1.1 | 666 | 0.7 | 145 | 0.2 | 489 | 0.5 | 95,604 | 100 |
| 65-69 | 64,563 | 92.1 | 1,752 | 2.5 | 1,735 | 2.5 | 184 | 0.3 | 884 | 1.3 | 597 | 0.9 | 87 | 0.1 | 308 | 0.4 | 70,110 | 100 |
| 70+ | 1,691 | 91.9 | 38 | 2.1 | 44 | 2.4 | 6 | 0.3 | 27 | 1.5 | 23 | 1.3 | 3 | 0.2 | 8 | 0.4 | 1,840 | 100 |
| Sex |  |  |  |  |  |  |  |  |  |  |  |  |  |  |  |  |  |  |
| Female | 190,775 | 88.6 | 5,180 | 2.4 | 7,998 | 3.7 | 1,493 | 0.7 | 3,449 | 1.6 | 3,755 | 1.7 | 733 | 0.3 | 1,968 | 0.9 | 215,351 | 100 |
| Male | 157,960 | 89.4 | 4,620 | 2.6 | 4,927 | 2.8 | 863 | 0.5 | 3,874 | 2.2 | 2,640 | 1.5 | 420 | 0.2 | 1,461 | 0.8 | 176,765 | 100 |
| Country of birth |  |  |  |  |  |  |  |  |  |  |  |  |  |  |  |  |  |  |
| UK & Ireland | 342,178 | 94.8 | 9,610 | 2.7 | 4,236 | 1.2 | 1,616 | 0.4 | 755 | 0.2 | 1,859 | 0.5 | 100 | 0.0 | 671 | 0.2 | 361,025 | 100 |
| Elsewhere | 6,557 | 21.1 | 190 | 0.6 | 8,689 | 27.9 | 740 | 2.4 | 6,568 | 21.1 | 4,536 | 14.6 | 1,053 | 3.4 | 2,758 | 8.9 | 31,091 | 100 |
| Number in household |  |  |  |  |  |  |  |  |  |  |  |  |  |  |  |  |  |  |
| 1 | 61,249 | 87.7 | 2,392 | 3.4 | 2,621 | 3.8 | 543 | 0.8 | 686 | 1.0 | 1,560 | 2.2 | 161 | 0.2 | 650 | 0.9 | 69,862 | 100 |
| 2 | 169,934 | 92.5 | 3,778 | 2.1 | 5,008 | 2.7 | 760 | 0.4 | 1,556 | 0.8 | 1,508 | 0.8 | 348 | 0.2 | 885 | 0.5 | 183,777 | 100 |
| 3 | 55,015 | 87.4 | 1,600 | 2.5 | 2,226 | 3.5 | 464 | 0.7 | 1,379 | 2.2 | 1,314 | 2.1 | 285 | 0.5 | 651 | 1.0 | 62,934 | 100 |
| 4+ | 62,537 | 82.8 | 2,030 | 2.7 | 3,070 | 4.1 | 589 | 0.8 | 3,702 | 4.9 | 2,013 | 2.7 | 359 | 0.5 | 1,243 | 1.6 | 75,543 | 100 |
| Education level |  |  |  |  |  |  |  |  |  |  |  |  |  |  |  |  |  |  |
| College or University degree | 109,271 | 84.8 | 3,496 | 2.7 | 7,603 | 5.9 | 952 | 0.7 | 3,084 | 2.4 | 2,223 | 1.7 | 593 | 0.5 | 1,668 | 1.3 | 128,890 | 100 |
| A levels/AS levels | 39,905 | 89.4 | 1,241 | 2.8 | 1,652 | 3.7 | 295 | 0.7 | 686 | 1.5 | 441 | 1.0 | 111 | 0.2 | 319 | 0.7 | 44,650 | 100 |
| O levels/GCSEs/CSEs | 100,241 | 92.3 | 2,106 | 1.9 | 1,600 | 1.5 | 600 | 0.6 | 1,671 | 1.5 | 1,702 | 1.6 | 195 | 0.2 | 533 | 0.5 | 108,648 | 100 |
| Other | 41,161 | 88.7 | 1,287 | 2.8 | 1,129 | 2.4 | 249 | 0.5 | 742 | 1.6 | 1,205 | 2.6 | 129 | 0.3 | 491 | 1.1 | 46,393 | 100 |
| None of the above | 58,157 | 91.5 | 1,670 | 2.6 | 941 | 1.5 | 260 | 0.4 | 1,140 | 1.8 | 824 | 1.3 | 125 | 0.2 | 418 | 0.7 | 63,535 | 100 |
| Deprivation quartile |  |  |  |  |  |  |  |  |  |  |  |  |  |  |  |  |  |  |
| Quartile 1 (most advantaged) | 94,661 | 94.0 | 1,887 | 1.9 | 2,139 | 2.1 | 323 | 0.3 | 858 | 0.9 | 249 | 0.2 | 229 | 0.2 | 355 | 0.4 | 100,701 | 100 |
| Quartile 2 | 92,795 | 92.9 | 2,067 | 2.1 | 2,447 | 2.5 | 394 | 0.4 | 1,062 | 1.1 | 459 | 0.5 | 229 | 0.2 | 385 | 0.4 | 99,838 | 100 |
| Quartile 3 | 87,393 | 88.8 | 2,630 | 2.7 | 3,314 | 3.4 | 564 | 0.6 | 2,172 | 2.2 | 1,232 | 1.3 | 307 | 0.3 | 768 | 0.8 | 98,380 | 100 |
| Quartile 4 (least advantaged) | 73,886 | 79.3 | 3,216 | 3.5 | 5,025 | 5.4 | 1,075 | 1.2 | 3,231 | 3.5 | 4,455 | 4.8 | 388 | 0.4 | 1,921 | 2.1 | 93,197 | 100 |
| Housing tenure |  |  |  |  |  |  |  |  |  |  |  |  |  |  |  |  |  |  |
| Own | 318,581 | 90.5 | 8,414 | 2.4 | 10,481 | 3.0 | 1,766 | 0.5 | 6,018 | 1.7 | 3,699 | 1.1 | 974 | 0.3 | 2,146 | 0.6 | 352,079 | 100 |
| Rent/Other | 30,154 | 75.3 | 1,386 | 3.5 | 2,444 | 6.1 | 590 | 1.5 | 1,305 | 3.3 | 2,696 | 6.7 | 179 | 0.4 | 1,283 | 3.2 | 40,037 | 100 |
|  |  |  |  |  |  |  |  |  |  |  |  |  |  |  |  |  |  |  |
| Urban/rural |  |  |  |  |  |  |  |  |  |  |  |  |  |  |  |  |  |  |
| Urban | 293,890 | 87.8 | 9,033 | 2.7 | 11,652 | 3.5 | 2,168 | 0.6 | 7,115 | 2.1 | 6,321 | 1.9 | 1,099 | 0.3 | 3,292 | 1.0 | 334,570 | 100 |
| Rural | 54,845 | 95.3 | 767 | 1.3 | 1,273 | 2.2 | 188 | 0.3 | 208 | 0.4 | 74 | 0.1 | 54 | 0.1 | 137 | 0.2 | 57,546 | 100 |
| Employment status |  |  |  |  |  |  |  |  |  |  |  |  |  |  |  |  |  |  |
| In paid employment or self-employed | 201,860 | 87.7 | 5,963 | 2.6 | 8,634 | 3.8 | 1,676 | 0.7 | 4,705 | 2.0 | 4,430 | 1.9 | 804 | 0.3 | 2,118 | 0.9 | 230,190 | 100 |
| Retired | 119,745 | 93.1 | 2,792 | 2.2 | 2,777 | 2.2 | 326 | 0.3 | 1,389 | 1.1 | 857 | 0.7 | 195 | 0.2 | 532 | 0.4 | 128,613 | 100 |
| Looking after home and/or family | 9,150 | 83.5 | 241 | 2.2 | 618 | 5.6 | 91 | 0.8 | 411 | 3.8 | 144 | 1.3 | 86 | 0.8 | 215 | 2.0 | 10,956 | 100 |
| Unable to work because of sickness or disability | 9,420 | 84.8 | 437 | 3.9 | 308 | 2.8 | 107 | 1.0 | 333 | 3.0 | 325 | 2.9 | 10 | 0.1 | 171 | 1.5 | 11,111 | 100 |
| Unemployed | 4,834 | 75.7 | 232 | 3.6 | 294 | 4.6 | 88 | 1.4 | 301 | 4.7 | 401 | 6.3 | 36 | 0.6 | 200 | 3.1 | 6,386 | 100 |
| Other | 3,726 | 76.7 | 135 | 2.8 | 294 | 6.0 | 68 | 1.4 | 184 | 3.8 | 238 | 4.9 | 22 | 0.5 | 193 | 4.0 | 4,860 | 100 |
| Manual occupation |  |  |  |  |  |  |  |  |  |  |  |  |  |  |  |  |  |  |
| Non-manual | 175,036 | 87.7 | 5,202 | 2.6 | 7,744 | 3.9 | 1,445 | 0.7 | 3,961 | 2.0 | 3,717 | 1.9 | 710 | 0.4 | 1,749 | 0.9 | 199,564 | 100 |
| Manual | 26,824 | 87.6 | 761 | 2.5 | 890 | 2.9 | 231 | 0.8 | 744 | 2.4 | 713 | 2.3 | 94 | 0.3 | 369 | 1.2 | 30,626 | 100 |
| Not in employment | 146,875 | 90.7 | 3,837 | 2.4 | 4,291 | 2.6 | 680 | 0.4 | 2,618 | 1.6 | 1,965 | 1.2 | 349 | 0.2 | 1,311 | 0.8 | 161,926 | 100 |
| Healthcare worker status |  |  |  |  |  |  |  |  |  |  |  |  |  |  |  |  |  |  |
| Non-healthcare worker | 180,327 | 88.3 | 5,076 | 2.5 | 7,610 | 3.7 | 1,441 | 0.7 | 4,067 | 2.0 | 3,373 | 1.7 | 680 | 0.3 | 1,680 | 0.8 | 204,254 | 100 |
| Healthcare worker | 21,533 | 83.0 | 887 | 3.4 | 1,024 | 3.9 | 235 | 0.9 | 638 | 2.5 | 1,057 | 4.1 | 124 | 0.5 | 438 | 1.7 | 25,936 | 100 |
| Not in employment | 146,875 | 90.7 | 3,837 | 2.4 | 4,291 | 2.6 | 680 | 0.4 | 2,618 | 1.6 | 1,965 | 1.2 | 349 | 0.2 | 1,311 | 0.8 | 161,926 | 100 |
| Long-standing illness, disability or infirmity |  |  |  |  |  |  |  |  |  |  |  |  |  |  |  |  |  |  |
| No | 239,129 | 88.9 | 6,590 | 2.5 | 9,196 | 3.4 | 1,640 | 0.6 | 5,022 | 1.9 | 4,140 | 1.5 | 927 | 0.3 | 2,275 | 0.8 | 268,919 | 100 |
| Yes | 109,606 | 89.0 | 3,210 | 2.6 | 3,729 | 3.0 | 716 | 0.6 | 2,301 | 1.9 | 2,255 | 1.8 | 226 | 0.2 | 1,154 | 0.9 | 123,197 | 100 |
| Number of chronic conditions |  |  |  |  |  |  |  |  |  |  |  |  |  |  |  |  |  |  |
| 0 | 130,612 | 88.3 | 3,641 | 2.5 | 5,482 | 3.7 | 980 | 0.7 | 2,764 | 1.9 | 2,453 | 1.7 | 618 | 0.4 | 1,393 | 0.9 | 147,943 | 100 |
| 1 | 115,851 | 89.1 | 3,237 | 2.5 | 4,294 | 3.3 | 737 | 0.6 | 2,311 | 1.8 | 2,138 | 1.6 | 335 | 0.3 | 1,131 | 0.9 | 130,034 | 100 |
| 2 | 61,908 | 89.4 | 1,757 | 2.5 | 2,013 | 2.9 | 406 | 0.6 | 1,326 | 1.9 | 1,128 | 1.6 | 147 | 0.2 | 537 | 0.8 | 69,222 | 100 |
| 3 | 25,995 | 89.8 | 745 | 2.6 | 746 | 2.6 | 145 | 0.5 | 597 | 2.1 | 446 | 1.5 | 41 | 0.1 | 242 | 0.8 | 28,957 | 100 |
| 4+ | 14,369 | 90.0 | 420 | 2.6 | 390 | 2.4 | 88 | 0.6 | 325 | 2.0 | 230 | 1.4 | 12 | 0.1 | 126 | 0.8 | 15,960 | 100 |
| Overall health rating |  |  |  |  |  |  |  |  |  |  |  |  |  |  |  |  |  |  |
| Excellent | 59,116 | 90.2 | 1,628 | 2.5 | 2,490 | 3.8 | 354 | 0.5 | 678 | 1.0 | 729 | 1.1 | 144 | 0.2 | 421 | 0.6 | 65,560 | 100 |
| Good | 207,745 | 89.7 | 5,576 | 2.4 | 7,401 | 3.2 | 1,309 | 0.6 | 3,703 | 1.6 | 3,414 | 1.5 | 692 | 0.3 | 1,832 | 0.8 | 231,672 | 100 |
| Fair | 68,834 | 86.8 | 2,098 | 2.6 | 2,512 | 3.2 | 567 | 0.7 | 2,297 | 2.9 | 1,818 | 2.3 | 289 | 0.4 | 932 | 1.2 | 79,347 | 100 |
| Poor | 13,040 | 83.9 | 498 | 3.2 | 522 | 3.4 | 126 | 0.8 | 645 | 4.2 | 434 | 2.8 | 28 | 0.2 | 244 | 1.6 | 15,537 | 100 |
| BMI Category |  |  |  |  |  |  |  |  |  |  |  |  |  |  |  |  |  |  |
| Underweight (<18.5) | 1,674 | 86.8 | 40 | 2.1 | 92 | 4.8 | 18 | 0.9 | 44 | 2.3 | 5 | 0.3 | 24 | 1.2 | 31 | 1.6 | 1,928 | 100 |
| Normal weight (18.5-24.9) | 115,279 | 88.8 | 3,203 | 2.5 | 5,079 | 3.9 | 857 | 0.7 | 2,419 | 1.9 | 1,185 | 0.9 | 718 | 0.6 | 1,015 | 0.8 | 129,755 | 100 |
| Overweight (25.0-29.9) | 149,104 | 89.3 | 4,186 | 2.5 | 4,996 | 3.0 | 907 | 0.5 | 3,327 | 2.0 | 2,646 | 1.6 | 339 | 0.2 | 1,474 | 0.9 | 166,979 | 100 |
| Obese (>=30.0) | 82,678 | 88.5 | 2,371 | 2.5 | 2,758 | 3.0 | 574 | 0.6 | 1,533 | 1.6 | 2,559 | 2.7 | 72 | 0.1 | 909 | 1.0 | 93,454 | 100 |
| Smoking status |  |  |  |  |  |  |  |  |  |  |  |  |  |  |  |  |  |  |
| Never | 192,324 | 88.5 | 4,535 | 2.1 | 6,174 | 2.8 | 1,139 | 0.5 | 5,621 | 2.6 | 4,514 | 2.1 | 916 | 0.4 | 2,074 | 1.0 | 217,297 | 100 |
| Previous | 123,422 | 90.4 | 3,954 | 2.9 | 5,085 | 3.7 | 784 | 0.6 | 1,033 | 0.8 | 1,130 | 0.8 | 162 | 0.1 | 912 | 0.7 | 136,482 | 100 |
| Current | 32,989 | 86.1 | 1,311 | 3.4 | 1,666 | 4.3 | 433 | 1.1 | 669 | 1.7 | 751 | 2.0 | 75 | 0.2 | 443 | 1.2 | 38,337 | 100 |
| Alcohol consumption |  |  |  |  |  |  |  |  |  |  |  |  |  |  |  |  |  |  |
| Daily or almost daily | 74,976 | 91.9 | 2,062 | 2.5 | 2,870 | 3.5 | 353 | 0.4 | 530 | 0.6 | 426 | 0.5 | 86 | 0.1 | 264 | 0.3 | 81,567 | 100 |
| Three or four times a week | 85,003 | 92.1 | 2,548 | 2.8 | 2,635 | 2.9 | 424 | 0.5 | 645 | 0.7 | 628 | 0.7 | 79 | 0.1 | 346 | 0.4 | 92,308 | 100 |
| Once or twice a week | 91,766 | 90.9 | 2,469 | 2.4 | 3,019 | 3.0 | 572 | 0.6 | 1,076 | 1.1 | 1,308 | 1.3 | 148 | 0.1 | 598 | 0.6 | 100,956 | 100 |
| One to three times a month | 38,925 | 89.0 | 971 | 2.2 | 1,554 | 3.6 | 349 | 0.8 | 569 | 1.3 | 866 | 2.0 | 127 | 0.3 | 382 | 0.9 | 43,743 | 100 |
| Special occasions only | 36,155 | 82.3 | 998 | 2.3 | 1,860 | 4.2 | 406 | 0.9 | 1,382 | 3.1 | 1,799 | 4.1 | 417 | 0.9 | 899 | 2.0 | 43,916 | 100 |
| Never (former drinker) | 11,286 | 84.8 | 447 | 3.4 | 433 | 3.3 | 114 | 0.9 | 413 | 3.1 | 362 | 2.7 | 41 | 0.3 | 219 | 1.6 | 13,315 | 100 |
| Never | 10,624 | 65.1 | 305 | 1.9 | 554 | 3.4 | 138 | 0.8 | 2,708 | 16.6 | 1,006 | 6.2 | 255 | 1.6 | 721 | 4.4 | 16,311 | 100 |
| Total | 348,735 | 88.9 | 9,800 | 2.5 | 12,925 | 3.3 | 2,356 | 0.6 | 7,323 | 1.9 | 6,395 | 1.6 | 1,153 | 0.3 | 3,429 | 0.9 | 392,116 | 100 |

Table S3: Description of SARS-CoV-2 test results within UK Biobank by ethnicity and socioeconomic position

|  | Tested for SARS-CoV-2 | | | | | Tested positive for SARS-CoV-2 | | | | | Tested positive for SARS-CoV-2 in hospital | | | | |
| --- | --- | --- | --- | --- | --- | --- | --- | --- | --- | --- | --- | --- | --- | --- | --- |
| Ethnicity | No | No | Yes | Yes | Total | No | No | Yes | Yes | Total | No | No | Yes | Yes | Total |
|  | N | % | N | % | N | N | % | N | % | N | N | % | N | % | N |
| White British | 346,470 | 99.4 | 2,265 | 0.6 | 348,735 | 347,967 | 99.8 | 768 | 0.2 | 348,735 | 348,141 | 99.8 | 594 | 0.2 | 348,735 |
| White Irish | 9,718 | 99.2 | 82 | 0.8 | 9,800 | 9,768 | 99.7 | 32 | 0.3 | 9,800 | 9,773 | 99.7 | 27 | 0.3 | 9,800 |
| White Other | 12,841 | 99.4 | 84 | 0.6 | 12,925 | 12,895 | 99.8 | 30 | 0.2 | 12,925 | 12,907 | 99.9 | 18 | 0.1 | 12,925 |
| Mixed | 2,338 | 99.2 | 18 | 0.8 | 2,356 | 2,350 | 99.7 | 6 | 0.3 | 2,356 | 2,351 | 99.8 | 5 | 0.2 | 2,356 |
| South Asian | 7,249 | 99.0 | 74 | 1.0 | 7,323 | 7,281 | 99.4 | 42 | 0.6 | 7,323 | 7,292 | 99.6 | 31 | 0.4 | 7,323 |
| Black | 6,301 | 98.5 | 94 | 1.5 | 6,395 | 6,345 | 99.2 | 50 | 0.8 | 6,395 | 6,360 | 99.5 | 35 | 0.5 | 6,395 |
| Chinese | 1,149 | 99.7 | 4 | 0.3 | 1,153 | 1,150 | 99.7 | 3 | 0.3 | 1,153 | 1,150 | 99.7 | 3 | 0.3 | 1,153 |
| Other | 3,392 | 98.9 | 37 | 1.1 | 3,429 | 3,412 | 99.5 | 17 | 0.5 | 3,429 | 3,416 | 99.6 | 13 | 0.4 | 3,429 |
| Socioeconomic deprivation |  |  |  |  |  |  |  |  |  |  |  |  |  |  |  |
| Quartile 1 (most advantaged) | 100,192 | 99.5 | 509 | 0.5 | 100,701 | 100,537 | 99.8 | 164 | 0.2 | 100,701 | 100,586 | 99.9 | 115 | 0.1 | 100,701 |
| Quartile 2 | 99,268 | 99.4 | 570 | 0.6 | 99,838 | 99,648 | 99.8 | 190 | 0.2 | 99,838 | 99,686 | 99.8 | 152 | 0.2 | 99,838 |
| Quartile 3 | 97,705 | 99.3 | 675 | 0.7 | 98,380 | 98,146 | 99.8 | 234 | 0.2 | 98,380 | 98,203 | 99.8 | 177 | 0.2 | 98,380 |
| Quartile 4 (least advantaged) | 92,293 | 99.0 | 904 | 1.0 | 93,197 | 92,837 | 99.6 | 360 | 0.4 | 93,197 | 92,915 | 99.7 | 282 | 0.3 | 93,197 |
| Education level |  |  |  |  |  |  |  |  |  |  |  |  |  |  |  |
| College or University degree | 128,124 | 99.4 | 766 | 0.6 | 128,890 | 128,642 | 99.8 | 248 | 0.2 | 128,890 | 128,714 | 99.9 | 176 | 0.1 | 128,890 |
| A levels/  AS levels | 44,381 | 99.4 | 269 | 0.6 | 44,650 | 44,559 | 99.8 | 91 | 0.2 | 44,650 | 44,581 | 99.8 | 69 | 0.2 | 44,650 |
| O levels/ GCSEs/CSEs | 108,006 | 99.4 | 642 | 0.6 | 108,648 | 108,420 | 99.8 | 228 | 0.2 | 108,648 | 108,475 | 99.8 | 173 | 0.2 | 108,648 |
| Other | 46,050 | 99.3 | 343 | 0.7 | 46,393 | 46,252 | 99.7 | 141 | 0.3 | 46,393 | 46,287 | 99.8 | 106 | 0.2 | 46,393 |
| None of the above | 62,897 | 99.0 | 638 | 1.0 | 63,535 | 63,295 | 99.6 | 240 | 0.4 | 63,535 | 63,333 | 99.7 | 202 | 0.3 | 63,535 |
| Total | 389,458 | 99.3 | 2,658 | 0.7 | 392,116 | 391,168 | 99.8 | 948 | 0.2 | 392,116 | 391,390 | 99.8 | 726 | 0.2 | 392,116 |

Table S4: Ethnicity and risk of testing positive

|  | **Model 1** | **Model 2** | **Model 3** | **Model 4** | **Model 5** | **Model 6** | **Model 7** |
| --- | --- | --- | --- | --- | --- | --- | --- |
| **Tested positive for SARS-CoV-2** | **RR**  **[95% CI]** | **RR**  **[95% CI]** | **RR**  **[95% CI]** | **RR**  **[95% CI]** | **RR**  **[95% CI]** | **RR**  **[95% CI]** | **RR**  **[95% CI]** |
| **Age group:** 40-44 | 1.000 - | 1.000 - | 1.000 - | 1.000 - | 1.000 - | 1.000 - | 1.000 - |
| 45-49 | 0.830 [0.652,1.056] | 0.829 [0.651,1.056] | 0.810 [0.636,1.031] | 0.818 [0.643,1.042] | 0.805 [0.632,1.025] | 0.827 [0.649,1.053] | 0.803 [0.631,1.021] |
| 50-54 | 0.616^***^ [0.477,0.795] | 0.614^***^ [0.475,0.792] | 0.601^***^ [0.465,0.775] | 0.618^***^ [0.480,0.797] | 0.573^***^ [0.444,0.739] | 0.599^***^ [0.464,0.774] | 0.583^***^ [0.452,0.751] |
| 55-59 | 0.587^***^ [0.457,0.754] | 0.584^***^ [0.454,0.751] | 0.567^***^ [0.441,0.730] | 0.615^***^ [0.478,0.792] | 0.526^***^ [0.410,0.673] | 0.561^***^ [0.436,0.721] | 0.564^***^ [0.439,0.724] |
| 60-64 | 0.685^**^ [0.545,0.861] | 0.683^**^ [0.543,0.859] | 0.629^***^ [0.497,0.797] | 0.723^*^ [0.563,0.929] | 0.606^***^ [0.482,0.762] | 0.643^***^ [0.510,0.812] | 0.657^**^ [0.510,0.847] |
| 65-69 | 1.218 [0.982,1.512] | 1.214 [0.977,1.508] | 1.052 [0.831,1.332] | 1.234 [0.938,1.621] | 1.049 [0.842,1.306] | 1.124 [0.901,1.403] | 1.102 [0.835,1.454] |
| 70+ | 2.213^**^ [1.227,3.991] | 2.203^**^ [1.221,3.975] | 1.889^*^ [1.039,3.432] | 2.206^*^ [1.197,4.066] | 1.901^*^ [1.050,3.440] | 2.033^*^ [1.124,3.678] | 1.991^*^ [1.075,3.687] |
| **Sex:** Female | 1.000 - | 1.000 - | 1.000 - | 1.000 - | 1.000 - | 1.000 - | 1.000 - |
| Male | 1.366^***^ [1.203,1.552] | 1.367^***^ [1.203,1.552] | 1.655^***^ [1.453,1.884] | 1.588^***^ [1.389,1.816] | 1.354^***^ [1.192,1.538] | 1.358^***^ [1.190,1.550] | 1.548^***^ [1.349,1.777] |
| **Assessment centre:** Manchester | 1.003 [0.700,1.437] | 1.002 [0.699,1.436] | 1.000 [0.698,1.433] | 0.990 [0.691,1.421] | 1.009 [0.704,1.447] | 1.000 [0.697,1.434] | 0.989 [0.690,1.420] |
| Oxford | 0.551^*^ [0.348,0.873] | 0.549^*^ [0.347,0.870] | 0.570^*^ [0.360,0.903] | 0.712 [0.447,1.134] | 0.595^*^ [0.375,0.942] | 0.596^*^ [0.376,0.944] | 0.740 [0.464,1.179] |
| Stoke | 0.775 [0.542,1.108] | 0.775 [0.542,1.108] | 0.782 [0.547,1.118] | 0.839 [0.587,1.198] | 0.780 [0.546,1.116] | 0.768 [0.537,1.098] | 0.821 [0.575,1.173] |
| Reading | 0.534^***^ [0.377,0.755] | 0.532^***^ [0.376,0.752] | 0.560^**^ [0.396,0.792] | 0.722 [0.509,1.025] | 0.594^**^ [0.420,0.842] | 0.570^**^ [0.403,0.807] | 0.748 [0.527,1.062] |
| Bury | 0.655^*^ [0.470,0.911] | 0.655^*^ [0.471,0.911] | 0.658^*^ [0.473,0.916] | 0.677^*^ [0.486,0.942] | 0.652^*^ [0.468,0.907] | 0.650^*^ [0.467,0.905] | 0.664^*^ [0.477,0.925] |
| Newcastle | 1.000 - | 1.000 - | 1.000 - | 1.000 - | 1.000 - | 1.000 - | 1.000 - |
| Leeds | 0.615^**^ [0.460,0.823] | 0.615^**^ [0.460,0.824] | 0.621^**^ [0.464,0.831] | 0.672^**^ [0.502,0.900] | 0.641^**^ [0.479,0.858] | 0.625^**^ [0.467,0.837] | 0.677^**^ [0.506,0.907] |
| Bristol | 0.382^***^ [0.272,0.538] | 0.382^***^ [0.271,0.538] | 0.384^***^ [0.273,0.541] | 0.451^***^ [0.319,0.636] | 0.406^***^ [0.288,0.572] | 0.395^***^ [0.281,0.557] | 0.455^***^ [0.322,0.642] |
| Barts | 0.703 [0.467,1.059] | 0.698 [0.463,1.053] | 0.738 [0.490,1.111] | 0.627^*^ [0.413,0.953] | 0.739 [0.489,1.116] | 0.719 [0.476,1.086] | 0.678 [0.446,1.032] |
| Nottingham | 0.497^***^ [0.354,0.698] | 0.497^***^ [0.354,0.698] | 0.498^***^ [0.355,0.700] | 0.562^***^ [0.400,0.791] | 0.516^***^ [0.367,0.724] | 0.506^***^ [0.360,0.710] | 0.563^***^ [0.400,0.791] |
| Sheffield | 0.837 [0.623,1.126] | 0.838 [0.623,1.126] | 0.813 [0.605,1.093] | 0.876 [0.652,1.178] | 0.834 [0.620,1.121] | 0.845 [0.628,1.136] | 0.858 [0.638,1.153] |
| Liverpool | 0.950 [0.717,1.259] | 0.950 [0.717,1.259] | 0.933 [0.704,1.235] | 0.939 [0.708,1.244] | 0.943 [0.711,1.249] | 0.930 [0.702,1.233] | 0.917 [0.691,1.216] |
| Middlesborough | 0.721 [0.507,1.025] | 0.721 [0.507,1.025] | 0.698^*^ [0.491,0.992] | 0.771 [0.542,1.097] | 0.711 [0.500,1.010] | 0.711 [0.500,1.010] | 0.745 [0.524,1.060] |
| Hounslow | 0.749 [0.551,1.018] | 0.745 [0.548,1.013] | 0.791 [0.583,1.074] | 0.803 [0.588,1.097] | 0.791 [0.581,1.077] | 0.780 [0.572,1.062] | 0.835 [0.611,1.141] |
| Croydon | 0.887 [0.662,1.189] | 0.884 [0.660,1.184] | 0.920 [0.688,1.230] | 0.949 [0.707,1.274] | 0.926 [0.691,1.241] | 0.920 [0.686,1.232] | 0.976 [0.726,1.311] |
| Birmingham | 0.768 [0.561,1.051] | 0.769 [0.562,1.053] | 0.769 [0.562,1.053] | 0.760 [0.555,1.041] | 0.773 [0.564,1.060] | 0.756 [0.552,1.036] | 0.752 [0.548,1.031] |
| **Ethnicity:** White British | 1.000 - | 1.000 - | 1.000 - | 1.000 - | 1.000 - | 1.000 - | 1.000 - |
| White Irish | 1.422 [0.997,2.026] | 1.422 [0.998,2.028] | 1.339 [0.939,1.909] | 1.226 [0.859,1.749] | 1.378 [0.967,1.965] | 1.354 [0.949,1.932] | 1.208 [0.845,1.725] |
| White Other | 1.096 [0.758,1.584] | 1.026 [0.673,1.564] | 1.038 [0.682,1.579] | 1.057 [0.688,1.623] | 1.010 [0.663,1.537] | 0.977 [0.640,1.491] | 1.016 [0.664,1.556] |
| Mixed | 1.155 [0.514,2.598] | 1.121 [0.496,2.532] | 1.077 [0.477,2.434] | 0.958 [0.424,2.165] | 1.058 [0.467,2.393] | 1.012 [0.447,2.291] | 0.922 [0.407,2.090] |
| South Asian | 2.424^***^ [1.751,3.356] | 2.222^***^ [1.482,3.331] | 2.126^***^ [1.415,3.192] | 1.891^**^ [1.243,2.876] | 1.940^**^ [1.288,2.923] | 1.973^**^ [1.313,2.964] | 1.750^**^ [1.150,2.662] |
| Black | 3.350^***^ [2.478,4.530] | 3.127^***^ [2.181,4.484] | 2.655^***^ [1.834,3.842] | 2.050^***^ [1.388,3.029] | 2.816^***^ [1.959,4.048] | 2.585^***^ [1.793,3.728] | 1.923^**^ [1.303,2.840] |
| Chinese | 1.224 [0.394,3.799] | 1.120 [0.352,3.566] | 1.093 [0.343,3.482] | 1.116 [0.349,3.566] | 1.150 [0.361,3.659] | 1.167 [0.367,3.712] | 1.231 [0.386,3.929] |
| Other | 2.197^**^ [1.354,3.567] | 2.029^**^ [1.207,3.413] | 1.792^*^ [1.060,3.030] | 1.473 [0.856,2.533] | 1.836^*^ [1.090,3.091] | 1.683^*^ [1.002,2.828] | 1.382 [0.810,2.359] |
| **Country of birth:** UK & Ireland |  | 1.000 - | 1.000 - | 1.000 - | 1.000 - | 1.000 - | 1.000 - |
| Elsewhere |  | 1.106 [0.828,1.478] | 1.049 [0.781,1.409] | 0.988 [0.721,1.355] | 1.125 [0.840,1.508] | 1.110 [0.827,1.488] | 1.017 [0.743,1.392] |
| **Healthcare worker status**: Non-healthcare worker |  |  | 1.000 - | 1.000 - |  |  | 1.000 - |
| Healthcare worker |  |  | 4.683^***^ [3.873,5.663] | 4.799^***^ [3.954,5.823] |  |  | 4.753^***^ [3.915,5.771] |
| Not in employment |  |  | 1.625^***^ [1.366,1.933] | - |  |  | - |
| **Household size:** 1 |  |  |  | 1.000 - |  |  | 1.000 - |
| 2 |  |  |  | 0.934 [0.783,1.114] |  |  | 0.944 [0.792,1.126] |
| 3 |  |  |  | 1.094 [0.881,1.357] |  |  | 1.085 [0.874,1.346] |
| 4+ |  |  |  | 1.267^*^ [1.028,1.561] |  |  | 1.288^*^ [1.046,1.586] |
| **Education level:** College or University degree |  |  |  | 1.000 - |  |  | 1.000 - |
| A levels/AS levels |  |  |  | 1.179 [0.927,1.499] |  |  | 1.119 [0.879,1.425] |
| O levels/GCSEs/CSEs |  |  |  | 1.139 [0.945,1.372] |  |  | 1.047 [0.867,1.263] |
| Other |  |  |  | 1.316^*^ [1.061,1.631] |  |  | 1.189 [0.957,1.477] |
| None of the above |  |  |  | 1.681^***^ [1.378,2.052] |  |  | 1.460^***^ [1.188,1.794] |
| **Socioeconomic deprivation:** Quartile 1 (most advantaged) |  |  |  | 1.000 - |  |  | 1.000 - |
| Quartile 2 |  |  |  | 1.102 [0.893,1.360] |  |  | 1.075 [0.871,1.327] |
| Quartile 3 |  |  |  | 1.248^*^ [1.018,1.531] |  |  | 1.178 [0.961,1.445] |
| Quartile 4 (least advantaged) |  |  |  | 1.545^***^ [1.251,1.907] |  |  | 1.387^**^ [1.122,1.714] |
| **Housing tenure:** Own |  |  |  | 1.000 - |  |  | 1.000 - |
| Rent/Other |  |  |  | 1.357^**^ [1.118,1.648] |  |  | 1.215 [0.999,1.477] |
| **Urban/rural:** Urban |  |  |  | 1.000 - |  |  | 1.000 - |
| Rural |  |  |  | 0.695^**^ [0.543,0.889] |  |  | 0.714^**^ [0.558,0.914] |
| **Employment status:** In paid employment or self-employed |  |  |  | 1.000 - |  |  | 1.000 - |
| Retired |  |  |  | 1.568^***^ [1.257,1.956] |  |  | 1.461^***^ [1.171,1.823] |
| Looking after home and/or family |  |  |  | 1.287 [0.798,2.076] |  |  | 1.227 [0.761,1.978] |
| Unable to work because of sickness or disability |  |  |  | 2.418^***^ [1.772,3.300] |  |  | 1.518^*^ [1.095,2.105] |
| Unemployed |  |  |  | 0.995 [0.577,1.718] |  |  | 0.936 [0.542,1.615] |
| Other |  |  |  | 1.413 [0.782,2.554] |  |  | 1.384 [0.766,2.502] |
| **Manual occupation:**  Non-manual |  |  |  | 1.000 - |  |  | 1.000 - |
| Manual |  |  |  | 1.596^***^ [1.288,1.978] |  |  | 1.603^***^ [1.294,1.986] |
| Not in employment |  |  |  | - |  |  | - |
| **Long-standing illness/disability:** No |  |  |  |  | 1.000 - |  | 1.000 - |
| Yes |  |  |  |  | 1.198^*^ [1.024,1.402] |  | 1.152 [0.983,1.349] |
| **Number of chronic conditions:** 0 |  |  |  |  | 1.000 - |  | 1.000 - |
| 1 |  |  |  |  | 1.051 [0.884,1.248] |  | 1.015 [0.854,1.206] |
| 2 |  |  |  |  | 1.283^*^ [1.051,1.566] |  | 1.171 [0.957,1.433] |
| 3 |  |  |  |  | 1.499^***^ [1.180,1.904] |  | 1.307^*^ [1.025,1.666] |
| 4+ |  |  |  |  | 1.587^**^ [1.184,2.127] |  | 1.297 [0.965,1.742] |
| **Self-reported health:** Excellent |  |  |  |  | 1.000 - |  | 1.000 - |
| Good |  |  |  |  | 1.067 [0.865,1.317] |  | 0.995 [0.806,1.228] |
| Fair |  |  |  |  | 1.441^**^ [1.135,1.829] |  | 1.208 [0.945,1.546] |
| Poor |  |  |  |  | 2.148^***^ [1.575,2.929] |  | 1.596^**^ [1.142,2.230] |
| **Body Mass Index:** Underweight (<18.5) |  |  |  |  |  | 1.914 [0.852,4.303] | 1.816 [0.810,4.067] |
| Normal weight (18.5-24.9) |  |  |  |  |  | 1.000 - | 1.000 - |
| Overweight (25.0-29.9) |  |  |  |  |  | 1.299^**^ [1.097,1.539] | 1.244^*^ [1.049,1.475] |
| Obese (>=30.0) |  |  |  |  |  | 1.860^***^ [1.561,2.217] | 1.536^***^ [1.278,1.847] |
| **Smoking status:** Never |  |  |  |  |  | 1.000 - | 1.000 - |
| Previous |  |  |  |  |  | 1.491^***^ [1.295,1.716] | 1.349^***^ [1.169,1.557] |
| Current |  |  |  |  |  | 1.398^**^ [1.127,1.734] | 1.111 [0.885,1.395] |
| **Alcohol consumption:** Daily or almost daily |  |  |  |  |  | 1.000 - | 1.000 - |
| Three or four times a week |  |  |  |  |  | 0.988 [0.796,1.227] | 0.958 [0.772,1.189] |
| Once or twice a week |  |  |  |  |  | 1.219 [0.994,1.494] | 1.104 [0.898,1.357] |
| One to three times a month |  |  |  |  |  | 1.484^**^ [1.170,1.883] | 1.271 [0.999,1.616] |
| Special occasions only |  |  |  |  |  | 1.424^**^ [1.119,1.814] | 1.107 [0.863,1.419] |
| Never (former drinker) |  |  |  |  |  | 2.081^***^ [1.542,2.808] | 1.461^*^ [1.074,1.987] |
| Never |  |  |  |  |  | 1.753^***^ [1.303,2.358] | 1.293 [0.953,1.754] |
| **Observations** | 392116 | 392116 | 392116 | 392116 | 392116 | 392116 | 392116 |

Table S5: Ethnicity and risk of testing positive in hospital

|  | **Model 1** | **Model 2** | **Model 3** | **Model 4** | **Model 5** | **Model 6** | **Model 7** |
| --- | --- | --- | --- | --- | --- | --- | --- |
| **Tested positive for SARS-CoV-2 in hospital** | **RR**  **[95% CI]** | **RR**  **[95% CI]** | **RR**  **[95% CI]** | **RR**  **[95% CI]** | **RR**  **[95% CI]** | **RR**  **[95% CI]** | **RR**  **[95% CI]** |
| **Age group:** 40-44 | 1.000 - | 1.000 - | 1.000 - | 1.000 - | 1.000 - | 1.000 - | 1.000 - |
| 45-49 | 0.954 [0.711,1.279] | 0.955 [0.712,1.280] | 0.937 [0.699,1.257] | 0.944 [0.704,1.265] | 0.924 [0.689,1.238] | 0.950 [0.708,1.273] | 0.921 [0.687,1.233] |
| 50-54 | 0.692^*^ [0.508,0.944] | 0.694^*^ [0.509,0.947] | 0.682^*^ [0.500,0.930] | 0.694^*^ [0.509,0.946] | 0.643^**^ [0.472,0.876] | 0.674^*^ [0.494,0.920] | 0.647^**^ [0.474,0.882] |
| 55-59 | 0.778 [0.580,1.044] | 0.780 [0.581,1.048] | 0.759 [0.564,1.022] | 0.803 [0.597,1.079] | 0.695^*^ [0.519,0.930] | 0.741^*^ [0.551,0.996] | 0.725^*^ [0.541,0.973] |
| 60-64 | 0.928 [0.707,1.218] | 0.931 [0.709,1.222] | 0.860 [0.648,1.143] | 0.956 [0.711,1.284] | 0.817 [0.623,1.072] | 0.863 [0.654,1.139] | 0.854 [0.632,1.152] |
| 65-69 | 1.450^**^ [1.114,1.887] | 1.454^**^ [1.116,1.895] | 1.271 [0.952,1.697] | 1.434^*^ [1.041,1.975] | 1.243 [0.951,1.624] | 1.325^*^ [1.011,1.737] | 1.258 [0.906,1.745] |
| 70+ | 2.910^**^ [1.510,5.608] | 2.919^**^ [1.514,5.628] | 2.526^**^ [1.297,4.919] | 2.829^**^ [1.435,5.574] | 2.493^**^ [1.290,4.819] | 2.651^**^ [1.371,5.128] | 2.516^**^ [1.270,4.985] |
| **Sex:** Female | 1.000 - | 1.000 - | 1.000 - | 1.000 - | 1.000 - | 1.000 - | 1.000 - |
| Male | 1.438^***^ [1.243,1.664] | 1.438^***^ [1.243,1.664] | 1.675^***^ [1.443,1.944] | 1.617^***^ [1.387,1.884] | 1.418^***^ [1.226,1.640] | 1.415^***^ [1.216,1.646] | 1.556^***^ [1.330,1.822] |
| **Assessment centre:** Manchester | 1.000 [0.674,1.482] | 1.000 [0.675,1.483] | 0.999 [0.674,1.481] | 0.987 [0.665,1.465] | 1.010 [0.681,1.498] | 0.995 [0.670,1.477] | 0.985 [0.663,1.462] |
| Oxford | 0.568^*^ [0.346,0.933] | 0.569^*^ [0.347,0.935] | 0.588^*^ [0.358,0.966] | 0.752 [0.454,1.244] | 0.624 [0.380,1.026] | 0.620 [0.377,1.020] | 0.784 [0.473,1.299] |
| Stoke | 0.784 [0.533,1.154] | 0.784 [0.533,1.154] | 0.790 [0.537,1.162] | 0.853 [0.580,1.255] | 0.792 [0.538,1.165] | 0.777 [0.527,1.144] | 0.835 [0.568,1.229] |
| Reading | 0.317^***^ [0.200,0.501] | 0.317^***^ [0.201,0.502] | 0.330^***^ [0.209,0.523] | 0.441^***^ [0.278,0.701] | 0.358^***^ [0.226,0.568] | 0.341^***^ [0.216,0.540] | 0.458^***^ [0.288,0.728] |
| Bury | 0.551^**^ [0.377,0.806] | 0.551^**^ [0.377,0.806] | 0.554^**^ [0.379,0.809] | 0.571^**^ [0.390,0.836] | 0.549^**^ [0.375,0.803] | 0.547^**^ [0.374,0.800] | 0.560^**^ [0.382,0.820] |
| Newcastle | 1.000 - | 1.000 - | 1.000 - | 1.000 - | 1.000 - | 1.000 - | 1.000 - |
| Leeds | 0.572^***^ [0.414,0.791] | 0.572^***^ [0.414,0.791] | 0.576^***^ [0.417,0.796] | 0.628^**^ [0.454,0.868] | 0.598^**^ [0.433,0.827] | 0.581^**^ [0.420,0.803] | 0.632^**^ [0.457,0.874] |
| Bristol | 0.304^***^ [0.203,0.456] | 0.305^***^ [0.203,0.457] | 0.306^***^ [0.205,0.459] | 0.368^***^ [0.245,0.553] | 0.326^***^ [0.218,0.490] | 0.316^***^ [0.211,0.474] | 0.371^***^ [0.247,0.559] |
| Barts | 0.622 [0.386,1.001] | 0.625 [0.388,1.007] | 0.654 [0.407,1.052] | 0.549^*^ [0.339,0.891] | 0.666 [0.413,1.074] | 0.642 [0.398,1.036] | 0.596^*^ [0.367,0.968] |
| Nottingham | 0.443^***^ [0.302,0.650] | 0.443^***^ [0.302,0.650] | 0.444^***^ [0.303,0.651] | 0.506^***^ [0.344,0.745] | 0.461^***^ [0.314,0.676] | 0.451^***^ [0.307,0.662] | 0.506^***^ [0.344,0.745] |
| Sheffield | 0.750 [0.538,1.045] | 0.749 [0.537,1.045] | 0.732 [0.525,1.020] | 0.795 [0.570,1.109] | 0.746 [0.535,1.040] | 0.756 [0.542,1.055] | 0.778 [0.558,1.085] |
| Liverpool | 0.942 [0.693,1.280] | 0.942 [0.693,1.279] | 0.927 [0.682,1.259] | 0.936 [0.689,1.272] | 0.935 [0.688,1.270] | 0.923 [0.680,1.254] | 0.917 [0.674,1.247] |
| Middlesborough | 0.755 [0.518,1.100] | 0.755 [0.518,1.100] | 0.736 [0.505,1.073] | 0.820 [0.562,1.195] | 0.744 [0.511,1.084] | 0.745 [0.511,1.085] | 0.790 [0.542,1.152] |
| Hounslow | 0.722 [0.514,1.014] | 0.725 [0.516,1.018] | 0.761 [0.543,1.067] | 0.778 [0.550,1.099] | 0.774 [0.550,1.089] | 0.760 [0.540,1.070] | 0.810 [0.572,1.146] |
| Croydon | 0.605^**^ [0.425,0.861] | 0.606^**^ [0.426,0.863] | 0.627^**^ [0.441,0.890] | 0.651^*^ [0.456,0.929] | 0.637^*^ [0.447,0.908] | 0.633^*^ [0.445,0.902] | 0.670^*^ [0.469,0.957] |
| Birmingham | 0.741 [0.524,1.047] | 0.740 [0.524,1.046] | 0.740 [0.524,1.046] | 0.731 [0.517,1.034] | 0.744 [0.525,1.052] | 0.727 [0.514,1.028] | 0.721 [0.509,1.022] |
| **Ethnicity:** White British | 1.000 - | 1.000 - | 1.000 - | 1.000 - | 1.000 - | 1.000 - | 1.000 - |
| White Irish | 1.581^*^ [1.074,2.327] | 1.580^*^ [1.073,2.326] | 1.507^*^ [1.024,2.218] | 1.369 [0.929,2.018] | 1.527^*^ [1.037,2.248] | 1.494^*^ [1.014,2.201] | 1.343 [0.910,1.982] |
| White Other | 0.908 [0.564,1.462] | 0.950 [0.555,1.627] | 0.957 [0.561,1.635] | 0.982 [0.569,1.693] | 0.934 [0.546,1.596] | 0.904 [0.528,1.548] | 0.939 [0.547,1.613] |
| Mixed | 1.364 [0.560,3.318] | 1.391 [0.567,3.414] | 1.341 [0.547,3.291] | 1.179 [0.481,2.893] | 1.306 [0.531,3.213] | 1.249 [0.508,3.072] | 1.128 [0.458,2.778] |
| South Asian | 2.453^***^ [1.682,3.576] | 2.609^***^ [1.621,4.201] | 2.516^***^ [1.560,4.056] | 2.240^**^ [1.373,3.655] | 2.246^**^ [1.387,3.638] | 2.276^***^ [1.414,3.664] | 2.014^**^ [1.236,3.283] |
| Black | 3.443^***^ [2.399,4.942] | 3.615^***^ [2.370,5.515] | 3.162^***^ [2.057,4.861] | 2.437^***^ [1.557,3.816] | 3.221^***^ [2.104,4.930] | 2.975^***^ [1.938,4.567] | 2.276^***^ [1.454,3.562] |
| Chinese | 1.700 [0.548,5.279] | 1.810 [0.564,5.807] | 1.773 [0.553,5.685] | 1.810 [0.562,5.825] | 1.867 [0.582,5.983] | 1.911 [0.596,6.129] | 2.036 [0.633,6.544] |
| Other | 2.368^**^ [1.362,4.116] | 2.502^**^ [1.406,4.454] | 2.268^**^ [1.271,4.048] | 1.892^*^ [1.042,3.436] | 2.240^**^ [1.256,3.994] | 2.044^*^ [1.148,3.640] | 1.742 [0.967,3.141] |
| **Country of birth:** UK & Ireland |  | 1.000 - | 1.000 - | 1.000 - | 1.000 - | 1.000 - | 1.000 - |
| Elsewhere |  | 0.931 [0.662,1.310] | 0.891 [0.631,1.259] | 0.844 [0.584,1.221] | 0.947 [0.670,1.337] | 0.933 [0.661,1.317] | 0.870 [0.602,1.255] |
| **Healthcare worker status**: Non-healthcare worker |  |  | 1.000 - | 1.000 - |  |  | 1.000 - |
| Healthcare worker |  |  | 3.752^***^ [2.989,4.709] | 3.903^***^ [3.100,4.915] |  |  | 3.866^***^ [3.068,4.871] |
| Not in employment |  |  | 1.506^***^ [1.239,1.831] | - |  |  | - |
| **Household size:** 1 |  |  |  | 1.000 - |  |  | 1.000 - |
| 2 |  |  |  | 0.946 [0.776,1.154] |  |  | 0.953 [0.781,1.163] |
| 3 |  |  |  | 1.079 [0.845,1.377] |  |  | 1.067 [0.837,1.362] |
| 4+ |  |  |  | 1.205 [0.946,1.533] |  |  | 1.223 [0.961,1.555] |
| **Education level:** College or University degree |  |  |  | 1.000 - |  |  | 1.000 - |
| A levels/AS levels |  |  |  | 1.248 [0.945,1.648] |  |  | 1.178 [0.891,1.557] |
| O levels/GCSEs/CSEs |  |  |  | 1.183 [0.952,1.471] |  |  | 1.082 [0.869,1.347] |
| Other |  |  |  | 1.352^*^ [1.052,1.738] |  |  | 1.210 [0.940,1.557] |
| None of the above |  |  |  | 1.827^***^ [1.455,2.293] |  |  | 1.575^***^ [1.247,1.990] |
| **Socioeconomic deprivation:** Quartile 1 (most advantaged) |  |  |  | 1.000 - |  |  | 1.000 - |
| Quartile 2 |  |  |  | 1.238 [0.970,1.580] |  |  | 1.204 [0.943,1.538] |
| Quartile 3 |  |  |  | 1.333^*^ [1.048,1.695] |  |  | 1.253 [0.985,1.592] |
| Quartile 4 (least advantaged) |  |  |  | 1.736^***^ [1.359,2.217] |  |  | 1.544^***^ [1.208,1.972] |
| **Housing tenure:** Own |  |  |  | 1.000 - |  |  | 1.000 - |
| Rent/Other |  |  |  | 1.291^*^ [1.034,1.611] |  |  | 1.148 [0.918,1.437] |
| **Urban/rural:** Urban |  |  |  | 1.000 - |  |  | 1.000 - |
| Rural |  |  |  | 0.667^**^ [0.500,0.889] |  |  | 0.684^**^ [0.513,0.913] |
| **Employment status:** In paid employment or self-employed |  |  |  | 1.000 - |  |  | 1.000 - |
| Retired |  |  |  | 1.417^**^ [1.111,1.807] |  |  | 1.310^*^ [1.027,1.670] |
| Looking after home and/or family |  |  |  | 1.203 [0.691,2.096] |  |  | 1.138 [0.654,1.980] |
| Unable to work because of sickness or disability |  |  |  | 2.186^***^ [1.546,3.090] |  |  | 1.341 [0.935,1.922] |
| Unemployed |  |  |  | 0.771 [0.391,1.519] |  |  | 0.720 [0.365,1.418] |
| Other |  |  |  | 1.599 [0.860,2.973] |  |  | 1.558 [0.837,2.899] |
| **Manual occupation:**  Non-manual |  |  |  | 1.000 - |  |  | 1.000 - |
| Manual |  |  |  | 1.509^**^ [1.176,1.937] |  |  | 1.522^***^ [1.186,1.954] |
| Not in employment |  |  |  | - |  |  | - |
| **Long-standing illness/disability:** No |  |  |  |  | 1.000 - |  | 1.000 - |
| Yes |  |  |  |  | 1.209^*^ [1.011,1.445] |  | 1.170 [0.977,1.401] |
| **Number of chronic conditions:** 0 |  |  |  |  | 1.000 - |  | 1.000 - |
| 1 |  |  |  |  | 1.087 [0.891,1.326] |  | 1.048 [0.859,1.279] |
| 2 |  |  |  |  | 1.337^*^ [1.066,1.677] |  | 1.218 [0.968,1.532] |
| 3 |  |  |  |  | 1.561^**^ [1.192,2.046] |  | 1.352^*^ [1.027,1.781] |
| 4+ |  |  |  |  | 1.511^*^ [1.082,2.111] |  | 1.227 [0.876,1.719] |
| **Self-reported health:** Excellent |  |  |  |  | 1.000 - |  | 1.000 - |
| Good |  |  |  |  | 1.193 [0.928,1.535] |  | 1.095 [0.852,1.408] |
| Fair |  |  |  |  | 1.611^***^ [1.215,2.136] |  | 1.312 [0.983,1.752] |
| Poor |  |  |  |  | 2.529^***^ [1.773,3.608] |  | 1.822^**^ [1.246,2.666] |
| **Body Mass Index:** Underweight (<18.5) |  |  |  |  |  | 2.263 [0.928,5.519] | 2.145 [0.881,5.219] |
| Normal weight (18.5-24.9) |  |  |  |  |  | 1.000 - | 1.000 - |
| Overweight (25.0-29.9) |  |  |  |  |  | 1.374^**^ [1.128,1.674] | 1.296^*^ [1.063,1.580] |
| Obese (>=30.0) |  |  |  |  |  | 2.009^***^ [1.639,2.462] | 1.612^***^ [1.302,1.995] |
| **Smoking status:** Never |  |  |  |  |  | 1.000 - | 1.000 - |
| Previous |  |  |  |  |  | 1.567^***^ [1.333,1.842] | 1.404^***^ [1.192,1.655] |
| Current |  |  |  |  |  | 1.464^**^ [1.143,1.876] | 1.142 [0.881,1.479] |
| **Alcohol consumption:** Daily or almost daily |  |  |  |  |  | 1.000 - | 1.000 - |
| Three or four times a week |  |  |  |  |  | 1.008 [0.791,1.286] | 0.977 [0.766,1.245] |
| Once or twice a week |  |  |  |  |  | 1.152 [0.913,1.454] | 1.030 [0.814,1.303] |
| One to three times a month |  |  |  |  |  | 1.434^**^ [1.092,1.883] | 1.217 [0.924,1.603] |
| Special occasions only |  |  |  |  |  | 1.384^*^ [1.050,1.823] | 1.061 [0.800,1.408] |
| Never (former drinker) |  |  |  |  |  | 2.025^***^ [1.441,2.847] | 1.405 [0.989,1.996] |
| Never |  |  |  |  |  | 1.880^***^ [1.352,2.615] | 1.373 [0.979,1.926] |
| **Observations** | 392116 | 392116 | 392116 | 392116 | 392116 | 392116 | 392116 |

Table S6: Ethnicity and risk of being tested

|  | Model 1 | Model 2 | Model 3 | Model 4 | Model 5 | Model 6 | Model 7 |
| --- | --- | --- | --- | --- | --- | --- | --- |
| Tested for SARS-CoV-2 | RR  [95% CI] | RR  [95% CI] | RR  [95% CI] | RR  [95% CI] | RR  [95% CI] | RR  [95% CI] | RR  [95% CI] |
| **Age group:** 40-44 | 1.000 - | 1.000 - | 1.000 - | 1.000 - | 1.000 - | 1.000 - | 1.000 - |
| 45-49 | 0.805^**^ [0.694,0.934] | 0.805^**^ [0.694,0.934] | 0.790^**^ [0.681,0.916] | 0.795^**^ [0.685,0.922] | 0.784^**^ [0.676,0.910] | 0.805^**^ [0.694,0.935] | 0.782^**^ [0.674,0.907] |
| 50-54 | 0.649^***^ [0.558,0.756] | 0.649^***^ [0.557,0.756] | 0.636^***^ [0.547,0.741] | 0.645^***^ [0.554,0.752] | 0.611^***^ [0.525,0.711] | 0.640^***^ [0.549,0.746] | 0.617^***^ [0.529,0.719] |
| 55-59 | 0.615^***^ [0.530,0.715] | 0.615^***^ [0.530,0.714] | 0.596^***^ [0.514,0.692] | 0.632^***^ [0.543,0.736] | 0.561^***^ [0.483,0.650] | 0.601^***^ [0.517,0.698] | 0.594^***^ [0.510,0.691] |
| 60-64 | 0.769^***^ [0.673,0.880] | 0.769^***^ [0.673,0.880] | 0.703^***^ [0.611,0.809] | 0.819^**^ [0.704,0.953] | 0.695^***^ [0.607,0.796] | 0.742^***^ [0.648,0.851] | 0.768^***^ [0.659,0.895] |
| 65-69 | 1.184^*^ [1.039,1.350] | 1.184^*^ [1.038,1.349] | 1.018 [0.879,1.179] | 1.258^**^ [1.061,1.492] | 1.044 [0.914,1.193] | 1.131 [0.990,1.293] | 1.163 [0.978,1.383] |
| 70+ | 1.875^**^ [1.280,2.748] | 1.874^**^ [1.279,2.747] | 1.597^*^ [1.083,2.354] | 1.970^***^ [1.323,2.934] | 1.652^*^ [1.127,2.422] | 1.792^**^ [1.221,2.630] | 1.835^**^ [1.232,2.734] |
| **Sex:** Female | 1.000 - | 1.000 - | 1.000 - | 1.000 - | 1.000 - | 1.000 - | 1.000 - |
| Male | 1.142^***^ [1.059,1.232] | 1.142^***^ [1.059,1.232] | 1.342^***^ [1.241,1.451] | 1.274^***^ [1.177,1.380] | 1.128^**^ [1.046,1.217] | 1.139^**^ [1.053,1.233] | 1.247^***^ [1.148,1.354] |
| **Assessment centre:** Manchester | 1.114 [0.894,1.388] | 1.114 [0.894,1.388] | 1.111 [0.891,1.384] | 1.084 [0.869,1.351] | 1.119 [0.898,1.395] | 1.099 [0.881,1.370] | 1.074 [0.861,1.339] |
| Oxford | 0.860 [0.676,1.095] | 0.860 [0.676,1.095] | 0.891 [0.700,1.133] | 1.031 [0.808,1.316] | 0.928 [0.728,1.181] | 0.900 [0.707,1.146] | 1.047 [0.820,1.336] |
| Stoke | 0.790^*^ [0.633,0.986] | 0.790^*^ [0.633,0.986] | 0.795^*^ [0.637,0.993] | 0.838 [0.672,1.047] | 0.794^*^ [0.636,0.991] | 0.782^*^ [0.626,0.976] | 0.822 [0.658,1.025] |
| Reading | 0.633^***^ [0.516,0.777] | 0.633^***^ [0.515,0.777] | 0.660^***^ [0.538,0.811] | 0.795^*^ [0.646,0.980] | 0.703^***^ [0.572,0.864] | 0.661^***^ [0.538,0.812] | 0.815 [0.662,1.003] |
| Bury | 0.782^*^ [0.643,0.951] | 0.782^*^ [0.643,0.951] | 0.786^*^ [0.646,0.955] | 0.798^*^ [0.656,0.970] | 0.778^*^ [0.640,0.946] | 0.773^*^ [0.636,0.941] | 0.781^*^ [0.642,0.950] |
| Newcastle | 1.000 - | 1.000 - | 1.000 - | 1.000 - | 1.000 - | 1.000 - | 1.000 - |
| Leeds | 0.365^***^ [0.294,0.453] | 0.365^***^ [0.294,0.453] | 0.368^***^ [0.296,0.456] | 0.389^***^ [0.314,0.483] | 0.380^***^ [0.306,0.471] | 0.368^***^ [0.296,0.456] | 0.391^***^ [0.315,0.485] |
| Bristol | 0.630^***^ [0.525,0.756] | 0.629^***^ [0.524,0.756] | 0.633^***^ [0.527,0.759] | 0.711^***^ [0.591,0.855] | 0.668^***^ [0.556,0.802] | 0.641^***^ [0.534,0.770] | 0.711^***^ [0.591,0.855] |
| Barts | 1.037 [0.822,1.307] | 1.036 [0.821,1.307] | 1.086 [0.862,1.368] | 0.917 [0.724,1.163] | 1.093 [0.866,1.380] | 1.031 [0.817,1.301] | 0.970 [0.765,1.229] |
| Nottingham | 0.674^***^ [0.556,0.817] | 0.674^***^ [0.556,0.817] | 0.676^***^ [0.558,0.819] | 0.738^**^ [0.608,0.894] | 0.699^***^ [0.577,0.847] | 0.679^***^ [0.561,0.823] | 0.736^**^ [0.607,0.892] |
| Sheffield | 1.143 [0.965,1.355] | 1.143 [0.965,1.355] | 1.116 [0.942,1.323] | 1.179 [0.995,1.397] | 1.137 [0.959,1.347] | 1.147 [0.968,1.359] | 1.150 [0.971,1.362] |
| Liverpool | 1.016 [0.855,1.207] | 1.016 [0.855,1.207] | 1.002 [0.843,1.190] | 0.999 [0.840,1.187] | 1.005 [0.846,1.194] | 0.997 [0.839,1.185] | 0.979 [0.824,1.163] |
| Middlesborough | 0.991 [0.814,1.207] | 0.991 [0.814,1.207] | 0.963 [0.791,1.173] | 1.036 [0.850,1.262] | 0.977 [0.802,1.189] | 0.979 [0.804,1.192] | 1.005 [0.825,1.224] |
| Hounslow | 0.799^*^ [0.660,0.968] | 0.799^*^ [0.659,0.967] | 0.838 [0.692,1.013] | 0.833 [0.686,1.011] | 0.847 [0.699,1.026] | 0.814^*^ [0.671,0.987] | 0.850 [0.700,1.032] |
| Croydon | 0.879 [0.730,1.059] | 0.879 [0.730,1.058] | 0.907 [0.754,1.092] | 0.917 [0.760,1.105] | 0.919 [0.763,1.106] | 0.893 [0.741,1.075] | 0.926 [0.768,1.116] |
| Birmingham | 0.823 [0.677,1.001] | 0.823 [0.677,1.001] | 0.824 [0.678,1.002] | 0.809^*^ [0.666,0.984] | 0.828 [0.680,1.006] | 0.805^*^ [0.662,0.979] | 0.799^*^ [0.657,0.972] |
| **Ethnicity:** White British | 1.000 - | 1.000 - | 1.000 - | 1.000 - | 1.000 - | 1.000 - | 1.000 - |
| White Irish | 1.263^*^ [1.013,1.575] | 1.263^*^ [1.013,1.575] | 1.197 [0.960,1.493] | 1.099 [0.880,1.372] | 1.223 [0.982,1.525] | 1.215 [0.975,1.516] | 1.086 [0.870,1.356] |
| White Other | 1.007 [0.809,1.255] | 0.997 [0.773,1.286] | 1.008 [0.782,1.299] | 0.997 [0.770,1.290] | 0.981 [0.761,1.264] | 0.958 [0.742,1.237] | 0.967 [0.749,1.249] |
| Mixed | 1.173 [0.738,1.865] | 1.168 [0.732,1.862] | 1.130 [0.710,1.798] | 1.009 [0.633,1.607] | 1.101 [0.690,1.756] | 1.067 [0.669,1.704] | 0.975 [0.612,1.555] |
| South Asian | 1.560^***^ [1.232,1.975] | 1.539^**^ [1.156,2.048] | 1.475^**^ [1.107,1.965] | 1.351^*^ [1.009,1.808] | 1.349^*^ [1.011,1.801] | 1.392^*^ [1.041,1.861] | 1.252 [0.933,1.681] |
| Black | 2.192^***^ [1.772,2.711] | 2.168^***^ [1.692,2.779] | 1.875^***^ [1.455,2.416] | 1.517^**^ [1.166,1.972] | 1.951^***^ [1.519,2.506] | 1.879^***^ [1.460,2.417] | 1.475^**^ [1.134,1.918] |
| Chinese | 0.546 [0.205,1.455] | 0.538 [0.200,1.453] | 0.527 [0.195,1.421] | 0.534 [0.197,1.442] | 0.555 [0.206,1.496] | 0.537 [0.199,1.451] | 0.558 [0.206,1.508] |
| Other | 1.637^**^ [1.182,2.268] | 1.617^**^ [1.132,2.309] | 1.448^*^ [1.011,2.074] | 1.229 [0.852,1.773] | 1.463^*^ [1.023,2.093] | 1.394 [0.976,1.991] | 1.176 [0.819,1.688] |
| **Country of birth:** UK & Ireland |  | 1.000 - | 1.000 - | 1.000 - | 1.000 - | 1.000 - | 1.000 - |
| Elsewhere |  | 1.016 [0.837,1.233] | 0.977 [0.803,1.189] | 0.943 [0.768,1.157] | 1.034 [0.851,1.256] | 1.016 [0.836,1.235] | 0.964 [0.787,1.181] |
| **Healthcare worker status**: Non-healthcare worker |  |  | 1.000 - | 1.000 - |  |  | 1.000 - |
| Healthcare worker |  |  | 3.873^***^ [3.446,4.353] | 3.911^***^ [3.478,4.399] |  |  | 3.890^***^ [3.459,4.376] |
| Not in employment |  |  | 1.573^***^ [1.413,1.751] | - |  |  | - |
| **Household size:** 1 |  |  |  | 1.000 - |  |  | 1.000 - |
| 2 |  |  |  | 0.844^**^ [0.762,0.935] |  |  | 0.859^**^ [0.776,0.952] |
| 3 |  |  |  | 1.016 [0.895,1.153] |  |  | 1.020 [0.899,1.157] |
| 4+ |  |  |  | 1.041 [0.918,1.180] |  |  | 1.071 [0.945,1.214] |
| **Education level:** College or University degree |  |  |  | 1.000 - |  |  | 1.000 - |
| A levels/AS levels |  |  |  | 1.084 [0.943,1.245] |  |  | 1.045 [0.910,1.201] |
| O levels/GCSEs/CSEs |  |  |  | 1.019 [0.914,1.135] |  |  | 0.963 [0.862,1.074] |
| Other |  |  |  | 1.067 [0.937,1.216] |  |  | 0.994 [0.871,1.134] |
| None of the above |  |  |  | 1.431^***^ [1.272,1.609] |  |  | 1.291^***^ [1.145,1.457] |
| **Socioeconomic deprivation:** Quartile 1 (most advantaged) |  |  |  | 1.000 - |  |  | 1.000 - |
| Quartile 2 |  |  |  | 1.076 [0.954,1.212] |  |  | 1.056 [0.936,1.190] |
| Quartile 3 |  |  |  | 1.204^**^ [1.070,1.354] |  |  | 1.148^*^ [1.020,1.292] |
| Quartile 4 (least advantaged) |  |  |  | 1.331^***^ [1.176,1.507] |  |  | 1.212^**^ [1.070,1.374] |
| **Housing tenure:** Own |  |  |  | 1.000 - |  |  | 1.000 - |
| Rent/Other |  |  |  | 1.348^***^ [1.199,1.516] |  |  | 1.207^**^ [1.072,1.358] |
| **Urban/rural:** Urban |  |  |  | 1.000 - |  |  | 1.000 - |
| Rural |  |  |  | 0.788^***^ [0.690,0.899] |  |  | 0.800^***^ [0.701,0.913] |
| **Employment status:** In paid employment or self-employed |  |  |  | 1.000 - |  |  | 1.000 - |
| Retired |  |  |  | 1.349^***^ [1.183,1.537] |  |  | 1.257^***^ [1.103,1.433] |
| Looking after home and/or family |  |  |  | 1.013 [0.751,1.367] |  |  | 0.964 [0.714,1.300] |
| Unable to work because of sickness or disability |  |  |  | 2.630^***^ [2.205,3.137] |  |  | 1.620^***^ [1.337,1.964] |
| Unemployed |  |  |  | 1.273 [0.949,1.707] |  |  | 1.186 [0.885,1.590] |
| Other |  |  |  | 1.652^**^ [1.204,2.268] |  |  | 1.602^**^ [1.167,2.199] |
| **Manual occupation:**  Non-manual |  |  |  | 1.000 - |  |  | 1.000 - |
| Manual |  |  |  | 1.314^***^ [1.145,1.509] |  |  | 1.314^***^ [1.145,1.508] |
| Not in employment |  |  |  | - |  |  | - |
| **Long-standing illness/disability:** No |  |  |  |  | 1.000 - |  | 1.000 - |
| Yes |  |  |  |  | 1.225^***^ [1.116,1.345] |  | 1.176^***^ [1.070,1.293] |
| **Number of chronic conditions:** 0 |  |  |  |  | 1.000 - |  | 1.000 - |
| 1 |  |  |  |  | 1.036 [0.935,1.147] |  | 1.015 [0.916,1.124] |
| 2 |  |  |  |  | 1.179^**^ [1.045,1.330] |  | 1.113 [0.985,1.257] |
| 3 |  |  |  |  | 1.343^***^ [1.158,1.558] |  | 1.227^**^ [1.056,1.424] |
| 4+ |  |  |  |  | 1.626^***^ [1.371,1.930] |  | 1.414^***^ [1.190,1.681] |
| **Self-reported health:** Excellent |  |  |  |  | 1.000 - |  | 1.000 - |
| Good |  |  |  |  | 1.024 [0.907,1.157] |  | 0.998 [0.883,1.128] |
| Fair |  |  |  |  | 1.382^***^ [1.200,1.592] |  | 1.247^**^ [1.078,1.443] |
| Poor |  |  |  |  | 2.099^***^ [1.745,2.524] |  | 1.647^***^ [1.349,2.010] |
| **Body Mass Index:** Underweight (<18.5) |  |  |  |  |  | 1.460 [0.892,2.390] | 1.352 [0.826,2.212] |
| Normal weight (18.5-24.9) |  |  |  |  |  | 1.000 - | 1.000 - |
| Overweight (25.0-29.9) |  |  |  |  |  | 1.124^*^ [1.021,1.237] | 1.084 [0.984,1.193] |
| Obese (>=30.0) |  |  |  |  |  | 1.470^***^ [1.329,1.627] | 1.216^***^ [1.093,1.353] |
| **Smoking status:** Never |  |  |  |  |  | 1.000 - | 1.000 - |
| Previous |  |  |  |  |  | 1.311^***^ [1.205,1.426] | 1.201^***^ [1.103,1.309] |
| Current |  |  |  |  |  | 1.521^***^ [1.347,1.717] | 1.213^**^ [1.069,1.378] |
| **Alcohol consumption:** Daily or almost daily |  |  |  |  |  | 1.000 - | 1.000 - |
| Three or four times a week |  |  |  |  |  | 0.935 [0.827,1.056] | 0.915 [0.810,1.034] |
| Once or twice a week |  |  |  |  |  | 1.024 [0.910,1.152] | 0.945 [0.839,1.064] |
| One to three times a month |  |  |  |  |  | 1.195^*^ [1.038,1.376] | 1.044 [0.906,1.203] |
| Special occasions only |  |  |  |  |  | 1.296^***^ [1.128,1.488] | 1.033 [0.897,1.190] |
| Never (former drinker) |  |  |  |  |  | 1.677^***^ [1.397,2.013] | 1.176 [0.976,1.416] |
| Never |  |  |  |  |  | 1.448^***^ [1.201,1.745] | 1.108 [0.916,1.340] |
| **Observations** | 392116 | 392116 | 392116 | 392116 | 392116 | 392116 | 392116 |

Table S7: Ethnicity (more defined groups) and risk of testing positive

|  | **Model 1** | **Model 2** | **Model 3** | **Model 4** | **Model 5** | **Model 6** | **Model 7** |
| --- | --- | --- | --- | --- | --- | --- | --- |
| **Tested positive for SARS-CoV-2** | **RR**  **[95% CI]** | **RR**  **[95% CI]** | **RR**  **[95% CI]** | **RR**  **[95% CI]** | **RR**  **[95% CI]** | **RR**  **[95% CI]** | **RR**  **[95% CI]** |
| **Age group:** 40-44 | 1.000 - | 1.000 - | 1.000 - | 1.000 - | 1.000 - | 1.000 - | 1.000 - |
| 45-49 | 0.831 [0.652,1.057] | 0.829 [0.651,1.056] | 0.808 [0.634,1.029] | 0.817 [0.642,1.039] | 0.805 [0.632,1.026] | 0.826 [0.648,1.052] | 0.801 [0.629,1.019] |
| 50-54 | 0.617^***^ [0.478,0.796] | 0.614^***^ [0.475,0.793] | 0.599^***^ [0.464,0.773] | 0.617^***^ [0.478,0.796] | 0.573^***^ [0.444,0.740] | 0.599^***^ [0.463,0.773] | 0.581^***^ [0.451,0.750] |
| 55-59 | 0.588^***^ [0.458,0.756] | 0.585^***^ [0.455,0.753] | 0.566^***^ [0.440,0.728] | 0.614^***^ [0.477,0.791] | 0.527^***^ [0.411,0.675] | 0.560^***^ [0.435,0.720] | 0.563^***^ [0.439,0.724] |
| 60-64 | 0.687^**^ [0.547,0.864] | 0.684^**^ [0.544,0.861] | 0.629^***^ [0.496,0.797] | 0.723^*^ [0.563,0.929] | 0.608^***^ [0.483,0.764] | 0.643^***^ [0.509,0.812] | 0.657^**^ [0.510,0.847] |
| 65-69 | 1.221 [0.984,1.515] | 1.215 [0.978,1.509] | 1.050 [0.829,1.330] | 1.232 [0.937,1.619] | 1.050 [0.844,1.308] | 1.123 [0.900,1.400] | 1.100 [0.834,1.452] |
| 70+ | 2.216^**^ [1.228,4.000] | 2.204^**^ [1.221,3.980] | 1.886^*^ [1.037,3.431] | 2.202^*^ [1.194,4.060] | 1.904^*^ [1.051,3.448] | 2.029^*^ [1.120,3.673] | 1.988^*^ [1.073,3.682] |
| **Sex:** Female | 1.000 - | 1.000 - | 1.000 - | 1.000 - | 1.000 - | 1.000 - | 1.000 - |
| Male | 1.366^***^ [1.203,1.551] | 1.366^***^ [1.203,1.552] | 1.657^***^ [1.456,1.887] | 1.590^***^ [1.391,1.818] | 1.353^***^ [1.192,1.537] | 1.360^***^ [1.192,1.551] | 1.552^***^ [1.353,1.781] |
| **Assessment centre:** Manchester | 0.997 [0.695,1.430] | 0.997 [0.695,1.430] | 0.995 [0.694,1.426] | 0.986 [0.687,1.416] | 1.005 [0.701,1.442] | 0.998 [0.696,1.432] | 0.987 [0.687,1.417] |
| Oxford | 0.551^*^ [0.348,0.873] | 0.549^*^ [0.347,0.870] | 0.570^*^ [0.360,0.902] | 0.710 [0.446,1.131] | 0.595^*^ [0.376,0.942] | 0.596^*^ [0.376,0.944] | 0.739 [0.463,1.177] |
| Stoke | 0.775 [0.542,1.108] | 0.775 [0.542,1.108] | 0.782 [0.547,1.118] | 0.839 [0.587,1.198] | 0.780 [0.546,1.116] | 0.768 [0.537,1.098] | 0.821 [0.575,1.173] |
| Reading | 0.534^***^ [0.378,0.756] | 0.533^***^ [0.377,0.753] | 0.560^**^ [0.396,0.792] | 0.721 [0.508,1.023] | 0.595^**^ [0.420,0.842] | 0.570^**^ [0.403,0.807] | 0.746 [0.526,1.060] |
| Bury | 0.654^*^ [0.470,0.910] | 0.654^*^ [0.470,0.910] | 0.658^*^ [0.473,0.916] | 0.677^*^ [0.486,0.942] | 0.652^*^ [0.468,0.907] | 0.651^*^ [0.468,0.905] | 0.665^*^ [0.478,0.926] |
| Newcastle | 1.000 - | 1.000 - | 1.000 - | 1.000 - | 1.000 - | 1.000 - | 1.000 - |
| Leeds | 0.614^**^ [0.459,0.822] | 0.614^**^ [0.459,0.822] | 0.619^**^ [0.463,0.829] | 0.670^**^ [0.501,0.897] | 0.640^**^ [0.479,0.857] | 0.624^**^ [0.467,0.836] | 0.676^**^ [0.505,0.905] |
| Bristol | 0.382^***^ [0.271,0.538] | 0.382^***^ [0.271,0.537] | 0.383^***^ [0.272,0.540] | 0.449^***^ [0.318,0.634] | 0.406^***^ [0.288,0.572] | 0.395^***^ [0.280,0.556] | 0.453^***^ [0.321,0.640] |
| Barts | 0.705 [0.468,1.064] | 0.701 [0.464,1.058] | 0.743 [0.493,1.119] | 0.631^*^ [0.415,0.958] | 0.739 [0.489,1.117] | 0.723 [0.478,1.094] | 0.680 [0.447,1.036] |
| Nottingham | 0.497^***^ [0.354,0.698] | 0.497^***^ [0.354,0.698] | 0.498^***^ [0.355,0.699] | 0.561^***^ [0.399,0.789] | 0.516^***^ [0.367,0.724] | 0.505^***^ [0.360,0.710] | 0.561^***^ [0.399,0.789] |
| Sheffield | 0.836 [0.622,1.124] | 0.837 [0.622,1.125] | 0.812 [0.604,1.091] | 0.875 [0.651,1.176] | 0.833 [0.619,1.120] | 0.844 [0.628,1.135] | 0.857 [0.638,1.152] |
| Liverpool | 0.950 [0.717,1.259] | 0.951 [0.717,1.260] | 0.934 [0.705,1.237] | 0.939 [0.708,1.245] | 0.943 [0.711,1.250] | 0.930 [0.702,1.233] | 0.917 [0.692,1.216] |
| Middlesborough | 0.720 [0.506,1.024] | 0.720 [0.506,1.023] | 0.696^*^ [0.490,0.990] | 0.770 [0.541,1.096] | 0.710 [0.499,1.009] | 0.710 [0.500,1.009] | 0.744 [0.523,1.059] |
| Hounslow | 0.756 [0.557,1.028] | 0.752 [0.553,1.022] | 0.796 [0.587,1.080] | 0.806 [0.591,1.101] | 0.797 [0.586,1.085] | 0.784 [0.575,1.068] | 0.836 [0.611,1.142] |
| Croydon | 0.888 [0.663,1.190] | 0.885 [0.661,1.185] | 0.921 [0.688,1.231] | 0.950 [0.708,1.275] | 0.926 [0.691,1.241] | 0.919 [0.686,1.232] | 0.975 [0.726,1.310] |
| Birmingham | 0.768 [0.560,1.053] | 0.768 [0.560,1.054] | 0.764 [0.557,1.048] | 0.754 [0.549,1.034] | 0.774 [0.564,1.063] | 0.754 [0.550,1.034] | 0.746 [0.543,1.025] |
| **Ethnicity:** White British | 1.000 - | 1.000 - | 1.000 - | 1.000 - | 1.000 - | 1.000 - | 1.000 - |
| White Irish | 1.420 [0.996,2.025] | 1.421 [0.997,2.026] | 1.338 [0.938,1.908] | 1.225 [0.858,1.749] | 1.378 [0.966,1.965] | 1.354 [0.948,1.932] | 1.208 [0.845,1.725] |
| White Other | 1.094 [0.757,1.582] | 1.015 [0.663,1.554] | 1.015 [0.664,1.552] | 1.028 [0.666,1.587] | 1.005 [0.658,1.536] | 0.962 [0.628,1.474] | 0.991 [0.644,1.524] |
| Mixed | 1.154 [0.513,2.596] | 1.115 [0.493,2.520] | 1.065 [0.471,2.409] | 0.946 [0.418,2.139] | 1.056 [0.466,2.390] | 1.005 [0.444,2.277] | 0.911 [0.401,2.067] |
| Indian | 1.977^**^ [1.264,3.091] | 1.794^*^ [1.093,2.946] | 1.686^*^ [1.027,2.765] | 1.576 [0.951,2.611] | 1.627 [0.988,2.679] | 1.693^*^ [1.033,2.773] | 1.524 [0.924,2.515] |
| Pakistani | 3.243^***^ [1.732,6.072] | 2.944^**^ [1.485,5.837] | 2.858^**^ [1.439,5.676] | 2.260^*^ [1.127,4.533] | 2.394^*^ [1.200,4.777] | 2.148^*^ [1.063,4.339] | 1.839 [0.900,3.757] |
| Other South Asian | 3.003^***^ [1.644,5.486] | 2.704^**^ [1.386,5.277] | 2.516^**^ [1.288,4.915] | 2.091^*^ [1.059,4.131] | 2.351^*^ [1.199,4.611] | 2.356^*^ [1.205,4.609] | 1.939 [0.979,3.841] |
| Black Caribbean | 3.512^***^ [2.394,5.151] | 3.301^***^ [2.196,4.963] | 2.983^***^ [1.982,4.490] | 2.336^***^ [1.534,3.558] | 2.882^***^ [1.908,4.352] | 2.808^***^ [1.864,4.230] | 2.178^***^ [1.431,3.316] |
| Black African | 3.111^***^ [1.973,4.906] | 2.816^***^ [1.648,4.810] | 2.168^**^ [1.259,3.732] | 1.624 [0.921,2.862] | 2.680^***^ [1.564,4.590] | 2.217^**^ [1.294,3.797] | 1.528 [0.869,2.688] |
| Black Other | 4.029 [0.567,28.613] | 3.787 [0.536,26.776] | 2.981 [0.417,21.330] | 2.464 [0.345,17.612] | 3.494 [0.498,24.492] | 3.189 [0.451,22.547] | 2.394 [0.336,17.028] |
| Chinese | 1.223 [0.394,3.796] | 1.106 [0.347,3.526] | 1.063 [0.333,3.391] | 1.078 [0.337,3.451] | 1.144 [0.359,3.647] | 1.143 [0.359,3.641] | 1.189 [0.372,3.801] |
| Other | 2.194^**^ [1.352,3.562] | 2.005^**^ [1.188,3.382] | 1.745^*^ [1.029,2.959] | 1.422 [0.823,2.457] | 1.828^*^ [1.082,3.088] | 1.652 [0.980,2.783] | 1.334 [0.778,2.287] |
| **Country of birth:** UK & Ireland |  | 1.000 - | 1.000 - | 1.000 - | 1.000 - | 1.000 - | 1.000 - |
| Elsewhere |  | 1.121 [0.832,1.509] | 1.081 [0.800,1.460] | 1.024 [0.744,1.409] | 1.131 [0.837,1.527] | 1.134 [0.840,1.529] | 1.052 [0.766,1.444] |
| **Healthcare worker status:** Non-healthcare worker |  |  | 1.000 - | 1.000 - |  |  | 1.000 - |
| Healthcare worker |  |  | 4.707^***^ [3.893,5.693] | 4.819^***^ [3.971,5.848] |  |  | 4.770^***^ [3.929,5.792] |
| Not in employment |  |  | 1.624^***^ [1.365,1.932] | - |  |  | - |
| **Household size:** 1 |  |  |  | 1.000 - |  |  | 1.000 - |
| 2 |  |  |  | 0.937 [0.786,1.117] |  |  | 0.947 [0.794,1.129] |
| 3 |  |  |  | 1.099 [0.885,1.364] |  |  | 1.090 [0.878,1.352] |
| 4+ |  |  |  | 1.277^*^ [1.035,1.574] |  |  | 1.298^*^ [1.054,1.600] |
| **Education level:** College or University degree |  |  |  | 1.000 - |  |  | 1.000 - |
| A levels/AS levels |  |  |  | 1.175 [0.924,1.495] |  |  | 1.116 [0.877,1.421] |
| O levels/GCSEs/CSEs |  |  |  | 1.134 [0.941,1.366] |  |  | 1.042 [0.863,1.258] |
| Other |  |  |  | 1.313^*^ [1.058,1.628] |  |  | 1.186 [0.955,1.474] |
| None of the above |  |  |  | 1.670^***^ [1.369,2.038] |  |  | 1.452^***^ [1.182,1.784] |
| **Socioeconomic deprivation:** Quartile 1 (most advantaged) |  |  |  | 1.000 - |  |  | 1.000 - |
| Quartile 2 |  |  |  | 1.102 [0.893,1.359] |  |  | 1.075 [0.871,1.327] |
| Quartile 3 |  |  |  | 1.247^*^ [1.017,1.529] |  |  | 1.178 [0.960,1.444] |
| Quartile 4 (least advantaged) |  |  |  | 1.542^***^ [1.249,1.904] |  |  | 1.386^**^ [1.122,1.713] |
| **Housing tenure:** Own |  |  |  | 1.000 - |  |  | 1.000 - |
| Rent/Other |  |  |  | 1.365^**^ [1.124,1.658] |  |  | 1.222^*^ [1.005,1.487] |
| **Urban/rural:** Urban |  |  |  | 1.000 - |  |  | 1.000 - |
| Rural |  |  |  | 0.695^**^ [0.543,0.889] |  |  | 0.713^**^ [0.557,0.913] |
| **Employment status:** In paid employment or self-employed |  |  |  | 1.000 - |  |  | 1.000 - |
| Retired |  |  |  | 1.571^***^ [1.259,1.961] |  |  | 1.463^***^ [1.172,1.826] |
| Looking after home and/or family |  |  |  | 1.279 [0.791,2.066] |  |  | 1.222 [0.757,1.974] |
| Unable to work because of sickness or disability |  |  |  | 2.408^***^ [1.764,3.288] |  |  | 1.518^*^ [1.095,2.106] |
| Unemployed |  |  |  | 0.997 [0.577,1.721] |  |  | 0.939 [0.544,1.621] |
| Other |  |  |  | 1.422 [0.787,2.571] |  |  | 1.393 [0.770,2.518] |
| **Manual occupation:** Non-manual |  |  |  | 1.000 - |  |  | 1.000 - |
| Manual |  |  |  | 1.597^***^ [1.289,1.978] |  |  | 1.603^***^ [1.294,1.985] |
| Not in employment |  |  |  | - |  |  | - |
| **Long-standing illness/disability:** No |  |  |  |  | 1.000 - |  | 1.000 - |
| Yes |  |  |  |  | 1.198^*^ [1.024,1.402] |  | 1.151 [0.982,1.348] |
| **Number of chronic conditions:** 0 |  |  |  |  | 1.000 - |  | 1.000 - |
| 1 |  |  |  |  | 1.051 [0.884,1.248] |  | 1.014 [0.853,1.205] |
| 2 |  |  |  |  | 1.283^*^ [1.051,1.567] |  | 1.170 [0.956,1.432] |
| 3 |  |  |  |  | 1.498^***^ [1.180,1.903] |  | 1.305^*^ [1.023,1.663] |
| 4+ |  |  |  |  | 1.588^**^ [1.185,2.127] |  | 1.295 [0.964,1.739] |
| **Self-reported health:** Excellent |  |  |  |  | 1.000 - |  | 1.000 - |
| Good |  |  |  |  | 1.068 [0.865,1.317] |  | 0.995 [0.806,1.229] |
| Fair |  |  |  |  | 1.439^**^ [1.134,1.827] |  | 1.206 [0.943,1.543] |
| Poor |  |  |  |  | 2.139^***^ [1.568,2.918] |  | 1.589^**^ [1.137,2.222] |
| **Body Mass Index:** Underweight (<18.5) |  |  |  |  |  | 1.913 [0.852,4.299] | 1.814 [0.811,4.059] |
| Normal weight (18.5-24.9) |  |  |  |  |  | 1.000 - | 1.000 - |
| Overweight (25.0-29.9) |  |  |  |  |  | 1.299^**^ [1.097,1.539] | 1.245^*^ [1.050,1.477] |
| Obese (>=30.0) |  |  |  |  |  | 1.861^***^ [1.561,2.218] | 1.539^***^ [1.280,1.851] |
| **Smoking status:** Never |  |  |  |  |  | 1.000 - | 1.000 - |
| Previous |  |  |  |  |  | 1.486^***^ [1.291,1.710] | 1.345^***^ [1.166,1.552] |
| Current |  |  |  |  |  | 1.388^**^ [1.120,1.722] | 1.104 [0.880,1.385] |
| **Alcohol consumption:** Daily or almost daily |  |  |  |  |  | 1.000 - | 1.000 - |
| Three or four times a week |  |  |  |  |  | 0.988 [0.796,1.226] | 0.957 [0.772,1.188] |
| Once or twice a week |  |  |  |  |  | 1.219 [0.994,1.494] | 1.104 [0.898,1.357] |
| One to three times a month |  |  |  |  |  | 1.484^**^ [1.170,1.882] | 1.270 [0.999,1.615] |
| Special occasions only |  |  |  |  |  | 1.426^**^ [1.120,1.816] | 1.110 [0.866,1.423] |
| Never (former drinker) |  |  |  |  |  | 2.086^***^ [1.546,2.814] | 1.469^*^ [1.080,1.997] |
| Never |  |  |  |  |  | 1.749^***^ [1.297,2.359] | 1.300 [0.956,1.767] |
| **Observations** | 392116 | 392116 | 392116 | 392116 | 392116 | 392116 | 392116 |

Table S8: Ethnicity (more defined groups) and risk of testing positive in hospital

|  | **Model 1** | **Model 2** | **Model 3** | **Model 4** | **Model 5** | **Model 6** | **Model 7** |
| --- | --- | --- | --- | --- | --- | --- | --- |
| **Tested positive for SARS-CoV-2 in hospital** | **RR**  **[95% CI]** | **RR**  **[95% CI]** | **RR**  **[95% CI]** | **RR**  **[95% CI]** | **RR**  **[95% CI]** | **RR**  **[95% CI]** | **RR**  **[95% CI]** |
| **Age group:** 40-44 | 1.000 - | 1.000 - | 1.000 - | 1.000 - | 1.000 - | 1.000 - | 1.000 - |
| 45-49 | 0.953 [0.711,1.277] | 0.954 [0.711,1.278] | 0.934 [0.697,1.252] | 0.941 [0.702,1.261] | 0.923 [0.688,1.237] | 0.947 [0.706,1.269] | 0.917 [0.685,1.229] |
| 50-54 | 0.692^*^ [0.507,0.944] | 0.693^*^ [0.508,0.946] | 0.679^*^ [0.497,0.926] | 0.690^*^ [0.506,0.942] | 0.642^**^ [0.471,0.875] | 0.671^*^ [0.491,0.917] | 0.643^**^ [0.471,0.879] |
| 55-59 | 0.778 [0.580,1.045] | 0.780 [0.580,1.048] | 0.757 [0.562,1.018] | 0.800 [0.595,1.075] | 0.694^*^ [0.518,0.929] | 0.738^*^ [0.549,0.991] | 0.722^*^ [0.538,0.969] |
| 60-64 | 0.930 [0.708,1.220] | 0.931 [0.709,1.223] | 0.860 [0.647,1.143] | 0.955 [0.710,1.284] | 0.818 [0.623,1.073] | 0.860 [0.652,1.136] | 0.852 [0.631,1.151] |
| 65-69 | 1.449^**^ [1.114,1.886] | 1.452^**^ [1.114,1.891] | 1.267 [0.949,1.691] | 1.430^*^ [1.038,1.969] | 1.241 [0.950,1.621] | 1.318^*^ [1.006,1.726] | 1.253 [0.903,1.739] |
| 70+ | 2.912^**^ [1.510,5.616] | 2.917^**^ [1.511,5.630] | 2.521^**^ [1.293,4.916] | 2.822^**^ [1.431,5.566] | 2.492^**^ [1.288,4.821] | 2.637^**^ [1.362,5.106] | 2.506^**^ [1.264,4.971] |
| **Sex:** Female | 1.000 - | 1.000 - | 1.000 - | 1.000 - | 1.000 - | 1.000 - | 1.000 - |
| Male | 1.440^***^ [1.245,1.665] | 1.440^***^ [1.245,1.665] | 1.679^***^ [1.447,1.949] | 1.619^***^ [1.390,1.887] | 1.419^***^ [1.227,1.642] | 1.419^***^ [1.220,1.650] | 1.561^***^ [1.334,1.827] |
| **Assessment centre:** Manchester | 0.990 [0.667,1.470] | 0.990 [0.667,1.470] | 0.989 [0.666,1.467] | 0.978 [0.658,1.453] | 1.002 [0.675,1.488] | 0.990 [0.666,1.470] | 0.979 [0.658,1.454] |
| Oxford | 0.568^*^ [0.346,0.933] | 0.569^*^ [0.346,0.934] | 0.587^*^ [0.357,0.964] | 0.748 [0.452,1.239] | 0.624 [0.379,1.025] | 0.619 [0.376,1.018] | 0.781 [0.471,1.294] |
| Stoke | 0.784 [0.532,1.154] | 0.784 [0.532,1.154] | 0.789 [0.536,1.162] | 0.852 [0.580,1.254] | 0.791 [0.537,1.165] | 0.776 [0.527,1.143] | 0.834 [0.567,1.227] |
| Reading | 0.317^***^ [0.200,0.502] | 0.317^***^ [0.201,0.502] | 0.330^***^ [0.209,0.523] | 0.441^***^ [0.277,0.700] | 0.358^***^ [0.226,0.568] | 0.341^***^ [0.215,0.540] | 0.457^***^ [0.288,0.726] |
| Bury | 0.549^**^ [0.376,0.803] | 0.549^**^ [0.376,0.803] | 0.552^**^ [0.377,0.807] | 0.569^**^ [0.389,0.833] | 0.547^**^ [0.374,0.801] | 0.546^**^ [0.373,0.798] | 0.559^**^ [0.382,0.818] |
| Newcastle | 1.000 - | 1.000 - | 1.000 - | 1.000 - | 1.000 - | 1.000 - | 1.000 - |
| Leeds | 0.569^***^ [0.412,0.787] | 0.569^***^ [0.412,0.787] | 0.573^***^ [0.414,0.791] | 0.624^**^ [0.451,0.862] | 0.595^**^ [0.431,0.823] | 0.578^***^ [0.418,0.800] | 0.628^**^ [0.454,0.869] |
| Bristol | 0.304^***^ [0.203,0.456] | 0.304^***^ [0.203,0.456] | 0.305^***^ [0.204,0.458] | 0.366^***^ [0.243,0.550] | 0.326^***^ [0.217,0.489] | 0.315^***^ [0.210,0.472] | 0.370^***^ [0.246,0.556] |
| Barts | 0.634 [0.393,1.023] | 0.635 [0.393,1.025] | 0.665 [0.413,1.071] | 0.557^*^ [0.343,0.905] | 0.674 [0.417,1.091] | 0.654 [0.404,1.057] | 0.603^*^ [0.371,0.981] |
| Nottingham | 0.442^***^ [0.301,0.648] | 0.442^***^ [0.301,0.648] | 0.442^***^ [0.301,0.649] | 0.504^***^ [0.342,0.742] | 0.460^***^ [0.313,0.675] | 0.450^***^ [0.306,0.661] | 0.504^***^ [0.342,0.742] |
| Sheffield | 0.748 [0.537,1.043] | 0.748 [0.537,1.043] | 0.731 [0.524,1.019] | 0.794 [0.569,1.107] | 0.745 [0.534,1.038] | 0.756 [0.542,1.054] | 0.777 [0.557,1.084] |
| Liverpool | 0.943 [0.694,1.281] | 0.943 [0.694,1.281] | 0.929 [0.684,1.261] | 0.937 [0.689,1.274] | 0.936 [0.689,1.272] | 0.924 [0.680,1.255] | 0.917 [0.675,1.248] |
| Middlesborough | 0.753 [0.517,1.098] | 0.753 [0.517,1.098] | 0.734 [0.504,1.070] | 0.817 [0.560,1.192] | 0.742 [0.510,1.082] | 0.744 [0.510,1.083] | 0.789 [0.541,1.150] |
| Hounslow | 0.731 [0.521,1.025] | 0.732 [0.522,1.027] | 0.767 [0.548,1.073] | 0.782 [0.554,1.104] | 0.780 [0.555,1.096] | 0.763 [0.543,1.074] | 0.810 [0.573,1.145] |
| Croydon | 0.610^**^ [0.429,0.868] | 0.611^**^ [0.430,0.869] | 0.631^*^ [0.445,0.896] | 0.656^*^ [0.460,0.935] | 0.642^*^ [0.451,0.913] | 0.637^*^ [0.447,0.906] | 0.674^*^ [0.472,0.961] |
| Birmingham | 0.732 [0.516,1.037] | 0.731 [0.516,1.036] | 0.728 [0.514,1.032] | 0.719 [0.507,1.019] | 0.737 [0.520,1.045] | 0.718 [0.507,1.018] | 0.711 [0.500,1.009] |
| **Ethnicity:** White British | 1.000 - | 1.000 - | 1.000 - | 1.000 - | 1.000 - | 1.000 - | 1.000 - |
| White Irish | 1.579^*^ [1.073,2.324] | 1.578^*^ [1.072,2.323] | 1.505^*^ [1.023,2.216] | 1.368 [0.928,2.016] | 1.525^*^ [1.036,2.246] | 1.493^*^ [1.013,2.200] | 1.342 [0.910,1.981] |
| White Other | 0.904 [0.562,1.456] | 0.924 [0.537,1.590] | 0.922 [0.537,1.584] | 0.942 [0.543,1.634] | 0.914 [0.533,1.569] | 0.874 [0.508,1.505] | 0.903 [0.523,1.560] |
| Mixed | 1.360 [0.559,3.309] | 1.373 [0.559,3.374] | 1.318 [0.537,3.239] | 1.156 [0.470,2.843] | 1.294 [0.525,3.185] | 1.230 [0.500,3.030] | 1.106 [0.448,2.731] |
| Indian | 2.171^**^ [1.321,3.567] | 2.236^**^ [1.287,3.882] | 2.125^**^ [1.225,3.687] | 1.991^*^ [1.136,3.490] | 2.001^*^ [1.147,3.490] | 2.102^**^ [1.213,3.642] | 1.897^*^ [1.087,3.313] |
| Pakistani | 3.977^***^ [2.047,7.728] | 4.096^***^ [1.958,8.568] | 3.978^***^ [1.898,8.336] | 3.135^**^ [1.482,6.630] | 3.267^**^ [1.550,6.888] | 2.824^**^ [1.331,5.989] | 2.394^*^ [1.116,5.134] |
| Other South Asian | 1.932 [0.801,4.657] | 1.994 [0.764,5.200] | 1.869 [0.714,4.890] | 1.573 [0.596,4.149] | 1.712 [0.653,4.487] | 1.706 [0.653,4.457] | 1.426 [0.540,3.771] |
| Black Caribbean | 3.864^***^ [2.485,6.009] | 3.938^***^ [2.460,6.304] | 3.610^***^ [2.255,5.778] | 2.780^***^ [1.715,4.505] | 3.404^***^ [2.115,5.480] | 3.343^***^ [2.085,5.359] | 2.593^***^ [1.602,4.199] |
| Black African | 2.769^***^ [1.535,4.993] | 2.853^**^ [1.474,5.524] | 2.296^*^ [1.179,4.472] | 1.743 [0.877,3.467] | 2.694^**^ [1.387,5.232] | 2.216^*^ [1.143,4.295] | 1.623 [0.820,3.215] |
| Black Other | 5.781 [0.812,41.142] | 5.891 [0.833,41.644] | 4.817 [0.674,34.414] | 3.910 [0.548,27.927] | 5.355 [0.763,37.610] | 4.967 [0.703,35.079] | 3.807 [0.536,27.029] |
| Chinese | 1.696 [0.546,5.265] | 1.748 [0.545,5.610] | 1.689 [0.527,5.420] | 1.717 [0.533,5.533] | 1.818 [0.567,5.831] | 1.829 [0.570,5.865] | 1.929 [0.599,6.208] |
| Other | 2.357^**^ [1.356,4.097] | 2.421^**^ [1.357,4.319] | 2.166^**^ [1.210,3.876] | 1.792 [0.983,3.267] | 2.185^**^ [1.223,3.904] | 1.962^*^ [1.099,3.501] | 1.650 [0.912,2.984] |
| **Country of birth:** UK & Ireland |  | 1.000 - | 1.000 - | 1.000 - | 1.000 - | 1.000 - | 1.000 - |
| Elsewhere |  | 0.966 [0.686,1.362] | 0.939 [0.664,1.327] | 0.891 [0.618,1.287] | 0.973 [0.688,1.377] | 0.978 [0.693,1.382] | 0.918 [0.637,1.322] |
| **Healthcare worker status:** Non-healthcare worker |  |  | 1.000 - | 1.000 - |  |  | 1.000 - |
| Healthcare worker |  |  | 3.778^***^ [3.009,4.743] | 3.925^***^ [3.117,4.943] |  |  | 3.885^***^ [3.083,4.895] |
| Not in employment |  |  | 1.506^***^ [1.238,1.831] | - |  |  | - |
| **Household size:** 1 |  |  |  | 1.000 - |  |  | 1.000 - |
| 2 |  |  |  | 0.951 [0.779,1.160] |  |  | 0.957 [0.785,1.168] |
| 3 |  |  |  | 1.086 [0.850,1.386] |  |  | 1.073 [0.841,1.369] |
| 4+ |  |  |  | 1.214 [0.952,1.547] |  |  | 1.233 [0.969,1.570] |
| **Education level:** College or University degree |  |  |  | 1.000 - |  |  | 1.000 - |
| A levels/AS levels |  |  |  | 1.243 [0.941,1.642] |  |  | 1.174 [0.888,1.552] |
| O levels/GCSEs/CSEs |  |  |  | 1.176 [0.946,1.462] |  |  | 1.075 [0.863,1.339] |
| Other |  |  |  | 1.349^*^ [1.049,1.735] |  |  | 1.207 [0.937,1.554] |
| None of the above |  |  |  | 1.812^***^ [1.443,2.275] |  |  | 1.564^***^ [1.237,1.976] |
| **Socioeconomic deprivation:** Quartile 1 (most advantaged) |  |  |  | 1.000 - |  |  | 1.000 - |
| Quartile 2 |  |  |  | 1.237 [0.969,1.579] |  |  | 1.204 [0.943,1.537] |
| Quartile 3 |  |  |  | 1.331^*^ [1.046,1.692] |  |  | 1.251 [0.984,1.590] |
| Quartile 4 (least advantaged) |  |  |  | 1.728^***^ [1.353,2.208] |  |  | 1.540^***^ [1.205,1.967] |
| **Housing tenure:** Own |  |  |  | 1.000 - |  |  | 1.000 - |
| Rent/Other |  |  |  | 1.308^*^ [1.047,1.633] |  |  | 1.164 [0.930,1.457] |
| **Urban/rural:** Urban |  |  |  | 1.000 - |  |  | 1.000 - |
| Rural |  |  |  | 0.667^**^ [0.500,0.889] |  |  | 0.684^**^ [0.513,0.912] |
| **Employment status:** In paid employment or self-employed |  |  |  | 1.000 - |  |  | 1.000 - |
| Retired |  |  |  | 1.419^**^ [1.112,1.810] |  |  | 1.311^*^ [1.028,1.673] |
| Looking after home and/or family |  |  |  | 1.194 [0.684,2.083] |  |  | 1.134 [0.650,1.977] |
| Unable to work because of sickness or disability |  |  |  | 2.174^***^ [1.537,3.075] |  |  | 1.339 [0.934,1.920] |
| Unemployed |  |  |  | 0.775 [0.393,1.527] |  |  | 0.726 [0.368,1.430] |
| Other |  |  |  | 1.620 [0.872,3.011] |  |  | 1.577 [0.848,2.934] |
| **Manual occupation:** Non-manual |  |  |  | 1.000 - |  |  | 1.000 - |
| Manual |  |  |  | 1.514^**^ [1.179,1.942] |  |  | 1.526^***^ [1.189,1.957] |
| Not in employment |  |  |  | - |  |  | - |
| **Long-standing illness/disability:** No |  |  |  |  | 1.000 - |  | 1.000 - |
| Yes |  |  |  |  | 1.209^*^ [1.011,1.445] |  | 1.170 [0.977,1.400] |
| **Number of chronic conditions:** 0 |  |  |  |  | 1.000 - |  | 1.000 - |
| 1 |  |  |  |  | 1.087 [0.891,1.326] |  | 1.047 [0.858,1.277] |
| 2 |  |  |  |  | 1.337^*^ [1.066,1.677] |  | 1.216 [0.967,1.531] |
| 3 |  |  |  |  | 1.559^**^ [1.190,2.043] |  | 1.349^*^ [1.024,1.777] |
| 4+ |  |  |  |  | 1.511^*^ [1.082,2.110] |  | 1.226 [0.875,1.717] |
| **Self-reported health:** Excellent |  |  |  |  | 1.000 - |  | 1.000 - |
| Good |  |  |  |  | 1.195 [0.929,1.537] |  | 1.097 [0.853,1.410] |
| Fair |  |  |  |  | 1.609^***^ [1.214,2.133] |  | 1.310 [0.981,1.748] |
| Poor |  |  |  |  | 2.518^***^ [1.764,3.593] |  | 1.816^**^ [1.241,2.657] |
| **Body Mass Index:** Underweight (<18.5) |  |  |  |  |  | 2.265 [0.929,5.524] | 2.149 [0.883,5.230] |
| Normal weight (18.5-24.9) |  |  |  |  |  | 1.000 - | 1.000 - |
| Overweight (25.0-29.9) |  |  |  |  |  | 1.374^**^ [1.128,1.674] | 1.297^*^ [1.064,1.581] |
| Obese (>=30.0) |  |  |  |  |  | 2.007^***^ [1.637,2.460] | 1.612^***^ [1.302,1.996] |
| **Smoking status:** Never |  |  |  |  |  | 1.000 - | 1.000 - |
| Previous |  |  |  |  |  | 1.564^***^ [1.331,1.837] | 1.401^***^ [1.190,1.651] |
| Current |  |  |  |  |  | 1.454^**^ [1.136,1.862] | 1.135 [0.877,1.470] |
| **Alcohol consumption:** Daily or almost daily |  |  |  |  |  | 1.000 - | 1.000 - |
| Three or four times a week |  |  |  |  |  | 1.008 [0.791,1.285] | 0.976 [0.766,1.244] |
| Once or twice a week |  |  |  |  |  | 1.153 [0.914,1.454] | 1.030 [0.815,1.303] |
| One to three times a month |  |  |  |  |  | 1.434^**^ [1.092,1.883] | 1.217 [0.923,1.603] |
| Special occasions only |  |  |  |  |  | 1.389^*^ [1.054,1.830] | 1.066 [0.803,1.414] |
| Never (former drinker) |  |  |  |  |  | 2.037^***^ [1.449,2.862] | 1.414 [0.996,2.009] |
| Never |  |  |  |  |  | 1.871^***^ [1.343,2.607] | 1.375 [0.979,1.930] |
| **Observations** | 392116 | 392116 | 392116 | 392116 | 392116 | 392116 | 392116 |

Table S9: Ethnicity (more defined groups) and risk of being tested

|  | **Model 1** | **Model 2** | **Model 3** | **Model 4** | **Model 5** | **Model 6** | **Model 7** |
| --- | --- | --- | --- | --- | --- | --- | --- |
| **Tested for SARS-CoV-2** | **RR**  **[95% CI]** | **RR**  **[95% CI]** | **RR**  **[95% CI]** | **RR**  **[95% CI]** | **RR**  **[95% CI]** | **RR**  **[95% CI]** | **RR**  **[95% CI]** |
| **Age group:** 40-44 | 1.000 - | 1.000 - | 1.000 - | 1.000 - | 1.000 - | 1.000 - | 1.000 - |
| 45-49 | 0.806^**^ [0.695,0.935] | 0.806^**^ [0.695,0.935] | 0.790^**^ [0.681,0.917] | 0.795^**^ [0.685,0.922] | 0.786^**^ [0.677,0.911] | 0.806^**^ [0.695,0.935] | 0.782^**^ [0.674,0.907] |
| 50-54 | 0.651^***^ [0.559,0.758] | 0.651^***^ [0.559,0.758] | 0.637^***^ [0.547,0.741] | 0.645^***^ [0.554,0.751] | 0.612^***^ [0.526,0.713] | 0.641^***^ [0.550,0.747] | 0.617^***^ [0.529,0.719] |
| 55-59 | 0.617^***^ [0.531,0.716] | 0.617^***^ [0.531,0.716] | 0.597^***^ [0.515,0.693] | 0.632^***^ [0.543,0.736] | 0.562^***^ [0.484,0.652] | 0.602^***^ [0.518,0.699] | 0.594^***^ [0.510,0.691] |
| 60-64 | 0.771^***^ [0.674,0.882] | 0.771^***^ [0.674,0.882] | 0.704^***^ [0.612,0.811] | 0.819^**^ [0.704,0.953] | 0.697^***^ [0.608,0.798] | 0.744^***^ [0.649,0.852] | 0.768^***^ [0.659,0.895] |
| 65-69 | 1.187^*^ [1.041,1.353] | 1.187^*^ [1.041,1.353] | 1.020 [0.881,1.181] | 1.258^**^ [1.061,1.492] | 1.047 [0.916,1.197] | 1.133 [0.991,1.296] | 1.164 [0.978,1.384] |
| 70+ | 1.879^**^ [1.282,2.755] | 1.879^**^ [1.282,2.754] | 1.600^*^ [1.085,2.359] | 1.970^***^ [1.323,2.933] | 1.657^**^ [1.130,2.428] | 1.795^**^ [1.223,2.634] | 1.835^**^ [1.232,2.734] |
| **Sex:** Female | 1.000 - | 1.000 - | 1.000 - | 1.000 - | 1.000 - | 1.000 - | 1.000 - |
| Male | 1.141^***^ [1.058,1.231] | 1.141^***^ [1.058,1.231] | 1.342^***^ [1.241,1.451] | 1.274^***^ [1.177,1.380] | 1.127^**^ [1.044,1.215] | 1.138^**^ [1.052,1.232] | 1.247^***^ [1.148,1.354] |
| **Assessment centre:** Manchester | 1.110 [0.890,1.384] | 1.110 [0.890,1.384] | 1.107 [0.888,1.380] | 1.081 [0.867,1.348] | 1.116 [0.895,1.392] | 1.096 [0.879,1.367] | 1.072 [0.860,1.337] |
| Oxford | 0.861 [0.676,1.095] | 0.860 [0.676,1.095] | 0.890 [0.700,1.133] | 1.030 [0.807,1.315] | 0.928 [0.729,1.182] | 0.901 [0.707,1.146] | 1.046 [0.820,1.336] |
| Stoke | 0.790^*^ [0.633,0.987] | 0.790^*^ [0.633,0.987] | 0.796^*^ [0.637,0.993] | 0.838 [0.672,1.046] | 0.794^*^ [0.636,0.992] | 0.782^*^ [0.627,0.976] | 0.822 [0.658,1.025] |
| Reading | 0.634^***^ [0.516,0.778] | 0.633^***^ [0.516,0.778] | 0.660^***^ [0.538,0.811] | 0.795^*^ [0.646,0.979] | 0.704^***^ [0.573,0.865] | 0.661^***^ [0.538,0.812] | 0.815 [0.661,1.003] |
| Bury | 0.781^*^ [0.643,0.950] | 0.781^*^ [0.643,0.950] | 0.785^*^ [0.646,0.954] | 0.797^*^ [0.656,0.970] | 0.777^*^ [0.639,0.945] | 0.773^**^ [0.636,0.940] | 0.781^*^ [0.642,0.949] |
| Newcastle | 1.000 - | 1.000 - | 1.000 - | 1.000 - | 1.000 - | 1.000 - | 1.000 - |
| Leeds | 0.365^***^ [0.294,0.453] | 0.365^***^ [0.294,0.453] | 0.367^***^ [0.296,0.456] | 0.389^***^ [0.313,0.482] | 0.379^***^ [0.306,0.471] | 0.367^***^ [0.296,0.456] | 0.390^***^ [0.314,0.484] |
| Bristol | 0.630^***^ [0.525,0.756] | 0.630^***^ [0.525,0.756] | 0.633^***^ [0.527,0.759] | 0.710^***^ [0.590,0.854] | 0.668^***^ [0.557,0.803] | 0.641^***^ [0.534,0.770] | 0.711^***^ [0.591,0.855] |
| Barts | 1.035 [0.820,1.306] | 1.034 [0.819,1.306] | 1.087 [0.863,1.370] | 0.920 [0.725,1.166] | 1.089 [0.862,1.376] | 1.030 [0.816,1.301] | 0.971 [0.765,1.231] |
| Nottingham | 0.675^***^ [0.557,0.818] | 0.675^***^ [0.557,0.818] | 0.676^***^ [0.558,0.819] | 0.737^**^ [0.608,0.894] | 0.699^***^ [0.577,0.848] | 0.680^***^ [0.561,0.824] | 0.736^**^ [0.607,0.892] |
| Sheffield | 1.143 [0.964,1.354] | 1.143 [0.964,1.355] | 1.116 [0.941,1.322] | 1.178 [0.994,1.396] | 1.136 [0.959,1.347] | 1.147 [0.967,1.359] | 1.150 [0.970,1.362] |
| Liverpool | 1.016 [0.855,1.207] | 1.016 [0.855,1.207] | 1.002 [0.843,1.191] | 0.999 [0.840,1.187] | 1.005 [0.846,1.194] | 0.997 [0.839,1.185] | 0.979 [0.824,1.163] |
| Middlesborough | 0.991 [0.814,1.206] | 0.991 [0.814,1.206] | 0.963 [0.791,1.172] | 1.035 [0.849,1.261] | 0.976 [0.802,1.188] | 0.979 [0.804,1.192] | 1.004 [0.824,1.224] |
| Hounslow | 0.803^*^ [0.663,0.973] | 0.803^*^ [0.663,0.973] | 0.841 [0.695,1.018] | 0.835 [0.688,1.014] | 0.851 [0.702,1.031] | 0.816^*^ [0.673,0.990] | 0.852 [0.701,1.034] |
| Croydon | 0.880 [0.731,1.060] | 0.880 [0.731,1.060] | 0.909 [0.755,1.093] | 0.918 [0.762,1.106] | 0.920 [0.764,1.107] | 0.893 [0.742,1.075] | 0.926 [0.769,1.117] |
| Birmingham | 0.825 [0.678,1.003] | 0.825 [0.678,1.003] | 0.823 [0.676,1.001] | 0.807^*^ [0.664,0.983] | 0.830 [0.682,1.010] | 0.806^*^ [0.662,0.980] | 0.798^*^ [0.655,0.971] |
| **Ethnicity:** White British | 1.000 - | 1.000 - | 1.000 - | 1.000 - | 1.000 - | 1.000 - | 1.000 - |
| White Irish | 1.263^*^ [1.013,1.575] | 1.263^*^ [1.013,1.575] | 1.197 [0.960,1.493] | 1.099 [0.880,1.371] | 1.223 [0.981,1.525] | 1.215 [0.975,1.516] | 1.086 [0.870,1.356] |
| White Other | 1.007 [0.808,1.255] | 1.000 [0.774,1.293] | 1.003 [0.777,1.295] | 0.990 [0.764,1.284] | 0.988 [0.765,1.275] | 0.959 [0.741,1.241] | 0.963 [0.744,1.247] |
| Mixed | 1.173 [0.738,1.865] | 1.170 [0.733,1.865] | 1.127 [0.708,1.795] | 1.006 [0.631,1.603] | 1.105 [0.692,1.763] | 1.068 [0.669,1.705] | 0.974 [0.610,1.553] |
| Indian | 1.375^*^ [1.002,1.887] | 1.362 [0.955,1.943] | 1.290 [0.905,1.839] | 1.236 [0.864,1.767] | 1.237 [0.866,1.768] | 1.297 [0.908,1.854] | 1.186 [0.828,1.698] |
| Pakistani | 2.068^**^ [1.283,3.333] | 2.050^**^ [1.239,3.392] | 1.988^**^ [1.200,3.294] | 1.676^*^ [1.008,2.788] | 1.671^*^ [1.006,2.774] | 1.610 [0.962,2.694] | 1.434 [0.853,2.411] |
| Other South Asian | 1.670^*^ [1.035,2.695] | 1.654 [0.991,2.761] | 1.558 [0.933,2.602] | 1.336 [0.796,2.243] | 1.436 [0.858,2.402] | 1.471 [0.879,2.462] | 1.242 [0.738,2.089] |
| Black Caribbean | 2.112^***^ [1.589,2.808] | 2.100^***^ [1.558,2.831] | 1.920^***^ [1.422,2.592] | 1.569^**^ [1.157,2.130] | 1.830^***^ [1.354,2.475] | 1.852^***^ [1.372,2.500] | 1.500^**^ [1.105,2.037] |
| Black African | 2.273^***^ [1.667,3.100] | 2.252^***^ [1.571,3.229] | 1.793^**^ [1.246,2.580] | 1.426 [0.981,2.073] | 2.136^***^ [1.487,3.069] | 1.902^***^ [1.320,2.741] | 1.421 [0.976,2.069] |
| Black Other | 2.707 [0.680,10.773] | 2.691 [0.673,10.769] | 2.215 [0.549,8.936] | 1.830 [0.453,7.388] | 2.469 [0.618,9.866] | 2.344 [0.588,9.341] | 1.827 [0.454,7.352] |
| Chinese | 0.546 [0.205,1.454] | 0.541 [0.200,1.460] | 0.524 [0.194,1.415] | 0.529 [0.196,1.432] | 0.560 [0.208,1.512] | 0.539 [0.199,1.456] | 0.555 [0.205,1.503] |
| Other | 1.637^**^ [1.181,2.267] | 1.623^**^ [1.134,2.323] | 1.441^*^ [1.004,2.068] | 1.220 [0.843,1.764] | 1.477^*^ [1.031,2.116] | 1.398 [0.977,2.001] | 1.171 [0.814,1.685] |
| **Country of birth:** UK & Ireland |  | 1.000 - | 1.000 - | 1.000 - | 1.000 - | 1.000 - | 1.000 - |
| Elsewhere |  | 1.011 [0.829,1.233] | 0.982 [0.804,1.201] | 0.951 [0.772,1.171] | 1.022 [0.836,1.249] | 1.015 [0.831,1.239] | 0.969 [0.788,1.191] |
| **Healthcare worker status:** Non-healthcare worker |  |  | 1.000 - | 1.000 - |  |  | 1.000 - |
| Healthcare worker |  |  | 3.878^***^ [3.450,4.359] | 3.916^***^ [3.482,4.404] |  |  | 3.893^***^ [3.461,4.379] |
| Not in employment |  |  | 1.571^***^ [1.411,1.749] | - |  |  | - |
| **Household size:** 1 |  |  |  | 1.000 - |  |  | 1.000 - |
| 2 |  |  |  | 0.844^**^ [0.762,0.935] |  |  | 0.859^**^ [0.776,0.952] |
| 3 |  |  |  | 1.017 [0.896,1.154] |  |  | 1.021 [0.900,1.158] |
| 4+ |  |  |  | 1.041 [0.918,1.181] |  |  | 1.072 [0.945,1.215] |
| **Education level:** College or University degree |  |  |  | 1.000 - |  |  | 1.000 - |
| A levels/AS levels |  |  |  | 1.083 [0.943,1.245] |  |  | 1.045 [0.909,1.201] |
| O levels/GCSEs/CSEs |  |  |  | 1.018 [0.913,1.135] |  |  | 0.962 [0.862,1.074] |
| Other |  |  |  | 1.067 [0.936,1.215] |  |  | 0.994 [0.871,1.134] |
| None of the above |  |  |  | 1.429^***^ [1.270,1.607] |  |  | 1.291^***^ [1.144,1.456] |
| **Socioeconomic deprivation:** Quartile 1 (most advantaged) |  |  |  | 1.000 - |  |  | 1.000 - |
| Quartile 2 |  |  |  | 1.076 [0.954,1.212] |  |  | 1.055 [0.936,1.190] |
| Quartile 3 |  |  |  | 1.203^**^ [1.070,1.353] |  |  | 1.148^*^ [1.020,1.292] |
| Quartile 4 (least advantaged) |  |  |  | 1.330^***^ [1.174,1.506] |  |  | 1.212^**^ [1.069,1.373] |
| **Housing tenure:** Own |  |  |  | 1.000 - |  |  | 1.000 - |
| Rent/Other |  |  |  | 1.350^***^ [1.200,1.518] |  |  | 1.208^**^ [1.073,1.360] |
| **Urban/rural:** Urban |  |  |  | 1.000 - |  |  | 1.000 - |
| Rural |  |  |  | 0.788^***^ [0.690,0.899] |  |  | 0.800^***^ [0.701,0.913] |
| **Employment status:** In paid employment or self-employed |  |  |  | 1.000 - |  |  | 1.000 - |
| Retired |  |  |  | 1.349^***^ [1.183,1.538] |  |  | 1.258^***^ [1.104,1.433] |
| Looking after home and/or family |  |  |  | 1.010 [0.749,1.363] |  |  | 0.962 [0.713,1.298] |
| Unable to work because of sickness or disability |  |  |  | 2.626^***^ [2.201,3.132] |  |  | 1.620^***^ [1.336,1.963] |
| Unemployed |  |  |  | 1.273 [0.948,1.708] |  |  | 1.187 [0.885,1.591] |
| Other |  |  |  | 1.653^**^ [1.204,2.269] |  |  | 1.603^**^ [1.168,2.200] |
| **Manual occupation:** Non-manual |  |  |  | 1.000 - |  |  | 1.000 - |
| Manual |  |  |  | 1.315^***^ [1.146,1.509] |  |  | 1.315^***^ [1.146,1.509] |
| Not in employment |  |  |  | - |  |  | - |
| **Long-standing illness/disability:** No |  |  |  |  | 1.000 - |  | 1.000 - |
| Yes |  |  |  |  | 1.225^***^ [1.116,1.345] |  | 1.176^***^ [1.070,1.293] |
| **Number of chronic conditions:** 0 |  |  |  |  | 1.000 - |  | 1.000 - |
| 1 |  |  |  |  | 1.036 [0.935,1.147] |  | 1.014 [0.916,1.123] |
| 2 |  |  |  |  | 1.179^**^ [1.045,1.330] |  | 1.112 [0.985,1.256] |
| 3 |  |  |  |  | 1.342^***^ [1.157,1.557] |  | 1.226^**^ [1.056,1.424] |
| 4+ |  |  |  |  | 1.627^***^ [1.371,1.930] |  | 1.414^***^ [1.190,1.681] |
| **Self-reported health:** Excellent |  |  |  |  | 1.000 - |  | 1.000 - |
| Good |  |  |  |  | 1.024 [0.907,1.157] |  | 0.998 [0.883,1.128] |
| Fair |  |  |  |  | 1.383^***^ [1.200,1.593] |  | 1.247^**^ [1.078,1.443] |
| Poor |  |  |  |  | 2.097^***^ [1.744,2.522] |  | 1.646^***^ [1.349,2.009] |
| **Body Mass Index:** Underweight (<18.5) |  |  |  |  |  | 1.460 [0.892,2.390] | 1.352 [0.826,2.213] |
| Normal weight (18.5-24.9) |  |  |  |  |  | 1.000 - | 1.000 - |
| Overweight (25.0-29.9) |  |  |  |  |  | 1.123^*^ [1.021,1.236] | 1.084 [0.984,1.193] |
| Obese (>=30.0) |  |  |  |  |  | 1.470^***^ [1.328,1.627] | 1.216^***^ [1.092,1.352] |
| **Smoking status:** Never |  |  |  |  |  | 1.000 - | 1.000 - |
| Previous |  |  |  |  |  | 1.310^***^ [1.204,1.425] | 1.201^***^ [1.102,1.308] |
| Current |  |  |  |  |  | 1.520^***^ [1.346,1.716] | 1.212^**^ [1.068,1.376] |
| **Alcohol consumption:** Daily or almost daily |  |  |  |  |  | 1.000 - | 1.000 - |
| Three or four times a week |  |  |  |  |  | 0.935 [0.827,1.056] | 0.915 [0.810,1.034] |
| Once or twice a week |  |  |  |  |  | 1.024 [0.910,1.152] | 0.945 [0.839,1.064] |
| One to three times a month |  |  |  |  |  | 1.196^*^ [1.038,1.377] | 1.044 [0.906,1.203] |
| Special occasions only |  |  |  |  |  | 1.296^***^ [1.128,1.488] | 1.033 [0.897,1.190] |
| Never (former drinker) |  |  |  |  |  | 1.676^***^ [1.396,2.012] | 1.176 [0.977,1.417] |
| Never |  |  |  |  |  | 1.435^***^ [1.188,1.733] | 1.101 [0.909,1.334] |
| **Observations** | 392116 | 392116 | 392116 | 392116 | 392116 | 392116 | 392116 |

Table S10: Socioeconomic deprivation and risk of testing positive

|  | **Model 1** | **Model 2** | **Model 3** | **Model 4** | **Model 5** | **Model 6** |
| --- | --- | --- | --- | --- | --- | --- |
| **Tested positive for SARS-CoV-2** | **RR**  **[95% CI]** | **RR**  **[95% CI]** | **RR**  **[95% CI]** | **RR**  **[95% CI]** | **RR**  **[95% CI]** | **RR**  **[95% CI]** |
| **Age group:** 40-44 | 1.000 - | 1.000 - | 1.000 - | 1.000 - | 1.000 - | 1.000 - |
| 45-49 | 0.831 [0.653,1.057] | 0.842 [0.661,1.073] | 0.818 [0.643,1.042] | 0.816 [0.641,1.040] | 0.836 [0.656,1.064] | 0.803 [0.631,1.021] |
| 50-54 | 0.613^***^ [0.475,0.789] | 0.632^***^ [0.490,0.816] | 0.618^***^ [0.480,0.797] | 0.589^***^ [0.456,0.759] | 0.613^***^ [0.475,0.791] | 0.583^***^ [0.452,0.751] |
| 55-59 | 0.577^***^ [0.450,0.740] | 0.611^***^ [0.475,0.785] | 0.615^***^ [0.478,0.792] | 0.548^***^ [0.428,0.702] | 0.579^***^ [0.450,0.745] | 0.564^***^ [0.439,0.724] |
| 60-64 | 0.669^***^ [0.534,0.838] | 0.723^**^ [0.575,0.910] | 0.723^*^ [0.563,0.929] | 0.639^***^ [0.508,0.804] | 0.673^***^ [0.533,0.849] | 0.657^**^ [0.510,0.847] |
| 65-69 | 1.196 [0.966,1.480] | 1.285^*^ [1.033,1.597] | 1.234 [0.938,1.621] | 1.109 [0.890,1.382] | 1.176 [0.942,1.467] | 1.102 [0.835,1.454] |
| 70+ | 2.207^**^ [1.225,3.978] | 2.337^**^ [1.295,4.217] | 2.206^*^ [1.197,4.066] | 2.015^*^ [1.113,3.648] | 2.135^*^ [1.180,3.864] | 1.991^*^ [1.075,3.687] |
| **Sex:** Female | 1.000 - | 1.000 - | 1.000 - | 1.000 - | 1.000 - | 1.000 - |
| Male | 1.368^***^ [1.205,1.553] | 1.362^***^ [1.199,1.547] | 1.588^***^ [1.389,1.816] | 1.352^***^ [1.190,1.535] | 1.355^***^ [1.188,1.546] | 1.548^***^ [1.349,1.777] |
| **Assessment centre**: Manchester | 1.082 [0.756,1.550] | 0.988 [0.688,1.416] | 0.990 [0.691,1.421] | 0.998 [0.696,1.432] | 0.990 [0.690,1.421] | 0.989 [0.690,1.420] |
| Oxford | 0.626^*^ [0.396,0.991] | 0.603^*^ [0.381,0.955] | 0.712 [0.447,1.134] | 0.639 [0.403,1.012] | 0.643 [0.406,1.019] | 0.740 [0.464,1.179] |
| Stoke | 0.864 [0.604,1.235] | 0.853 [0.597,1.219] | 0.839 [0.587,1.198] | 0.842 [0.589,1.203] | 0.830 [0.581,1.187] | 0.821 [0.575,1.173] |
| Reading | 0.673^*^ [0.475,0.955] | 0.636^*^ [0.449,0.901] | 0.722 [0.509,1.025] | 0.680^*^ [0.479,0.965] | 0.655^*^ [0.462,0.928] | 0.748 [0.527,1.062] |
| Bury | 0.726 [0.522,1.009] | 0.693^*^ [0.498,0.964] | 0.677^*^ [0.486,0.942] | 0.682^*^ [0.490,0.949] | 0.682^*^ [0.490,0.948] | 0.664^*^ [0.477,0.925] |
| Newcastle | 1.000 - | 1.000 - | 1.000 - | 1.000 - | 1.000 - | 1.000 - |
| Leeds | 0.685^*^ [0.511,0.918] | 0.657^**^ [0.491,0.879] | 0.672^**^ [0.502,0.900] | 0.673^**^ [0.503,0.901] | 0.657^**^ [0.491,0.880] | 0.677^**^ [0.506,0.907] |
| Bristol | 0.441^***^ [0.313,0.622] | 0.428^***^ [0.304,0.604] | 0.451^***^ [0.319,0.636] | 0.444^***^ [0.315,0.627] | 0.435^***^ [0.308,0.613] | 0.455^***^ [0.322,0.642] |
| Barts | 0.603^*^ [0.399,0.912] | 0.526^**^ [0.347,0.797] | 0.627^*^ [0.413,0.953] | 0.593^*^ [0.391,0.901] | 0.582^*^ [0.383,0.883] | 0.678 [0.446,1.032] |
| Nottingham | 0.560^***^ [0.399,0.787] | 0.539^***^ [0.383,0.757] | 0.562^***^ [0.400,0.791] | 0.548^***^ [0.390,0.770] | 0.539^***^ [0.384,0.758] | 0.563^***^ [0.400,0.791] |
| Sheffield | 0.891 [0.663,1.198] | 0.871 [0.648,1.170] | 0.876 [0.652,1.178] | 0.861 [0.641,1.158] | 0.872 [0.649,1.172] | 0.858 [0.638,1.153] |
| Liverpool | 0.984 [0.742,1.304] | 0.968 [0.730,1.282] | 0.939 [0.708,1.244] | 0.956 [0.721,1.267] | 0.946 [0.714,1.253] | 0.917 [0.691,1.216] |
| Middlesborough | 0.767 [0.539,1.090] | 0.760 [0.535,1.080] | 0.771 [0.542,1.097] | 0.741 [0.521,1.054] | 0.742 [0.522,1.055] | 0.745 [0.524,1.060] |
| Hounslow | 0.845 [0.627,1.140] | 0.705^*^ [0.517,0.960] | 0.803 [0.588,1.097] | 0.758 [0.555,1.034] | 0.749 [0.549,1.022] | 0.835 [0.611,1.141] |
| Croydon | 1.035 [0.777,1.380] | 0.848 [0.633,1.137] | 0.949 [0.707,1.274] | 0.897 [0.669,1.204] | 0.893 [0.665,1.197] | 0.976 [0.726,1.311] |
| Birmingham | 0.863 [0.633,1.177] | 0.752 [0.549,1.031] | 0.760 [0.555,1.041] | 0.760 [0.554,1.042] | 0.746 [0.545,1.023] | 0.752 [0.548,1.031] |
| **Socioeconomic deprivation:** Quartile 1 (most advantaged) | 1.000 - | 1.000 - | 1.000 - | 1.000 - | 1.000 - | 1.000 - |
| Quartile 2 | 1.142 [0.926,1.408] | 1.140 [0.924,1.406] | 1.102 [0.893,1.360] | 1.119 [0.907,1.380] | 1.106 [0.896,1.364] | 1.075 [0.871,1.327] |
| Quartile 3 | 1.396^**^ [1.140,1.709] | 1.375^**^ [1.122,1.684] | 1.248^*^ [1.018,1.531] | 1.302^*^ [1.063,1.596] | 1.288^*^ [1.052,1.578] | 1.178 [0.961,1.445] |
| Quartile 4 (least advantaged) | 2.186^***^ [1.798,2.657] | 2.019^***^ [1.656,2.462] | 1.545^***^ [1.251,1.907] | 1.748^***^ [1.430,2.137] | 1.739^***^ [1.421,2.128] | 1.387^**^ [1.122,1.714] |
| **Ethnicity:** White British |  | 1.000 - | 1.000 - | 1.000 - | 1.000 - | 1.000 - |
| White Irish |  | 1.359 [0.953,1.938] | 1.226 [0.859,1.749] | 1.332 [0.934,1.899] | 1.314 [0.921,1.876] | 1.208 [0.845,1.725] |
| White Other |  | 0.982 [0.643,1.500] | 1.057 [0.688,1.623] | 0.978 [0.642,1.491] | 0.952 [0.623,1.454] | 1.016 [0.664,1.556] |
| Mixed |  | 1.023 [0.453,2.307] | 0.958 [0.424,2.165] | 0.989 [0.438,2.234] | 0.956 [0.423,2.160] | 0.922 [0.407,2.090] |
| South Asian |  | 2.071^***^ [1.378,3.112] | 1.891^**^ [1.243,2.876] | 1.860^**^ [1.234,2.804] | 1.902^**^ [1.263,2.863] | 1.750^**^ [1.150,2.662] |
| Black |  | 2.622^***^ [1.821,3.775] | 2.050^***^ [1.388,3.029] | 2.482^***^ [1.721,3.578] | 2.305^***^ [1.594,3.333] | 1.923^**^ [1.303,2.840] |
| Chinese |  | 1.113 [0.349,3.544] | 1.116 [0.349,3.566] | 1.146 [0.360,3.646] | 1.182 [0.371,3.759] | 1.231 [0.386,3.929] |
| Other |  | 1.815^*^ [1.076,3.063] | 1.473 [0.856,2.533] | 1.702^*^ [1.009,2.872] | 1.582 [0.940,2.662] | 1.382 [0.810,2.359] |
| **Country of birth:** UK & Ireland |  | 1.000 - | 1.000 - | 1.000 - | 1.000 - | 1.000 - |
| Elsewhere |  | 1.069 [0.798,1.431] | 0.988 [0.721,1.355] | 1.096 [0.817,1.469] | 1.082 [0.806,1.453] | 1.017 [0.743,1.392] |
| **Household size:** 1 |  |  | 1.000 - |  |  | 1.000 - |
| 2 |  |  | 0.934 [0.783,1.114] |  |  | 0.944 [0.792,1.126] |
| 3 |  |  | 1.094 [0.881,1.357] |  |  | 1.085 [0.874,1.346] |
| 4+ |  |  | 1.267^*^ [1.028,1.561] |  |  | 1.288^*^ [1.046,1.586] |
| **Education level:** College or University degree |  |  | 1.000 - |  |  | 1.000 - |
| A levels/AS levels |  |  | 1.179 [0.927,1.499] |  |  | 1.119 [0.879,1.425] |
| O levels/GCSEs/CSEs |  |  | 1.139 [0.945,1.372] |  |  | 1.047 [0.867,1.263] |
| Other |  |  | 1.316^*^ [1.061,1.631] |  |  | 1.189 [0.957,1.477] |
| None of the above |  |  | 1.681^***^ [1.378,2.052] |  |  | 1.460^***^ [1.188,1.794] |
| **Housing tenure:** Own |  |  | 1.000 - |  |  | 1.000 - |
| Rent/Other |  |  | 1.357^**^ [1.118,1.648] |  |  | 1.215 [0.999,1.477] |
| **Urban/rural:** Urban |  |  | 1.000 - |  |  | 1.000 - |
| Rural |  |  | 0.695^**^ [0.543,0.889] |  |  | 0.714^**^ [0.558,0.914] |
| **Employment status:** In paid employment or self-employed |  |  | 1.000 - |  |  | 1.000 - |
| Retired |  |  | 1.568^***^ [1.257,1.956] |  |  | 1.461^***^ [1.171,1.823] |
| Looking after home and/or family |  |  | 1.287 [0.798,2.076] |  |  | 1.227 [0.761,1.978] |
| Unable to work because of sickness or disability |  |  | 2.418^***^ [1.772,3.300] |  |  | 1.518^*^ [1.095,2.105] |
| Unemployed |  |  | 0.995 [0.577,1.718] |  |  | 0.936 [0.542,1.615] |
| Other |  |  | 1.413 [0.782,2.554] |  |  | 1.384 [0.766,2.502] |
| **Manual occupation:** Non-manual |  |  | 1.000 - |  |  | 1.000 - |
| Manual |  |  | 1.596^***^ [1.288,1.978] |  |  | 1.603^***^ [1.294,1.986] |
| Not in employment |  |  | - |  |  | - |
| **Healthcare worker status**: Non-healthcare worker |  |  | 1.000 - |  |  | 1.000 - |
| Healthcare worker |  |  | 4.799^***^ [3.954,5.823] |  |  | 4.753^***^ [3.915,5.771] |
| Not in employment |  |  | - |  |  | - |
| **Long-standing illness/disability:** No |  |  |  | 1.000 - |  | 1.000 - |
| Yes |  |  |  | 1.180^*^ [1.009,1.381] |  | 1.152 [0.983,1.349] |
| **Number of chronic conditions:** 0 |  |  |  | 1.000 - |  | 1.000 - |
| 1 |  |  |  | 1.048 [0.882,1.246] |  | 1.015 [0.854,1.206] |
| 2 |  |  |  | 1.270^*^ [1.040,1.550] |  | 1.171 [0.957,1.433] |
| 3 |  |  |  | 1.471^**^ [1.158,1.869] |  | 1.307^*^ [1.025,1.666] |
| 4+ |  |  |  | 1.526^**^ [1.138,2.046] |  | 1.297 [0.965,1.742] |
| **Self-reported health:** Excellent |  |  |  | 1.000 - |  | 1.000 - |
| Good |  |  |  | 1.055 [0.855,1.302] |  | 0.995 [0.806,1.228] |
| Fair |  |  |  | 1.378^**^ [1.085,1.749] |  | 1.208 [0.945,1.546] |
| Poor |  |  |  | 1.962^***^ [1.435,2.682] |  | 1.596^**^ [1.142,2.230] |
| **Body Mass Index:** Underweight (<18.5) |  |  |  |  | 1.873 [0.833,4.210] | 1.816 [0.810,4.067] |
| Normal weight (18.5-24.9) |  |  |  |  | 1.000 - | 1.000 - |
| Overweight (25.0-29.9) |  |  |  |  | 1.293^**^ [1.091,1.531] | 1.244^*^ [1.049,1.475] |
| Obese (>=30.0) |  |  |  |  | 1.795^***^ [1.506,2.139] | 1.536^***^ [1.278,1.847] |
| **Smoking status:** Never |  |  |  |  | 1.000 - | 1.000 - |
| Previous |  |  |  |  | 1.446^***^ [1.256,1.666] | 1.349^***^ [1.169,1.557] |
| Current |  |  |  |  | 1.278^*^ [1.027,1.590] | 1.111 [0.885,1.395] |
| **Alcohol consumption:** Daily or almost daily |  |  |  |  | 1.000 - | 1.000 - |
| Three or four times a week |  |  |  |  | 0.990 [0.798,1.229] | 0.958 [0.772,1.189] |
| Once or twice a week |  |  |  |  | 1.195 [0.975,1.465] | 1.104 [0.898,1.357] |
| One to three times a month |  |  |  |  | 1.439^**^ [1.134,1.827] | 1.271 [0.999,1.616] |
| Special occasions only |  |  |  |  | 1.344^*^ [1.054,1.714] | 1.107 [0.863,1.419] |
| Never (former drinker) |  |  |  |  | 1.921^***^ [1.422,2.594] | 1.461^*^ [1.074,1.987] |
| Never |  |  |  |  | 1.626^**^ [1.206,2.191] | 1.293 [0.953,1.754] |
| **Observations** | 392116 | 392116 | 392116 | 392116 | 392116 | 392116 |

Table S11: Socioeconomic deprivation and risk of testing positive in hospital

|  | **Model 1** | **Model 2** | **Model 3** | **Model 4** | **Model 5** | **Model 6** |
| --- | --- | --- | --- | --- | --- | --- |
| **Tested positive for SARS-CoV-2 in hospital** | **RR**  **[95% CI]** | **RR**  **[95% CI]** | **RR**  **[95% CI]** | **RR**  **[95% CI]** | **RR**  **[95% CI]** | **RR**  **[95% CI]** |
| **Age group:** 40-44 | 1.000 - | 1.000 - | 1.000 - | 1.000 - | 1.000 - | 1.000 - |
| 45-49 | 0.957 [0.714,1.283] | 0.972 [0.725,1.304] | 0.944 [0.704,1.265] | 0.939 [0.700,1.259] | 0.961 [0.716,1.288] | 0.921 [0.687,1.233] |
| 50-54 | 0.690^*^ [0.507,0.940] | 0.717^*^ [0.526,0.979] | 0.694^*^ [0.509,0.946] | 0.663^**^ [0.487,0.904] | 0.691^*^ [0.506,0.943] | 0.647^**^ [0.474,0.882] |
| 55-59 | 0.769 [0.575,1.029] | 0.820 [0.610,1.101] | 0.803 [0.597,1.079] | 0.728^*^ [0.544,0.975] | 0.769 [0.572,1.033] | 0.725^*^ [0.541,0.973] |
| 60-64 | 0.911 [0.697,1.191] | 0.991 [0.754,1.303] | 0.956 [0.711,1.284] | 0.867 [0.660,1.139] | 0.907 [0.687,1.198] | 0.854 [0.632,1.152] |
| 65-69 | 1.429^**^ [1.101,1.855] | 1.548^**^ [1.187,2.019] | 1.434^*^ [1.041,1.975] | 1.324^*^ [1.012,1.731] | 1.393^*^ [1.062,1.826] | 1.258 [0.906,1.745] |
| 70+ | 2.908^**^ [1.511,5.597] | 3.114^***^ [1.615,6.005] | 2.829^**^ [1.435,5.574] | 2.662^**^ [1.377,5.147] | 2.801^**^ [1.448,5.419] | 2.516^**^ [1.270,4.985] |
| **Sex:** Female | 1.000 - | 1.000 - | 1.000 - | 1.000 - | 1.000 - | 1.000 - |
| Male | 1.441^***^ [1.246,1.667] | 1.433^***^ [1.239,1.658] | 1.617^***^ [1.387,1.884] | 1.416^***^ [1.224,1.638] | 1.411^***^ [1.213,1.642] | 1.556^***^ [1.330,1.822] |
| **Assessment centre**: Manchester | 1.085 [0.732,1.607] | 0.987 [0.665,1.465] | 0.987 [0.665,1.465] | 1.000 [0.674,1.485] | 0.986 [0.664,1.464] | 0.985 [0.663,1.462] |
| Oxford | 0.647 [0.394,1.062] | 0.630 [0.384,1.035] | 0.752 [0.454,1.244] | 0.674 [0.410,1.109] | 0.673 [0.409,1.108] | 0.784 [0.473,1.299] |
| Stoke | 0.882 [0.599,1.298] | 0.872 [0.592,1.284] | 0.853 [0.580,1.255] | 0.861 [0.585,1.268] | 0.847 [0.575,1.247] | 0.835 [0.568,1.229] |
| Reading | 0.407^***^ [0.257,0.646] | 0.388^***^ [0.245,0.616] | 0.441^***^ [0.278,0.701] | 0.419^***^ [0.264,0.665] | 0.400^***^ [0.252,0.635] | 0.458^***^ [0.288,0.728] |
| Bury | 0.615^*^ [0.421,0.899] | 0.587^**^ [0.401,0.858] | 0.571^**^ [0.390,0.836] | 0.578^**^ [0.395,0.845] | 0.576^**^ [0.394,0.843] | 0.560^**^ [0.382,0.820] |
| Newcastle | 1.000 - | 1.000 - | 1.000 - | 1.000 - | 1.000 - | 1.000 - |
| Leeds | 0.641^**^ [0.463,0.887] | 0.615^**^ [0.445,0.850] | 0.628^**^ [0.454,0.868] | 0.631^**^ [0.457,0.873] | 0.615^**^ [0.444,0.850] | 0.632^**^ [0.457,0.874] |
| Bristol | 0.355^***^ [0.236,0.533] | 0.347^***^ [0.231,0.520] | 0.368^***^ [0.245,0.553] | 0.362^***^ [0.241,0.543] | 0.352^***^ [0.234,0.528] | 0.371^***^ [0.247,0.559] |
| Barts | 0.518^**^ [0.322,0.833] | 0.458^**^ [0.284,0.739] | 0.549^*^ [0.339,0.891] | 0.523^**^ [0.323,0.847] | 0.507^**^ [0.314,0.820] | 0.596^*^ [0.367,0.968] |
| Nottingham | 0.503^***^ [0.342,0.739] | 0.484^***^ [0.329,0.711] | 0.506^***^ [0.344,0.745] | 0.493^***^ [0.336,0.725] | 0.484^***^ [0.330,0.712] | 0.506^***^ [0.344,0.745] |
| Sheffield | 0.800 [0.574,1.116] | 0.782 [0.561,1.090] | 0.795 [0.570,1.109] | 0.773 [0.555,1.079] | 0.784 [0.562,1.093] | 0.778 [0.558,1.085] |
| Liverpool | 0.980 [0.722,1.331] | 0.960 [0.707,1.304] | 0.936 [0.689,1.272] | 0.949 [0.699,1.289] | 0.940 [0.692,1.277] | 0.917 [0.674,1.247] |
| Middlesborough | 0.805 [0.552,1.173] | 0.799 [0.548,1.163] | 0.820 [0.562,1.195] | 0.778 [0.534,1.133] | 0.780 [0.535,1.136] | 0.790 [0.542,1.152] |
| Hounslow | 0.809 [0.582,1.125] | 0.682^*^ [0.484,0.960] | 0.778 [0.550,1.099] | 0.738 [0.523,1.040] | 0.727 [0.515,1.025] | 0.810 [0.572,1.146] |
| Croydon | 0.703^*^ [0.496,0.998] | 0.580^**^ [0.407,0.826] | 0.651^*^ [0.456,0.929] | 0.616^**^ [0.432,0.879] | 0.613^**^ [0.430,0.874] | 0.670^*^ [0.469,0.957] |
| Birmingham | 0.835 [0.593,1.174] | 0.723 [0.511,1.022] | 0.731 [0.517,1.034] | 0.730 [0.515,1.033] | 0.717 [0.507,1.014] | 0.721 [0.509,1.022] |
| **Socioeconomic deprivation:** Quartile 1 (most advantaged) | 1.000 - | 1.000 - | 1.000 - | 1.000 - | 1.000 - | 1.000 - |
| Quartile 2 | 1.288^*^ [1.009,1.642] | 1.286^*^ [1.008,1.640] | 1.238 [0.970,1.580] | 1.259 [0.987,1.606] | 1.245 [0.976,1.589] | 1.204 [0.943,1.538] |
| Quartile 3 | 1.500^***^ [1.182,1.903] | 1.480^**^ [1.167,1.878] | 1.333^*^ [1.048,1.695] | 1.395^**^ [1.099,1.770] | 1.382^**^ [1.089,1.753] | 1.253 [0.985,1.592] |
| Quartile 4 (least advantaged) | 2.455^***^ [1.956,3.080] | 2.284^***^ [1.814,2.876] | 1.736^***^ [1.359,2.217] | 1.953^***^ [1.547,2.465] | 1.950^***^ [1.544,2.462] | 1.544^***^ [1.208,1.972] |
| **Ethnicity:** White British |  | 1.000 - | 1.000 - | 1.000 - | 1.000 - | 1.000 - |
| White Irish |  | 1.501^*^ [1.019,2.211] | 1.369 [0.929,2.018] | 1.468 [0.996,2.162] | 1.444 [0.979,2.129] | 1.343 [0.910,1.982] |
| White Other |  | 0.907 [0.528,1.555] | 0.982 [0.569,1.693] | 0.902 [0.527,1.543] | 0.879 [0.513,1.506] | 0.939 [0.547,1.613] |
| Mixed |  | 1.254 [0.512,3.071] | 1.179 [0.481,2.893] | 1.210 [0.493,2.970] | 1.170 [0.476,2.873] | 1.128 [0.458,2.778] |
| South Asian |  | 2.415^***^ [1.496,3.898] | 2.240^**^ [1.373,3.655] | 2.145^**^ [1.323,3.478] | 2.185^**^ [1.355,3.525] | 2.014^**^ [1.236,3.283] |
| Black |  | 2.983^***^ [1.951,4.562] | 2.437^***^ [1.557,3.816] | 2.806^***^ [1.830,4.300] | 2.626^***^ [1.709,4.036] | 2.276^***^ [1.454,3.562] |
| Chinese |  | 1.799 [0.561,5.774] | 1.810 [0.562,5.825] | 1.860 [0.580,5.964] | 1.943 [0.606,6.231] | 2.036 [0.633,6.544] |
| Other |  | 2.221^**^ [1.244,3.966] | 1.892^*^ [1.042,3.436] | 2.068^*^ [1.158,3.695] | 1.914^*^ [1.073,3.412] | 1.742 [0.967,3.141] |
| **Country of birth:** UK & Ireland |  | 1.000 - | 1.000 - | 1.000 - | 1.000 - | 1.000 - |
| Elsewhere |  | 0.897 [0.635,1.265] | 0.844 [0.584,1.221] | 0.919 [0.650,1.301] | 0.908 [0.642,1.285] | 0.870 [0.602,1.255] |
| **Household size:** 1 |  |  | 1.000 - |  |  | 1.000 - |
| 2 |  |  | 0.946 [0.776,1.154] |  |  | 0.953 [0.781,1.163] |
| 3 |  |  | 1.079 [0.845,1.377] |  |  | 1.067 [0.837,1.362] |
| 4+ |  |  | 1.205 [0.946,1.533] |  |  | 1.223 [0.961,1.555] |
| **Education level:** College or University degree |  |  | 1.000 - |  |  | 1.000 - |
| A levels/AS levels |  |  | 1.248 [0.945,1.648] |  |  | 1.178 [0.891,1.557] |
| O levels/GCSEs/CSEs |  |  | 1.183 [0.952,1.471] |  |  | 1.082 [0.869,1.347] |
| Other |  |  | 1.352^*^ [1.052,1.738] |  |  | 1.210 [0.940,1.557] |
| None of the above |  |  | 1.827^***^ [1.455,2.293] |  |  | 1.575^***^ [1.247,1.990] |
| **Housing tenure:** Own |  |  | 1.000 - |  |  | 1.000 - |
| Rent/Other |  |  | 1.291^*^ [1.034,1.611] |  |  | 1.148 [0.918,1.437] |
| **Urban/rural:** Urban |  |  | 1.000 - |  |  | 1.000 - |
| Rural |  |  | 0.667^**^ [0.500,0.889] |  |  | 0.684^**^ [0.513,0.913] |
| **Employment status:** In paid employment or self-employed |  |  | 1.000 - |  |  | 1.000 - |
| Retired |  |  | 1.417^**^ [1.111,1.807] |  |  | 1.310^*^ [1.027,1.670] |
| Looking after home and/or family |  |  | 1.203 [0.691,2.096] |  |  | 1.138 [0.654,1.980] |
| Unable to work because of sickness or disability |  |  | 2.186^***^ [1.546,3.090] |  |  | 1.341 [0.935,1.922] |
| Unemployed |  |  | 0.771 [0.391,1.519] |  |  | 0.720 [0.365,1.418] |
| Other |  |  | 1.599 [0.860,2.973] |  |  | 1.558 [0.837,2.899] |
| **Manual occupation:** Non-manual |  |  | 1.000 - |  |  | 1.000 - |
| Manual |  |  | 1.509^**^ [1.176,1.937] |  |  | 1.522^***^ [1.186,1.954] |
| Not in employment |  |  | - |  |  | - |
| **Healthcare worker status**: Non-healthcare worker |  |  | 1.000 - |  |  | 1.000 - |
| Healthcare worker |  |  | 3.903^***^ [3.100,4.915] |  |  | 3.866^***^ [3.068,4.871] |
| Not in employment |  |  | - |  |  | - |
| **Long-standing illness/disability:** No |  |  |  | 1.000 - |  | 1.000 - |
| Yes |  |  |  | 1.189 [0.995,1.421] |  | 1.170 [0.977,1.401] |
| **Number of chronic conditions:** 0 |  |  |  | 1.000 - |  | 1.000 - |
| 1 |  |  |  | 1.085 [0.889,1.323] |  | 1.048 [0.859,1.279] |
| 2 |  |  |  | 1.322^*^ [1.054,1.658] |  | 1.218 [0.968,1.532] |
| 3 |  |  |  | 1.528^**^ [1.166,2.002] |  | 1.352^*^ [1.027,1.781] |
| 4+ |  |  |  | 1.446^*^ [1.034,2.021] |  | 1.227 [0.876,1.719] |
| **Self-reported health:** Excellent |  |  |  | 1.000 - |  | 1.000 - |
| Good |  |  |  | 1.177 [0.915,1.513] |  | 1.095 [0.852,1.408] |
| Fair |  |  |  | 1.529^**^ [1.154,2.028] |  | 1.312 [0.983,1.752] |
| Poor |  |  |  | 2.280^***^ [1.594,3.261] |  | 1.822^**^ [1.246,2.666] |
| **Body Mass Index:** Underweight (<18.5) |  |  |  |  | 2.204 [0.904,5.375] | 2.145 [0.881,5.219] |
| Normal weight (18.5-24.9) |  |  |  |  | 1.000 - | 1.000 - |
| Overweight (25.0-29.9) |  |  |  |  | 1.365^**^ [1.121,1.663] | 1.296^*^ [1.063,1.580] |
| Obese (>=30.0) |  |  |  |  | 1.927^***^ [1.573,2.362] | 1.612^***^ [1.302,1.995] |
| **Smoking status:** Never |  |  |  |  | 1.000 - | 1.000 - |
| Previous |  |  |  |  | 1.513^***^ [1.287,1.778] | 1.404^***^ [1.192,1.655] |
| Current |  |  |  |  | 1.322^*^ [1.029,1.699] | 1.142 [0.881,1.479] |
| **Alcohol consumption:** Daily or almost daily |  |  |  |  | 1.000 - | 1.000 - |
| Three or four times a week |  |  |  |  | 1.009 [0.792,1.286] | 0.977 [0.766,1.245] |
| Once or twice a week |  |  |  |  | 1.125 [0.891,1.419] | 1.030 [0.814,1.303] |
| One to three times a month |  |  |  |  | 1.382^*^ [1.052,1.816] | 1.217 [0.924,1.603] |
| Special occasions only |  |  |  |  | 1.292 [0.979,1.705] | 1.061 [0.800,1.408] |
| Never (former drinker) |  |  |  |  | 1.845^***^ [1.312,2.595] | 1.405 [0.989,1.996] |
| Never |  |  |  |  | 1.725^**^ [1.238,2.402] | 1.373 [0.979,1.926] |
| **Observations** | 392116 | 392116 | 392116 | 392116 | 392116 | 392116 |

Table S12: Socioeconomic deprivation and risk of being tested

|  | **Model 1** | **Model 2** | **Model 3** | **Model 4** | **Model 5** | **Model 6** |
| --- | --- | --- | --- | --- | --- | --- |
| **Tested for SARS-CoV-2** | **RR**  **[95% CI]** | **RR**  **[95% CI]** | **RR**  **[95% CI]** | **RR**  **[95% CI]** | **RR**  **[95% CI]** | **RR**  **[95% CI]** |
| **Age group:** 40-44 | 1.000 - | 1.000 - | 1.000 - | 1.000 - | 1.000 - | 1.000 - |
| 45-49 | 0.810^**^ [0.698,0.940] | 0.815^**^ [0.702,0.946] | 0.795^**^ [0.685,0.922] | 0.792^**^ [0.683,0.919] | 0.812^**^ [0.700,0.942] | 0.782^**^ [0.674,0.907] |
| 50-54 | 0.655^***^ [0.562,0.762] | 0.664^***^ [0.570,0.774] | 0.645^***^ [0.554,0.752] | 0.623^***^ [0.535,0.726] | 0.651^***^ [0.559,0.759] | 0.617^***^ [0.529,0.719] |
| 55-59 | 0.620^***^ [0.534,0.719] | 0.636^***^ [0.548,0.739] | 0.632^***^ [0.543,0.736] | 0.577^***^ [0.498,0.670] | 0.616^***^ [0.530,0.716] | 0.594^***^ [0.510,0.691] |
| 60-64 | 0.777^***^ [0.679,0.888] | 0.804^**^ [0.703,0.920] | 0.819^**^ [0.704,0.953] | 0.722^***^ [0.630,0.827] | 0.768^***^ [0.670,0.881] | 0.768^***^ [0.659,0.895] |
| 65-69 | 1.198^**^ [1.051,1.364] | 1.237^**^ [1.084,1.411] | 1.258^**^ [1.061,1.492] | 1.087 [0.950,1.243] | 1.172^*^ [1.025,1.340] | 1.163 [0.978,1.383] |
| 70+ | 1.911^***^ [1.304,2.800] | 1.962^***^ [1.338,2.875] | 1.970^***^ [1.323,2.934] | 1.721^**^ [1.174,2.523] | 1.859^**^ [1.266,2.728] | 1.835^**^ [1.232,2.734] |
| **Sex:** Female | 1.000 - | 1.000 - | 1.000 - | 1.000 - | 1.000 - | 1.000 - |
| Male | 1.142^***^ [1.059,1.232] | 1.140^***^ [1.057,1.230] | 1.274^***^ [1.177,1.380] | 1.127^**^ [1.045,1.216] | 1.137^**^ [1.051,1.231] | 1.247^***^ [1.148,1.354] |
| **Assessment centre**: Manchester | 1.151 [0.924,1.433] | 1.103 [0.885,1.375] | 1.084 [0.869,1.351] | 1.111 [0.891,1.385] | 1.091 [0.875,1.361] | 1.074 [0.861,1.339] |
| Oxford | 0.934 [0.734,1.189] | 0.923 [0.725,1.175] | 1.031 [0.808,1.316] | 0.974 [0.765,1.240] | 0.950 [0.746,1.210] | 1.047 [0.820,1.336] |
| Stoke | 0.857 [0.687,1.071] | 0.852 [0.683,1.064] | 0.838 [0.672,1.047] | 0.839 [0.672,1.048] | 0.831 [0.665,1.037] | 0.822 [0.658,1.025] |
| Reading | 0.744^**^ [0.605,0.915] | 0.727^**^ [0.591,0.894] | 0.795^*^ [0.646,0.980] | 0.775^*^ [0.630,0.954] | 0.734^**^ [0.597,0.903] | 0.815 [0.662,1.003] |
| Bury | 0.835 [0.687,1.016] | 0.819^*^ [0.673,0.996] | 0.798^*^ [0.656,0.970] | 0.805^*^ [0.662,0.979] | 0.803^*^ [0.660,0.976] | 0.781^*^ [0.642,0.950] |
| Newcastle | 1.000 - | 1.000 - | 1.000 - | 1.000 - | 1.000 - | 1.000 - |
| Leeds | 0.391^***^ [0.315,0.486] | 0.384^***^ [0.310,0.477] | 0.389^***^ [0.314,0.483] | 0.393^***^ [0.317,0.488] | 0.382^***^ [0.308,0.474] | 0.391^***^ [0.315,0.485] |
| Bristol | 0.696^***^ [0.580,0.837] | 0.688^***^ [0.573,0.827] | 0.711^***^ [0.591,0.855] | 0.712^***^ [0.592,0.856] | 0.688^***^ [0.572,0.826] | 0.711^***^ [0.591,0.855] |
| Barts | 0.870 [0.689,1.098] | 0.817 [0.646,1.034] | 0.917 [0.724,1.163] | 0.924 [0.730,1.171] | 0.865 [0.683,1.095] | 0.970 [0.765,1.229] |
| Nottingham | 0.730^**^ [0.602,0.885] | 0.718^***^ [0.592,0.870] | 0.738^**^ [0.608,0.894] | 0.730^**^ [0.602,0.885] | 0.713^***^ [0.588,0.864] | 0.736^**^ [0.607,0.892] |
| Sheffield | 1.192^*^ [1.005,1.413] | 1.179 [0.995,1.398] | 1.179 [0.995,1.397] | 1.164 [0.982,1.380] | 1.175 [0.991,1.393] | 1.150 [0.971,1.362] |
| Liverpool | 1.039 [0.874,1.234] | 1.030 [0.867,1.224] | 0.999 [0.840,1.187] | 1.015 [0.854,1.206] | 1.010 [0.850,1.200] | 0.979 [0.824,1.163] |
| Middlesborough | 1.037 [0.852,1.263] | 1.033 [0.849,1.258] | 1.036 [0.850,1.262] | 1.007 [0.827,1.226] | 1.012 [0.831,1.233] | 1.005 [0.825,1.224] |
| Hounslow | 0.819^*^ [0.678,0.990] | 0.760^**^ [0.627,0.921] | 0.833 [0.686,1.011] | 0.815^*^ [0.672,0.989] | 0.783^*^ [0.645,0.950] | 0.850 [0.700,1.032] |
| Croydon | 0.930 [0.773,1.119] | 0.847 [0.703,1.020] | 0.917 [0.760,1.105] | 0.894 [0.742,1.077] | 0.868 [0.720,1.046] | 0.926 [0.768,1.116] |
| Birmingham | 0.862 [0.711,1.047] | 0.808^*^ [0.665,0.983] | 0.809^*^ [0.666,0.984] | 0.816^*^ [0.671,0.992] | 0.796^*^ [0.654,0.968] | 0.799^*^ [0.657,0.972] |
| **Socioeconomic deprivation:** Quartile 1 (most advantaged) | 1.000 - | 1.000 - | 1.000 - | 1.000 - | 1.000 - | 1.000 - |
| Quartile 2 | 1.108 [0.983,1.249] | 1.107 [0.983,1.248] | 1.076 [0.954,1.212] | 1.087 [0.965,1.226] | 1.083 [0.961,1.220] | 1.056 [0.936,1.190] |
| Quartile 3 | 1.331^***^ [1.184,1.496] | 1.322^***^ [1.176,1.486] | 1.204^**^ [1.070,1.354] | 1.253^***^ [1.115,1.408] | 1.256^***^ [1.117,1.412] | 1.148^*^ [1.020,1.292] |
| Quartile 4 (least advantaged) | 1.817^***^ [1.620,2.038] | 1.750^***^ [1.558,1.965] | 1.331^***^ [1.176,1.507] | 1.510^***^ [1.343,1.697] | 1.543^***^ [1.372,1.737] | 1.212^**^ [1.070,1.374] |
| **Ethnicity:** White British |  | 1.000 - | 1.000 - | 1.000 - | 1.000 - | 1.000 - |
| White Irish |  | 1.219 [0.978,1.521] | 1.099 [0.880,1.372] | 1.194 [0.957,1.488] | 1.188 [0.953,1.482] | 1.086 [0.870,1.356] |
| White Other |  | 0.964 [0.747,1.244] | 0.997 [0.770,1.290] | 0.958 [0.743,1.235] | 0.939 [0.727,1.213] | 0.967 [0.749,1.249] |
| Mixed |  | 1.088 [0.682,1.734] | 1.009 [0.633,1.607] | 1.050 [0.658,1.675] | 1.022 [0.640,1.631] | 0.975 [0.612,1.555] |
| South Asian |  | 1.454^*^ [1.091,1.936] | 1.351^*^ [1.009,1.808] | 1.307 [0.979,1.745] | 1.351^*^ [1.010,1.807] | 1.252 [0.933,1.681] |
| Black |  | 1.892^***^ [1.474,2.430] | 1.517^**^ [1.166,1.972] | 1.786^***^ [1.389,2.296] | 1.723^***^ [1.338,2.218] | 1.475^**^ [1.134,1.918] |
| Chinese |  | 0.535 [0.198,1.443] | 0.534 [0.197,1.442] | 0.552 [0.205,1.489] | 0.540 [0.200,1.457] | 0.558 [0.206,1.508] |
| Other |  | 1.484^*^ [1.038,2.123] | 1.229 [0.852,1.773] | 1.389 [0.971,1.987] | 1.330 [0.931,1.901] | 1.176 [0.819,1.688] |
| **Country of birth:** UK & Ireland |  | 1.000 - | 1.000 - | 1.000 - | 1.000 - | 1.000 - |
| Elsewhere |  | 0.993 [0.817,1.206] | 0.943 [0.768,1.157] | 1.017 [0.836,1.236] | 0.999 [0.822,1.215] | 0.964 [0.787,1.181] |
| **Household size:** 1 |  |  | 1.000 - |  |  | 1.000 - |
| 2 |  |  | 0.844^**^ [0.762,0.935] |  |  | 0.859^**^ [0.776,0.952] |
| 3 |  |  | 1.016 [0.895,1.153] |  |  | 1.020 [0.899,1.157] |
| 4+ |  |  | 1.041 [0.918,1.180] |  |  | 1.071 [0.945,1.214] |
| **Education level:** College or University degree |  |  | 1.000 - |  |  | 1.000 - |
| A levels/AS levels |  |  | 1.084 [0.943,1.245] |  |  | 1.045 [0.910,1.201] |
| O levels/GCSEs/CSEs |  |  | 1.019 [0.914,1.135] |  |  | 0.963 [0.862,1.074] |
| Other |  |  | 1.067 [0.937,1.216] |  |  | 0.994 [0.871,1.134] |
| None of the above |  |  | 1.431^***^ [1.272,1.609] |  |  | 1.291^***^ [1.145,1.457] |
| **Housing tenure:** Own |  |  | 1.000 - |  |  | 1.000 - |
| Rent/Other |  |  | 1.348^***^ [1.199,1.516] |  |  | 1.207^**^ [1.072,1.358] |
| **Urban/rural:** Urban |  |  | 1.000 - |  |  | 1.000 - |
| Rural |  |  | 0.788^***^ [0.690,0.899] |  |  | 0.800^***^ [0.701,0.913] |
| **Employment status:** In paid employment or self-employed |  |  | 1.000 - |  |  | 1.000 - |
| Retired |  |  | 1.349^***^ [1.183,1.537] |  |  | 1.257^***^ [1.103,1.433] |
| Looking after home and/or family |  |  | 1.013 [0.751,1.367] |  |  | 0.964 [0.714,1.300] |
| Unable to work because of sickness or disability |  |  | 2.630^***^ [2.205,3.137] |  |  | 1.620^***^ [1.337,1.964] |
| Unemployed |  |  | 1.273 [0.949,1.707] |  |  | 1.186 [0.885,1.590] |
| Other |  |  | 1.652^**^ [1.204,2.268] |  |  | 1.602^**^ [1.167,2.199] |
| **Manual occupation:** Non-manual |  |  | 1.000 - |  |  | 1.000 - |
| Manual |  |  | 1.314^***^ [1.145,1.509] |  |  | 1.314^***^ [1.145,1.508] |
| Not in employment |  |  | - |  |  | - |
| **Healthcare worker status**: Non-healthcare worker |  |  | 1.000 - |  |  | 1.000 - |
| Healthcare worker |  |  | 3.911^***^ [3.478,4.399] |  |  | 3.890^***^ [3.459,4.376] |
| Not in employment |  |  | - |  |  | - |
| **Long-standing illness/disability:** No |  |  |  | 1.000 - |  | 1.000 - |
| Yes |  |  |  | 1.212^***^ [1.104,1.330] |  | 1.176^***^ [1.070,1.293] |
| **Number of chronic conditions:** 0 |  |  |  | 1.000 - |  | 1.000 - |
| 1 |  |  |  | 1.035 [0.934,1.146] |  | 1.015 [0.916,1.124] |
| 2 |  |  |  | 1.171^*^ [1.038,1.321] |  | 1.113 [0.985,1.257] |
| 3 |  |  |  | 1.325^***^ [1.142,1.537] |  | 1.227^**^ [1.056,1.424] |
| 4+ |  |  |  | 1.580^***^ [1.331,1.875] |  | 1.414^***^ [1.190,1.681] |
| **Self-reported health:** Excellent |  |  |  | 1.000 - |  | 1.000 - |
| Good |  |  |  | 1.017 [0.900,1.148] |  | 0.998 [0.883,1.128] |
| Fair |  |  |  | 1.339^***^ [1.162,1.542] |  | 1.247^**^ [1.078,1.443] |
| Poor |  |  |  | 1.967^***^ [1.634,2.368] |  | 1.647^***^ [1.349,2.010] |
| **Body Mass Index:** Underweight (<18.5) |  |  |  |  | 1.437 [0.878,2.353] | 1.352 [0.826,2.212] |
| Normal weight (18.5-24.9) |  |  |  |  | 1.000 - | 1.000 - |
| Overweight (25.0-29.9) |  |  |  |  | 1.119^*^ [1.017,1.232] | 1.084 [0.984,1.193] |
| Obese (>=30.0) |  |  |  |  | 1.431^***^ [1.293,1.583] | 1.216^***^ [1.093,1.353] |
| **Smoking status:** Never |  |  |  |  | 1.000 - | 1.000 - |
| Previous |  |  |  |  | 1.280^***^ [1.176,1.393] | 1.201^***^ [1.103,1.309] |
| Current |  |  |  |  | 1.418^***^ [1.254,1.603] | 1.213^**^ [1.069,1.378] |
| **Alcohol consumption:** Daily or almost daily |  |  |  |  | 1.000 - | 1.000 - |
| Three or four times a week |  |  |  |  | 0.937 [0.829,1.058] | 0.915 [0.810,1.034] |
| Once or twice a week |  |  |  |  | 1.010 [0.897,1.136] | 0.945 [0.839,1.064] |
| One to three times a month |  |  |  |  | 1.169^*^ [1.015,1.346] | 1.044 [0.906,1.203] |
| Special occasions only |  |  |  |  | 1.241^**^ [1.080,1.426] | 1.033 [0.897,1.190] |
| Never (former drinker) |  |  |  |  | 1.579^***^ [1.315,1.896] | 1.176 [0.976,1.416] |
| Never |  |  |  |  | 1.374^***^ [1.139,1.658] | 1.108 [0.916,1.340] |
| **Observations** | 392116 | 392116 | 392116 | 392116 | 392116 | 392116 |

Table S13: Education level and risk of testing positive

|  | **Model 1** | **Model 2** | **Model 3** | **Model 4** | **Model 5** | **Model 6** |
| --- | --- | --- | --- | --- | --- | --- |
| **Tested positive for SARS-CoV-2** | **RR**  **[95% CI]** | **RR**  **[95% CI]** | **RR**  **[95% CI]** | **RR**  **[95% CI]** | **RR**  **[95% CI]** | **RR**  **[95% CI]** |
| **Age group:** 40-44 | 1.000 - | 1.000 - | 1.000 - | 1.000 - | 1.000 - | 1.000 - |
| 45-49 | 0.799 [0.628,1.017] | 0.814 [0.639,1.036] | 0.818 [0.643,1.042] | 0.793 [0.623,1.011] | 0.814 [0.639,1.037] | 0.803 [0.631,1.021] |
| 50-54 | 0.554^***^ [0.430,0.714] | 0.580^***^ [0.449,0.749] | 0.618^***^ [0.480,0.797] | 0.547^***^ [0.424,0.706] | 0.571^***^ [0.442,0.738] | 0.583^***^ [0.452,0.751] |
| 55-59 | 0.492^***^ [0.383,0.632] | 0.532^***^ [0.413,0.685] | 0.615^***^ [0.478,0.792] | 0.488^***^ [0.380,0.626] | 0.518^***^ [0.402,0.668] | 0.564^***^ [0.439,0.724] |
| 60-64 | 0.524^***^ [0.417,0.658] | 0.584^***^ [0.463,0.736] | 0.723^*^ [0.563,0.929] | 0.535^***^ [0.425,0.675] | 0.568^***^ [0.449,0.719] | 0.657^**^ [0.510,0.847] |
| 65-69 | 0.882 [0.707,1.100] | 0.981 [0.783,1.228] | 1.234 [0.938,1.621] | 0.889 [0.708,1.115] | 0.952 [0.758,1.197] | 1.102 [0.835,1.454] |
| 70+ | 1.607 [0.888,2.908] | 1.767 [0.975,3.200] | 2.206^*^ [1.197,4.066] | 1.604 [0.883,2.915] | 1.718 [0.947,3.119] | 1.991^*^ [1.075,3.687] |
| **Sex:** Female | 1.000 - | 1.000 - | 1.000 - | 1.000 - | 1.000 - | 1.000 - |
| Male | 1.364^***^ [1.200,1.550] | 1.364^***^ [1.200,1.551] | 1.588^***^ [1.389,1.816] | 1.349^***^ [1.187,1.533] | 1.348^***^ [1.180,1.539] | 1.548^***^ [1.349,1.777] |
| **Assessment centre:** Manchester | 1.149 [0.803,1.646] | 1.041 [0.726,1.493] | 0.990 [0.691,1.421] | 1.035 [0.722,1.485] | 1.025 [0.714,1.470] | 0.989 [0.690,1.420] |
| Oxford | 0.623^*^ [0.393,0.988] | 0.602^*^ [0.380,0.955] | 0.712 [0.447,1.134] | 0.635 [0.400,1.007] | 0.628^*^ [0.396,0.996] | 0.740 [0.464,1.179] |
| Stoke | 0.761 [0.532,1.088] | 0.762 [0.533,1.089] | 0.839 [0.587,1.198] | 0.769 [0.538,1.099] | 0.757 [0.530,1.083] | 0.821 [0.575,1.173] |
| Reading | 0.614^**^ [0.434,0.870] | 0.586^**^ [0.414,0.829] | 0.722 [0.509,1.025] | 0.636^*^ [0.449,0.902] | 0.606^**^ [0.428,0.858] | 0.748 [0.527,1.062] |
| Bury | 0.680^*^ [0.489,0.946] | 0.654^*^ [0.470,0.910] | 0.677^*^ [0.486,0.942] | 0.651^*^ [0.468,0.906] | 0.650^*^ [0.467,0.904] | 0.664^*^ [0.477,0.925] |
| Newcastle | 1.000 - | 1.000 - | 1.000 - | 1.000 - | 1.000 - | 1.000 - |
| Leeds | 0.656^**^ [0.490,0.878] | 0.632^**^ [0.472,0.845] | 0.672^**^ [0.502,0.900] | 0.653^**^ [0.488,0.873] | 0.635^**^ [0.475,0.850] | 0.677^**^ [0.506,0.907] |
| Bristol | 0.416^***^ [0.295,0.585] | 0.407^***^ [0.289,0.574] | 0.451^***^ [0.319,0.636] | 0.425^***^ [0.302,0.599] | 0.412^***^ [0.293,0.581] | 0.455^***^ [0.322,0.642] |
| Barts | 0.987 [0.654,1.487] | 0.770 [0.509,1.166] | 0.627^*^ [0.413,0.953] | 0.789 [0.521,1.194] | 0.760 [0.502,1.151] | 0.678 [0.446,1.032] |
| Nottingham | 0.524^***^ [0.373,0.735] | 0.508^***^ [0.362,0.713] | 0.562^***^ [0.400,0.791] | 0.522^***^ [0.372,0.734] | 0.511^***^ [0.364,0.718] | 0.563^***^ [0.400,0.791] |
| Sheffield | 0.878 [0.653,1.181] | 0.861 [0.640,1.157] | 0.876 [0.652,1.178] | 0.851 [0.633,1.144] | 0.859 [0.639,1.154] | 0.858 [0.638,1.153] |
| Liverpool | 0.986 [0.744,1.307] | 0.971 [0.733,1.286] | 0.939 [0.708,1.244] | 0.959 [0.723,1.271] | 0.947 [0.714,1.255] | 0.917 [0.691,1.216] |
| Middlesborough | 0.733 [0.515,1.042] | 0.731 [0.514,1.040] | 0.771 [0.542,1.097] | 0.720 [0.506,1.023] | 0.719 [0.506,1.022] | 0.745 [0.524,1.060] |
| Hounslow | 1.033 [0.766,1.393] | 0.825 [0.605,1.124] | 0.803 [0.588,1.097] | 0.854 [0.626,1.165] | 0.831 [0.609,1.133] | 0.835 [0.611,1.141] |
| Croydon | 1.240 [0.931,1.652] | 0.976 [0.728,1.308] | 0.949 [0.707,1.274] | 0.998 [0.744,1.338] | 0.982 [0.732,1.316] | 0.976 [0.726,1.311] |
| Birmingham | 0.918 [0.673,1.250] | 0.788 [0.575,1.079] | 0.760 [0.555,1.041] | 0.789 [0.576,1.081] | 0.770 [0.562,1.055] | 0.752 [0.548,1.031] |
| **Education level:** College or University degree | 1.000 - | 1.000 - | 1.000 - | 1.000 - | 1.000 - | 1.000 - |
| A levels/AS levels | 1.105 [0.869,1.405] | 1.137 [0.894,1.446] | 1.179 [0.927,1.499] | 1.094 [0.860,1.392] | 1.079 [0.847,1.374] | 1.119 [0.879,1.425] |
| O levels/GCSEs/CSEs | 1.127 [0.938,1.353] | 1.144 [0.953,1.373] | 1.139 [0.945,1.372] | 1.078 [0.897,1.294] | 1.040 [0.865,1.251] | 1.047 [0.867,1.263] |
| Other | 1.636^***^ [1.325,2.021] | 1.575^***^ [1.275,1.945] | 1.316^*^ [1.061,1.631] | 1.465^***^ [1.186,1.809] | 1.404^**^ [1.134,1.737] | 1.189 [0.957,1.477] |
| None of the above | 2.004^***^ [1.663,2.417] | 1.956^***^ [1.625,2.353] | 1.681^***^ [1.378,2.052] | 1.677^***^ [1.387,2.027] | 1.626^***^ [1.340,1.973] | 1.460^***^ [1.188,1.794] |
| **Ethnicity:** White British |  | 1.000 - | 1.000 - | 1.000 - | 1.000 - | 1.000 - |
| White Irish |  | 1.388 [0.973,1.979] | 1.226 [0.859,1.749] | 1.352 [0.948,1.928] | 1.332 [0.933,1.902] | 1.208 [0.845,1.725] |
| White Other |  | 1.073 [0.700,1.645] | 1.057 [0.688,1.623] | 1.045 [0.684,1.598] | 1.011 [0.659,1.549] | 1.016 [0.664,1.556] |
| Mixed |  | 1.096 [0.485,2.475] | 0.958 [0.424,2.165] | 1.046 [0.462,2.366] | 1.008 [0.445,2.281] | 0.922 [0.407,2.090] |
| South Asian |  | 2.149^***^ [1.423,3.245] | 1.891^**^ [1.243,2.876] | 1.923^**^ [1.270,2.912] | 1.977^**^ [1.309,2.986] | 1.750^**^ [1.150,2.662] |
| Black |  | 2.886^***^ [1.993,4.178] | 2.050^***^ [1.388,3.029] | 2.667^***^ [1.841,3.865] | 2.508^***^ [1.729,3.640] | 1.923^**^ [1.303,2.840] |
| Chinese |  | 1.115 [0.349,3.562] | 1.116 [0.349,3.566] | 1.152 [0.361,3.676] | 1.180 [0.370,3.763] | 1.231 [0.386,3.929] |
| Other |  | 1.946^*^ [1.147,3.300] | 1.473 [0.856,2.533] | 1.795^*^ [1.060,3.041] | 1.675 [0.991,2.830] | 1.382 [0.810,2.359] |
| **Country of birth:** UK & Ireland |  | 1.000 - | 1.000 - | 1.000 - | 1.000 - | 1.000 - |
| Elsewhere |  | 1.121 [0.831,1.512] | 0.988 [0.721,1.355] | 1.132 [0.839,1.527] | 1.118 [0.829,1.508] | 1.017 [0.743,1.392] |
| **Household size:** 1 |  |  | 1.000 - |  |  | 1.000 - |
| 2 |  |  | 0.934 [0.783,1.114] |  |  | 0.944 [0.792,1.126] |
| 3 |  |  | 1.094 [0.881,1.357] |  |  | 1.085 [0.874,1.346] |
| 4+ |  |  | 1.267^*^ [1.028,1.561] |  |  | 1.288^*^ [1.046,1.586] |
| **Socioeconomic deprivation:** Quartile 1 (most advantaged) |  |  | 1.000 - |  |  | 1.000 - |
| Quartile 2 |  |  | 1.102 [0.893,1.360] |  |  | 1.075 [0.871,1.327] |
| Quartile 3 |  |  | 1.248^*^ [1.018,1.531] |  |  | 1.178 [0.961,1.445] |
| Quartile 4 (least advantaged) |  |  | 1.545^***^ [1.251,1.907] |  |  | 1.387^**^ [1.122,1.714] |
| **Housing tenure:** Own |  |  | 1.000 - |  |  | 1.000 - |
| Rent/Other |  |  | 1.357^**^ [1.118,1.648] |  |  | 1.215 [0.999,1.477] |
| **Urban/rural:** Urban |  |  | 1.000 - |  |  | 1.000 - |
| Rural |  |  | 0.695^**^ [0.543,0.889] |  |  | 0.714^**^ [0.558,0.914] |
| **Employment status:** In paid employment or self-employed |  |  | 1.000 - |  |  | 1.000 - |
| Retired |  |  | 1.568^***^ [1.257,1.956] |  |  | 1.461^***^ [1.171,1.823] |
| Looking after home and/or family |  |  | 1.287 [0.798,2.076] |  |  | 1.227 [0.761,1.978] |
| Unable to work because of sickness or disability |  |  | 2.418^***^ [1.772,3.300] |  |  | 1.518^*^ [1.095,2.105] |
| Unemployed |  |  | 0.995 [0.577,1.718] |  |  | 0.936 [0.542,1.615] |
| Other |  |  | 1.413 [0.782,2.554] |  |  | 1.384 [0.766,2.502] |
| **Manual occupation:** Non-manual |  |  | 1.000 - |  |  | 1.000 - |
| Manual |  |  | 1.596^***^ [1.288,1.978] |  |  | 1.603^***^ [1.294,1.986] |
| Not in employment |  |  | - |  |  | - |
| **Healthcare worker status:** Non-healthcare worker |  |  | 1.000 - |  |  | 1.000 - |
| Healthcare worker |  |  | 4.799^***^ [3.954,5.823] |  |  | 4.753^***^ [3.915,5.771] |
| Not in employment |  |  | - |  |  | - |
| **Long-standing illness/disability:** No |  |  |  | 1.000 - |  | 1.000 - |
| Yes |  |  |  | 1.208^*^ [1.032,1.414] |  | 1.152 [0.983,1.349] |
| **Number of chronic conditions:** 0 |  |  |  | 1.000 - |  | 1.000 - |
| 1 |  |  |  | 1.047 [0.881,1.244] |  | 1.015 [0.854,1.206] |
| 2 |  |  |  | 1.270^*^ [1.040,1.551] |  | 1.171 [0.957,1.433] |
| 3 |  |  |  | 1.476^**^ [1.162,1.876] |  | 1.307^*^ [1.025,1.666] |
| 4+ |  |  |  | 1.548^**^ [1.155,2.074] |  | 1.297 [0.965,1.742] |
| **Self-reported health:** Excellent |  |  |  | 1.000 - |  | 1.000 - |
| Good |  |  |  | 1.036 [0.840,1.278] |  | 0.995 [0.806,1.228] |
| Fair |  |  |  | 1.338^*^ [1.052,1.702] |  | 1.208 [0.945,1.546] |
| Poor |  |  |  | 1.918^***^ [1.399,2.629] |  | 1.596^**^ [1.142,2.230] |
| **Body Mass Index:** Underweight (<18.5) |  |  |  |  | 1.920 [0.854,4.315] | 1.816 [0.810,4.067] |
| Normal weight (18.5-24.9) |  |  |  |  | 1.000 - | 1.000 - |
| Overweight (25.0-29.9) |  |  |  |  | 1.273^**^ [1.074,1.509] | 1.244^*^ [1.049,1.475] |
| Obese (>=30.0) |  |  |  |  | 1.787^***^ [1.497,2.133] | 1.536^***^ [1.278,1.847] |
| **Smoking status:** Never |  |  |  |  | 1.000 - | 1.000 - |
| Previous |  |  |  |  | 1.453^***^ [1.261,1.674] | 1.349^***^ [1.169,1.557] |
| Current |  |  |  |  | 1.311^*^ [1.054,1.631] | 1.111 [0.885,1.395] |
| **Alcohol consumption:** Daily or almost daily |  |  |  |  | 1.000 - | 1.000 - |
| Three or four times a week |  |  |  |  | 0.967 [0.779,1.200] | 0.958 [0.772,1.189] |
| Once or twice a week |  |  |  |  | 1.156 [0.941,1.419] | 1.104 [0.898,1.357] |
| One to three times a month |  |  |  |  | 1.415^**^ [1.114,1.796] | 1.271 [0.999,1.616] |
| Special occasions only |  |  |  |  | 1.322^*^ [1.037,1.686] | 1.107 [0.863,1.419] |
| Never (former drinker) |  |  |  |  | 1.927^***^ [1.425,2.605] | 1.461^*^ [1.074,1.987] |
| Never |  |  |  |  | 1.584^**^ [1.172,2.141] | 1.293 [0.953,1.754] |
| **Observations** | 392116 | 392116 | 392116 | 392116 | 392116 | 392116 |

Table S14: Education level and risk of testing positive in hospital

|  | **Model 1** | **Model 2** | **Model 3** | **Model 4** | **Model 5** | **Model 6** |
| --- | --- | --- | --- | --- | --- | --- |
| **Tested positive for SARS-CoV-2 in hospital** | **RR**  **[95% CI]** | **RR**  **[95% CI]** | **RR**  **[95% CI]** | **RR**  **[95% CI]** | **RR**  **[95% CI]** | **RR**  **[95% CI]** |
| **Age group:** 40-44 | 1.000 - | 1.000 - | 1.000 - | 1.000 - | 1.000 - | 1.000 - |
| 45-49 | 0.916 [0.684,1.227] | 0.934 [0.697,1.252] | 0.944 [0.704,1.265] | 0.908 [0.678,1.218] | 0.931 [0.695,1.249] | 0.921 [0.687,1.233] |
| 50-54 | 0.618^**^ [0.454,0.842] | 0.652^**^ [0.477,0.889] | 0.694^*^ [0.509,0.946] | 0.611^**^ [0.448,0.834] | 0.638^**^ [0.468,0.872] | 0.647^**^ [0.474,0.882] |
| 55-59 | 0.645^**^ [0.482,0.864] | 0.703^*^ [0.523,0.944] | 0.803 [0.597,1.079] | 0.639^**^ [0.477,0.856] | 0.678^*^ [0.504,0.912] | 0.725^*^ [0.541,0.973] |
| 60-64 | 0.696^**^ [0.531,0.911] | 0.781 [0.593,1.029] | 0.956 [0.711,1.284] | 0.711^*^ [0.540,0.936] | 0.749^*^ [0.566,0.992] | 0.854 [0.632,1.152] |
| 65-69 | 1.020 [0.781,1.331] | 1.144 [0.873,1.500] | 1.434^*^ [1.041,1.975] | 1.031 [0.785,1.356] | 1.096 [0.831,1.445] | 1.258 [0.906,1.745] |
| 70+ | 2.040^*^ [1.056,3.941] | 2.271^*^ [1.173,4.394] | 2.829^**^ [1.435,5.574] | 2.054^*^ [1.058,3.986] | 2.182^*^ [1.125,4.232] | 2.516^**^ [1.270,4.985] |
| **Sex:** Female | 1.000 - | 1.000 - | 1.000 - | 1.000 - | 1.000 - | 1.000 - |
| Male | 1.443^***^ [1.247,1.670] | 1.441^***^ [1.245,1.668] | 1.617^***^ [1.387,1.884] | 1.417^***^ [1.225,1.640] | 1.408^***^ [1.209,1.639] | 1.556^***^ [1.330,1.822] |
| **Assessment centre:** Manchester | 1.157 [0.781,1.714] | 1.043 [0.703,1.548] | 0.987 [0.665,1.465] | 1.038 [0.700,1.541] | 1.023 [0.689,1.519] | 0.985 [0.663,1.462] |
| Oxford | 0.650 [0.395,1.070] | 0.632 [0.384,1.041] | 0.752 [0.454,1.244] | 0.672 [0.408,1.108] | 0.659 [0.400,1.087] | 0.784 [0.473,1.299] |
| Stoke | 0.769 [0.522,1.132] | 0.770 [0.523,1.133] | 0.853 [0.580,1.255] | 0.779 [0.529,1.147] | 0.765 [0.520,1.127] | 0.835 [0.568,1.229] |
| Reading | 0.370^***^ [0.233,0.586] | 0.354^***^ [0.224,0.562] | 0.441^***^ [0.278,0.701] | 0.388^***^ [0.244,0.615] | 0.367^***^ [0.231,0.581] | 0.458^***^ [0.288,0.728] |
| Bury | 0.575^**^ [0.393,0.840] | 0.551^**^ [0.377,0.806] | 0.571^**^ [0.390,0.836] | 0.549^**^ [0.375,0.803] | 0.547^**^ [0.374,0.801] | 0.560^**^ [0.382,0.820] |
| Newcastle | 1.000 - | 1.000 - | 1.000 - | 1.000 - | 1.000 - | 1.000 - |
| Leeds | 0.612^**^ [0.443,0.847] | 0.590^**^ [0.427,0.815] | 0.628^**^ [0.454,0.868] | 0.610^**^ [0.442,0.844] | 0.592^**^ [0.428,0.818] | 0.632^**^ [0.457,0.874] |
| Bristol | 0.334^***^ [0.222,0.501] | 0.328^***^ [0.219,0.492] | 0.368^***^ [0.245,0.553] | 0.344^***^ [0.229,0.517] | 0.331^***^ [0.221,0.497] | 0.371^***^ [0.247,0.559] |
| Barts | 0.890 [0.554,1.430] | 0.700 [0.432,1.134] | 0.549^*^ [0.339,0.891] | 0.718 [0.443,1.162] | 0.686 [0.423,1.112] | 0.596^*^ [0.367,0.968] |
| Nottingham | 0.468^***^ [0.319,0.688] | 0.454^***^ [0.309,0.667] | 0.506^***^ [0.344,0.745] | 0.468^***^ [0.319,0.687] | 0.457^***^ [0.311,0.671] | 0.506^***^ [0.344,0.745] |
| Sheffield | 0.790 [0.567,1.102] | 0.774 [0.555,1.079] | 0.795 [0.570,1.109] | 0.765 [0.549,1.067] | 0.772 [0.554,1.077] | 0.778 [0.558,1.085] |
| Liverpool | 0.985 [0.725,1.338] | 0.965 [0.711,1.311] | 0.936 [0.689,1.272] | 0.954 [0.702,1.296] | 0.943 [0.694,1.282] | 0.917 [0.674,1.247] |
| Middlesborough | 0.770 [0.529,1.123] | 0.769 [0.528,1.120] | 0.820 [0.562,1.195] | 0.756 [0.519,1.101] | 0.756 [0.519,1.102] | 0.790 [0.542,1.152] |
| Hounslow | 1.015 [0.730,1.413] | 0.815 [0.578,1.148] | 0.778 [0.550,1.099] | 0.845 [0.599,1.191] | 0.819 [0.581,1.156] | 0.810 [0.572,1.146] |
| Croydon | 0.861 [0.607,1.222] | 0.679^*^ [0.476,0.968] | 0.651^*^ [0.456,0.929] | 0.695^*^ [0.487,0.991] | 0.684^*^ [0.480,0.975] | 0.670^*^ [0.469,0.957] |
| Birmingham | 0.894 [0.636,1.256] | 0.761 [0.538,1.076] | 0.731 [0.517,1.034] | 0.761 [0.538,1.077] | 0.743 [0.525,1.051] | 0.721 [0.509,1.022] |
| **Education level:** College or University degree | 1.000 - | 1.000 - | 1.000 - | 1.000 - | 1.000 - | 1.000 - |
| A levels/AS levels | 1.187 [0.898,1.568] | 1.216 [0.920,1.607] | 1.248 [0.945,1.648] | 1.163 [0.880,1.537] | 1.151 [0.870,1.523] | 1.178 [0.891,1.557] |
| O levels/GCSEs/CSEs | 1.194 [0.965,1.478] | 1.204 [0.973,1.490] | 1.183 [0.952,1.471] | 1.125 [0.908,1.393] | 1.093 [0.881,1.356] | 1.082 [0.869,1.347] |
| Other | 1.655^***^ [1.294,2.118] | 1.591^***^ [1.244,2.035] | 1.352^*^ [1.052,1.738] | 1.466^**^ [1.145,1.875] | 1.412^**^ [1.102,1.808] | 1.210 [0.940,1.557] |
| None of the above | 2.229^***^ [1.801,2.760] | 2.163^***^ [1.752,2.672] | 1.827^***^ [1.455,2.293] | 1.828^***^ [1.471,2.271] | 1.791^***^ [1.438,2.231] | 1.575^***^ [1.247,1.990] |
| **Ethnicity:** White British |  | 1.000 - | 1.000 - | 1.000 - | 1.000 - | 1.000 - |
| White Irish |  | 1.539^*^ [1.045,2.265] | 1.369 [0.929,2.018] | 1.495^*^ [1.015,2.202] | 1.469 [0.997,2.166] | 1.343 [0.910,1.982] |
| White Other |  | 1.009 [0.586,1.739] | 0.982 [0.569,1.693] | 0.979 [0.570,1.682] | 0.949 [0.551,1.633] | 0.939 [0.547,1.613] |
| Mixed |  | 1.359 [0.554,3.333] | 1.179 [0.481,2.893] | 1.292 [0.525,3.175] | 1.246 [0.507,3.065] | 1.128 [0.458,2.778] |
| South Asian |  | 2.518^***^ [1.550,4.091] | 2.240^**^ [1.373,3.655] | 2.226^**^ [1.365,3.628] | 2.281^***^ [1.408,3.698] | 2.014^**^ [1.236,3.283] |
| Black |  | 3.331^***^ [2.161,5.136] | 2.437^***^ [1.557,3.816] | 3.055^***^ [1.979,4.716] | 2.900^***^ [1.877,4.481] | 2.276^***^ [1.454,3.562] |
| Chinese |  | 1.811 [0.562,5.836] | 1.810 [0.562,5.825] | 1.880 [0.584,6.048] | 1.950 [0.606,6.273] | 2.036 [0.633,6.544] |
| Other |  | 2.426^**^ [1.348,4.365] | 1.892^*^ [1.042,3.436] | 2.213^**^ [1.231,3.978] | 2.063^*^ [1.150,3.700] | 1.742 [0.967,3.141] |
| **Country of birth:** UK & Ireland |  | 1.000 - | 1.000 - | 1.000 - | 1.000 - | 1.000 - |
| Elsewhere |  | 0.947 [0.665,1.348] | 0.844 [0.584,1.221] | 0.956 [0.671,1.362] | 0.945 [0.664,1.345] | 0.870 [0.602,1.255] |
| **Household size:** 1 |  |  | 1.000 - |  |  | 1.000 - |
| 2 |  |  | 0.946 [0.776,1.154] |  |  | 0.953 [0.781,1.163] |
| 3 |  |  | 1.079 [0.845,1.377] |  |  | 1.067 [0.837,1.362] |
| 4+ |  |  | 1.205 [0.946,1.533] |  |  | 1.223 [0.961,1.555] |
| **Socioeconomic deprivation:** Quartile 1 (most advantaged) |  |  | 1.000 - |  |  | 1.000 - |
| Quartile 2 |  |  | 1.238 [0.970,1.580] |  |  | 1.204 [0.943,1.538] |
| Quartile 3 |  |  | 1.333^*^ [1.048,1.695] |  |  | 1.253 [0.985,1.592] |
| Quartile 4 (least advantaged) |  |  | 1.736^***^ [1.359,2.217] |  |  | 1.544^***^ [1.208,1.972] |
| **Housing tenure:** Own |  |  | 1.000 - |  |  | 1.000 - |
| Rent/Other |  |  | 1.291^*^ [1.034,1.611] |  |  | 1.148 [0.918,1.437] |
| **Urban/rural:** Urban |  |  | 1.000 - |  |  | 1.000 - |
| Rural |  |  | 0.667^**^ [0.500,0.889] |  |  | 0.684^**^ [0.513,0.913] |
| **Employment status:** In paid employment or self-employed |  |  | 1.000 - |  |  | 1.000 - |
| Retired |  |  | 1.417^**^ [1.111,1.807] |  |  | 1.310^*^ [1.027,1.670] |
| Looking after home and/or family |  |  | 1.203 [0.691,2.096] |  |  | 1.138 [0.654,1.980] |
| Unable to work because of sickness or disability |  |  | 2.186^***^ [1.546,3.090] |  |  | 1.341 [0.935,1.922] |
| Unemployed |  |  | 0.771 [0.391,1.519] |  |  | 0.720 [0.365,1.418] |
| Other |  |  | 1.599 [0.860,2.973] |  |  | 1.558 [0.837,2.899] |
| **Manual occupation:** Non-manual |  |  | 1.000 - |  |  | 1.000 - |
| Manual |  |  | 1.509^**^ [1.176,1.937] |  |  | 1.522^***^ [1.186,1.954] |
| Not in employment |  |  | - |  |  | - |
| **Healthcare worker status:** Non-healthcare worker |  |  | 1.000 - |  |  | 1.000 - |
| Healthcare worker |  |  | 3.903^***^ [3.100,4.915] |  |  | 3.866^***^ [3.068,4.871] |
| Not in employment |  |  | - |  |  | - |
| **Long-standing illness/disability:** No |  |  |  | 1.000 - |  | 1.000 - |
| Yes |  |  |  | 1.221^*^ [1.022,1.460] |  | 1.170 [0.977,1.401] |
| **Number of chronic conditions:** 0 |  |  |  | 1.000 - |  | 1.000 - |
| 1 |  |  |  | 1.083 [0.888,1.321] |  | 1.048 [0.859,1.279] |
| 2 |  |  |  | 1.322^*^ [1.054,1.658] |  | 1.218 [0.968,1.532] |
| 3 |  |  |  | 1.533^**^ [1.170,2.009] |  | 1.352^*^ [1.027,1.781] |
| 4+ |  |  |  | 1.469^*^ [1.052,2.051] |  | 1.227 [0.876,1.719] |
| **Self-reported health:** Excellent |  |  |  | 1.000 - |  | 1.000 - |
| Good |  |  |  | 1.151 [0.895,1.479] |  | 1.095 [0.852,1.408] |
| Fair |  |  |  | 1.473^**^ [1.109,1.957] |  | 1.312 [0.983,1.752] |
| Poor |  |  |  | 2.208^***^ [1.538,3.170] |  | 1.822^**^ [1.246,2.666] |
| **Body Mass Index:** Underweight (<18.5) |  |  |  |  | 2.269 [0.930,5.536] | 2.145 [0.881,5.219] |
| Normal weight (18.5-24.9) |  |  |  |  | 1.000 - | 1.000 - |
| Overweight (25.0-29.9) |  |  |  |  | 1.341^**^ [1.100,1.634] | 1.296^*^ [1.063,1.580] |
| Obese (>=30.0) |  |  |  |  | 1.914^***^ [1.559,2.349] | 1.612^***^ [1.302,1.995] |
| **Smoking status:** Never |  |  |  |  | 1.000 - | 1.000 - |
| Previous |  |  |  |  | 1.518^***^ [1.291,1.785] | 1.404^***^ [1.192,1.655] |
| Current |  |  |  |  | 1.355^*^ [1.055,1.741] | 1.142 [0.881,1.479] |
| **Alcohol consumption:** Daily or almost daily |  |  |  |  | 1.000 - | 1.000 - |
| Three or four times a week |  |  |  |  | 0.982 [0.770,1.251] | 0.977 [0.766,1.245] |
| Once or twice a week |  |  |  |  | 1.080 [0.854,1.365] | 1.030 [0.814,1.303] |
| One to three times a month |  |  |  |  | 1.352^*^ [1.028,1.778] | 1.217 [0.924,1.603] |
| Special occasions only |  |  |  |  | 1.265 [0.958,1.669] | 1.061 [0.800,1.408] |
| Never (former drinker) |  |  |  |  | 1.846^***^ [1.309,2.602] | 1.405 [0.989,1.996] |
| Never |  |  |  |  | 1.668^**^ [1.194,2.331] | 1.373 [0.979,1.926] |
| **Observations** | 392116 | 392116 | 392116 | 392116 | 392116 | 392116 |

Table S15: Education level and risk of being tested

|  | **Model 1** | **Model 2** | **Model 3** | **Model 4** | **Model 5** | **Model 6** |
| --- | --- | --- | --- | --- | --- | --- |
| **Tested for SARS-CoV-2** | **RR**  **[95% CI]** | **RR**  **[95% CI]** | **RR**  **[95% CI]** | **RR**  **[95% CI]** | **RR**  **[95% CI]** | **RR**  **[95% CI]** |
| **Age group:** 40-44 | 1.000 - | 1.000 - | 1.000 - | 1.000 - | 1.000 - | 1.000 - |
| 45-49 | 0.789^**^ [0.680,0.915] | 0.796^**^ [0.686,0.923] | 0.795^**^ [0.685,0.922] | 0.779^***^ [0.671,0.903] | 0.798^**^ [0.688,0.926] | 0.782^**^ [0.674,0.907] |
| 50-54 | 0.610^***^ [0.524,0.710] | 0.624^***^ [0.535,0.727] | 0.645^***^ [0.554,0.752] | 0.593^***^ [0.509,0.691] | 0.619^***^ [0.531,0.722] | 0.617^***^ [0.529,0.719] |
| 55-59 | 0.553^***^ [0.476,0.642] | 0.574^***^ [0.494,0.668] | 0.632^***^ [0.543,0.736] | 0.533^***^ [0.459,0.619] | 0.568^***^ [0.488,0.661] | 0.594^***^ [0.510,0.691] |
| 60-64 | 0.650^***^ [0.568,0.744] | 0.685^***^ [0.598,0.785] | 0.819^**^ [0.704,0.953] | 0.639^***^ [0.557,0.733] | 0.678^***^ [0.591,0.778] | 0.768^***^ [0.659,0.895] |
| 65-69 | 0.957 [0.837,1.095] | 1.007 [0.880,1.153] | 1.258^**^ [1.061,1.492] | 0.930 [0.811,1.068] | 1.000 [0.871,1.148] | 1.163 [0.978,1.383] |
| 70+ | 1.510^*^ [1.029,2.215] | 1.581^*^ [1.077,2.320] | 1.970^***^ [1.323,2.934] | 1.464 [0.997,2.149] | 1.575^*^ [1.072,2.314] | 1.835^**^ [1.232,2.734] |
| **Sex:** Female | 1.000 - | 1.000 - | 1.000 - | 1.000 - | 1.000 - | 1.000 - |
| Male | 1.141^***^ [1.058,1.232] | 1.141^***^ [1.058,1.232] | 1.274^***^ [1.177,1.380] | 1.125^**^ [1.042,1.214] | 1.133^**^ [1.046,1.226] | 1.247^***^ [1.148,1.354] |
| **Assessment centre:** Manchester | 1.198 [0.962,1.491] | 1.141 [0.915,1.422] | 1.084 [0.869,1.351] | 1.132 [0.908,1.411] | 1.113 [0.892,1.387] | 1.074 [0.861,1.339] |
| Oxford | 0.926 [0.727,1.180] | 0.913 [0.717,1.163] | 1.031 [0.808,1.316] | 0.961 [0.755,1.225] | 0.929 [0.730,1.183] | 1.047 [0.820,1.336] |
| Stoke | 0.781^*^ [0.626,0.976] | 0.781^*^ [0.626,0.976] | 0.838 [0.672,1.047] | 0.787^*^ [0.630,0.982] | 0.775^*^ [0.621,0.968] | 0.822 [0.658,1.025] |
| Reading | 0.692^***^ [0.563,0.851] | 0.678^***^ [0.552,0.834] | 0.795^*^ [0.646,0.980] | 0.734^**^ [0.597,0.903] | 0.690^***^ [0.561,0.848] | 0.815 [0.662,1.003] |
| Bury | 0.797^*^ [0.656,0.970] | 0.783^*^ [0.644,0.953] | 0.798^*^ [0.656,0.970] | 0.778^*^ [0.640,0.946] | 0.774^*^ [0.637,0.941] | 0.781^*^ [0.642,0.950] |
| Newcastle | 1.000 - | 1.000 - | 1.000 - | 1.000 - | 1.000 - | 1.000 - |
| Leeds | 0.379^***^ [0.305,0.470] | 0.372^***^ [0.300,0.462] | 0.389^***^ [0.314,0.483] | 0.384^***^ [0.309,0.476] | 0.372^***^ [0.300,0.461] | 0.391^***^ [0.315,0.485] |
| Bristol | 0.666^***^ [0.554,0.801] | 0.660^***^ [0.550,0.793] | 0.711^***^ [0.591,0.855] | 0.689^***^ [0.573,0.828] | 0.661^***^ [0.550,0.794] | 0.711^***^ [0.591,0.855] |
| Barts | 1.245 [0.988,1.569] | 1.101 [0.871,1.393] | 0.917 [0.724,1.163] | 1.125 [0.890,1.423] | 1.060 [0.838,1.341] | 0.970 [0.765,1.229] |
| Nottingham | 0.695^***^ [0.573,0.842] | 0.684^***^ [0.565,0.830] | 0.738^**^ [0.608,0.894] | 0.704^***^ [0.581,0.853] | 0.684^***^ [0.564,0.829] | 0.736^**^ [0.607,0.892] |
| Sheffield | 1.178 [0.994,1.397] | 1.167 [0.984,1.383] | 1.179 [0.995,1.397] | 1.152 [0.972,1.366] | 1.161 [0.979,1.376] | 1.150 [0.971,1.362] |
| Liverpool | 1.042 [0.877,1.239] | 1.034 [0.870,1.229] | 0.999 [0.840,1.187] | 1.017 [0.856,1.209] | 1.011 [0.851,1.202] | 0.979 [0.824,1.163] |
| Middlesborough | 1.006 [0.826,1.225] | 1.005 [0.825,1.223] | 1.036 [0.850,1.262] | 0.987 [0.811,1.202] | 0.990 [0.813,1.205] | 1.005 [0.825,1.224] |
| Hounslow | 0.949 [0.785,1.148] | 0.857 [0.706,1.040] | 0.833 [0.686,1.011] | 0.886 [0.730,1.075] | 0.848 [0.699,1.030] | 0.850 [0.700,1.032] |
| Croydon | 1.061 [0.882,1.277] | 0.942 [0.782,1.135] | 0.917 [0.760,1.105] | 0.962 [0.798,1.159] | 0.933 [0.774,1.124] | 0.926 [0.768,1.116] |
| Birmingham | 0.904 [0.745,1.097] | 0.839 [0.690,1.020] | 0.809^*^ [0.666,0.984] | 0.839 [0.690,1.020] | 0.816^*^ [0.671,0.992] | 0.799^*^ [0.657,0.972] |
| **Education level:** College or University degree | 1.000 - | 1.000 - | 1.000 - | 1.000 - | 1.000 - | 1.000 - |
| A levels/AS levels | 1.047 [0.911,1.203] | 1.058 [0.921,1.215] | 1.084 [0.943,1.245] | 1.020 [0.888,1.172] | 1.017 [0.885,1.169] | 1.045 [0.910,1.201] |
| O levels/GCSEs/CSEs | 1.015 [0.912,1.129] | 1.018 [0.914,1.133] | 1.019 [0.914,1.135] | 0.960 [0.862,1.069] | 0.947 [0.850,1.055] | 0.963 [0.862,1.074] |
| Other | 1.263^***^ [1.110,1.438] | 1.234^**^ [1.084,1.405] | 1.067 [0.937,1.216] | 1.147^*^ [1.007,1.306] | 1.132 [0.993,1.289] | 0.994 [0.871,1.134] |
| None of the above | 1.663^***^ [1.489,1.857] | 1.639^***^ [1.468,1.830] | 1.431^***^ [1.272,1.609] | 1.404^***^ [1.255,1.570] | 1.420^***^ [1.268,1.590] | 1.291^***^ [1.145,1.457] |
| **Ethnicity:** White British |  | 1.000 - | 1.000 - | 1.000 - | 1.000 - | 1.000 - |
| White Irish |  | 1.239 [0.994,1.545] | 1.099 [0.880,1.372] | 1.205 [0.966,1.502] | 1.199 [0.961,1.496] | 1.086 [0.870,1.356] |
| White Other |  | 1.020 [0.788,1.320] | 0.997 [0.770,1.290] | 0.993 [0.769,1.282] | 0.972 [0.751,1.257] | 0.967 [0.749,1.249] |
| Mixed |  | 1.149 [0.720,1.832] | 1.009 [0.633,1.607] | 1.092 [0.685,1.743] | 1.064 [0.667,1.699] | 0.975 [0.612,1.555] |
| South Asian |  | 1.492^**^ [1.117,1.992] | 1.351^*^ [1.009,1.808] | 1.334 [0.997,1.784] | 1.387^*^ [1.035,1.858] | 1.252 [0.933,1.681] |
| Black |  | 2.060^***^ [1.600,2.652] | 1.517^**^ [1.166,1.972] | 1.898^***^ [1.473,2.446] | 1.852^***^ [1.436,2.389] | 1.475^**^ [1.134,1.918] |
| Chinese |  | 0.532 [0.197,1.436] | 0.534 [0.197,1.442] | 0.550 [0.204,1.484] | 0.537 [0.199,1.450] | 0.558 [0.206,1.508] |
| Other |  | 1.562^*^ [1.089,2.241] | 1.229 [0.852,1.773] | 1.437^*^ [1.003,2.060] | 1.384 [0.966,1.981] | 1.176 [0.819,1.688] |
| **Country of birth:** UK & Ireland |  | 1.000 - | 1.000 - | 1.000 - | 1.000 - | 1.000 - |
| Elsewhere |  | 1.026 [0.842,1.250] | 0.943 [0.768,1.157] | 1.035 [0.850,1.261] | 1.020 [0.837,1.242] | 0.964 [0.787,1.181] |
| **Household size:** 1 |  |  | 1.000 - |  |  | 1.000 - |
| 2 |  |  | 0.844^**^ [0.762,0.935] |  |  | 0.859^**^ [0.776,0.952] |
| 3 |  |  | 1.016 [0.895,1.153] |  |  | 1.020 [0.899,1.157] |
| 4+ |  |  | 1.041 [0.918,1.180] |  |  | 1.071 [0.945,1.214] |
| **Socioeconomic deprivation:** Quartile 1 (most advantaged) |  |  | 1.000 - |  |  | 1.000 - |
| Quartile 2 |  |  | 1.076 [0.954,1.212] |  |  | 1.056 [0.936,1.190] |
| Quartile 3 |  |  | 1.204^**^ [1.070,1.354] |  |  | 1.148^*^ [1.020,1.292] |
| Quartile 4 (least advantaged) |  |  | 1.331^***^ [1.176,1.507] |  |  | 1.212^**^ [1.070,1.374] |
| **Housing tenure:** Own |  |  | 1.000 - |  |  | 1.000 - |
| Rent/Other |  |  | 1.348^***^ [1.199,1.516] |  |  | 1.207^**^ [1.072,1.358] |
| **Urban/rural:** Urban |  |  | 1.000 - |  |  | 1.000 - |
| Rural |  |  | 0.788^***^ [0.690,0.899] |  |  | 0.800^***^ [0.701,0.913] |
| **Employment status:** In paid employment or self-employed |  |  | 1.000 - |  |  | 1.000 - |
| Retired |  |  | 1.349^***^ [1.183,1.537] |  |  | 1.257^***^ [1.103,1.433] |
| Looking after home and/or family |  |  | 1.013 [0.751,1.367] |  |  | 0.964 [0.714,1.300] |
| Unable to work because of sickness or disability |  |  | 2.630^***^ [2.205,3.137] |  |  | 1.620^***^ [1.337,1.964] |
| Unemployed |  |  | 1.273 [0.949,1.707] |  |  | 1.186 [0.885,1.590] |
| Other |  |  | 1.652^**^ [1.204,2.268] |  |  | 1.602^**^ [1.167,2.199] |
| **Manual occupation:** Non-manual |  |  | 1.000 - |  |  | 1.000 - |
| Manual |  |  | 1.314^***^ [1.145,1.509] |  |  | 1.314^***^ [1.145,1.508] |
| Not in employment |  |  | - |  |  | - |
| **Healthcare worker status:** Non-healthcare worker |  |  | 1.000 - |  |  | 1.000 - |
| Healthcare worker |  |  | 3.911^***^ [3.478,4.399] |  |  | 3.890^***^ [3.459,4.376] |
| Not in employment |  |  | - |  |  | - |
| **Long-standing illness/disability:** No |  |  |  | 1.000 - |  | 1.000 - |
| Yes |  |  |  | 1.231^***^ [1.121,1.352] |  | 1.176^***^ [1.070,1.293] |
| **Number of chronic conditions:** 0 |  |  |  | 1.000 - |  | 1.000 - |
| 1 |  |  |  | 1.034 [0.934,1.145] |  | 1.015 [0.916,1.124] |
| 2 |  |  |  | 1.172^*^ [1.038,1.322] |  | 1.113 [0.985,1.257] |
| 3 |  |  |  | 1.329^***^ [1.145,1.541] |  | 1.227^**^ [1.056,1.424] |
| 4+ |  |  |  | 1.597^***^ [1.346,1.895] |  | 1.414^***^ [1.190,1.681] |
| **Self-reported health:** Excellent |  |  |  | 1.000 - |  | 1.000 - |
| Good |  |  |  | 1.010 [0.893,1.141] |  | 0.998 [0.883,1.128] |
| Fair |  |  |  | 1.325^***^ [1.148,1.528] |  | 1.247^**^ [1.078,1.443] |
| Poor |  |  |  | 1.956^***^ [1.622,2.359] |  | 1.647^***^ [1.349,2.010] |
| **Body Mass Index:** Underweight (<18.5) |  |  |  |  | 1.460 [0.892,2.389] | 1.352 [0.826,2.212] |
| Normal weight (18.5-24.9) |  |  |  |  | 1.000 - | 1.000 - |
| Overweight (25.0-29.9) |  |  |  |  | 1.111^*^ [1.009,1.223] | 1.084 [0.984,1.193] |
| Obese (>=30.0) |  |  |  |  | 1.436^***^ [1.296,1.590] | 1.216^***^ [1.093,1.353] |
| **Smoking status:** Never |  |  |  |  | 1.000 - | 1.000 - |
| Previous |  |  |  |  | 1.288^***^ [1.184,1.402] | 1.201^***^ [1.103,1.309] |
| Current |  |  |  |  | 1.456^***^ [1.288,1.645] | 1.213^**^ [1.069,1.378] |
| **Alcohol consumption:** Daily or almost daily |  |  |  |  | 1.000 - | 1.000 - |
| Three or four times a week |  |  |  |  | 0.921 [0.815,1.041] | 0.915 [0.810,1.034] |
| Once or twice a week |  |  |  |  | 0.989 [0.878,1.113] | 0.945 [0.839,1.064] |
| One to three times a month |  |  |  |  | 1.159^*^ [1.006,1.335] | 1.044 [0.906,1.203] |
| Special occasions only |  |  |  |  | 1.231^**^ [1.070,1.415] | 1.033 [0.897,1.190] |
| Never (former drinker) |  |  |  |  | 1.582^***^ [1.317,1.901] | 1.176 [0.976,1.416] |
| Never |  |  |  |  | 1.348^**^ [1.116,1.628] | 1.108 [0.916,1.340] |
| **Observations** | 392116 | 392116 | 392116 | 392116 | 392116 | 392116 |

Table S16: Ethnicity and risk of testing positive amongst those tested

|  | **Model 1** | **Model 2** | **Model 3** | **Model 4** | **Model 5** | **Model 6** | **Model 7** |
| --- | --- | --- | --- | --- | --- | --- | --- |
| **Tested positive for SARS-CoV-2** | **RR**  **[95% CI]** | **RR**  **[95% CI]** | **RR**  **[95% CI]** | **RR**  **[95% CI]** | **RR**  **[95% CI]** | **RR**  **[95% CI]** | **RR**  **[95% CI]** |
| **Age group:** 40-44 | 1.000 - | 1.000 - | 1.000 - | 1.000 - | 1.000 - | 1.000 - | 1.000 - |
| 45-49 | 0.968 [0.804,1.166] | 0.968 [0.803,1.165] | 0.989 [0.820,1.192] | 0.967 [0.802,1.166] | 0.960 [0.796,1.157] | 0.972 [0.807,1.170] | 0.972 [0.806,1.174] |
| 50-54 | 0.892 [0.733,1.086] | 0.888 [0.729,1.082] | 0.920 [0.753,1.123] | 0.907 [0.742,1.109] | 0.876 [0.717,1.070] | 0.893 [0.734,1.086] | 0.906 [0.741,1.109] |
| 55-59 | 0.904 [0.743,1.098] | 0.901 [0.741,1.095] | 0.964 [0.786,1.182] | 0.956 [0.777,1.176] | 0.874 [0.716,1.067] | 0.888 [0.731,1.080] | 0.933 [0.755,1.151] |
| 60-64 | 0.822^*^ [0.687,0.983] | 0.819^*^ [0.684,0.980] | 0.894 [0.733,1.091] | 0.851 [0.684,1.058] | 0.795^*^ [0.661,0.956] | 0.806^*^ [0.673,0.967] | 0.833 [0.666,1.040] |
| 65-69 | 0.966 [0.817,1.142] | 0.963 [0.815,1.139] | 1.062 [0.870,1.295] | 0.986 [0.779,1.246] | 0.925 [0.777,1.102] | 0.942 [0.795,1.116] | 0.958 [0.754,1.219] |
| 70+ | 1.135 [0.723,1.783] | 1.131 [0.720,1.776] | 1.241 [0.778,1.981] | 1.143 [0.697,1.872] | 1.065 [0.673,1.684] | 1.098 [0.692,1.741] | 1.084 [0.652,1.803] |
| **Sex:** Female | 1.000 - | 1.000 - | 1.000 - | 1.000 - | 1.000 - | 1.000 - | 1.000 - |
| Male | 1.212^***^ [1.094,1.342] | 1.213^***^ [1.095,1.344] | 1.244^***^ [1.120,1.381] | 1.246^***^ [1.118,1.387] | 1.217^***^ [1.098,1.348] | 1.205^***^ [1.084,1.339] | 1.240^***^ [1.109,1.385] |
| **Assessment centre:** Manchester | 0.908 [0.686,1.204] | 0.908 [0.685,1.203] | 0.907 [0.684,1.204] | 0.908 [0.687,1.200] | 0.910 [0.686,1.205] | 0.925 [0.699,1.225] | 0.921 [0.698,1.217] |
| Oxford | 0.626^*^ [0.421,0.929] | 0.624^*^ [0.420,0.926] | 0.614^*^ [0.412,0.913] | 0.665^*^ [0.449,0.985] | 0.626^*^ [0.421,0.930] | 0.666^*^ [0.448,0.990] | 0.694 [0.468,1.030] |
| Stoke | 0.970 [0.733,1.284] | 0.971 [0.734,1.285] | 0.972 [0.735,1.286] | 0.977 [0.741,1.287] | 0.978 [0.739,1.295] | 0.967 [0.732,1.277] | 0.980 [0.744,1.290] |
| Reading | 0.824 [0.622,1.092] | 0.822 [0.621,1.088] | 0.822 [0.621,1.087] | 0.863 [0.651,1.145] | 0.827 [0.624,1.095] | 0.855 [0.646,1.130] | 0.884 [0.666,1.173] |
| Bury | 0.832 [0.640,1.083] | 0.831 [0.639,1.082] | 0.840 [0.645,1.094] | 0.839 [0.646,1.090] | 0.830 [0.638,1.080] | 0.833 [0.641,1.082] | 0.837 [0.644,1.086] |
| Newcastle | 1.000 - | 1.000 - | 1.000 - | 1.000 - | 1.000 - | 1.000 - | 1.000 - |
| Leeds | 1.638^***^ [1.343,1.997] | 1.637^***^ [1.343,1.997] | 1.640^***^ [1.345,1.999] | 1.703^***^ [1.396,2.078] | 1.652^***^ [1.354,2.017] | 1.648^***^ [1.349,2.015] | 1.713^***^ [1.401,2.096] |
| Bristol | 0.597^***^ [0.447,0.798] | 0.596^***^ [0.447,0.796] | 0.595^***^ [0.446,0.794] | 0.610^***^ [0.457,0.814] | 0.596^***^ [0.446,0.796] | 0.599^***^ [0.448,0.800] | 0.609^***^ [0.456,0.814] |
| Barts | 0.662^*^ [0.475,0.924] | 0.660^*^ [0.473,0.921] | 0.659^*^ [0.472,0.920] | 0.647^*^ [0.462,0.908] | 0.660^*^ [0.473,0.922] | 0.706^*^ [0.505,0.986] | 0.685^*^ [0.488,0.964] |
| Nottingham | 0.725^*^ [0.548,0.959] | 0.725^*^ [0.548,0.959] | 0.728^*^ [0.550,0.964] | 0.756^*^ [0.572,0.998] | 0.723^*^ [0.545,0.958] | 0.732^*^ [0.553,0.970] | 0.754 [0.568,1.001] |
| Sheffield | 0.722^**^ [0.568,0.918] | 0.721^**^ [0.567,0.917] | 0.712^**^ [0.559,0.906] | 0.735^*^ [0.578,0.934] | 0.720^**^ [0.566,0.916] | 0.731^**^ [0.576,0.928] | 0.737^*^ [0.581,0.935] |
| Liverpool | 0.929 [0.744,1.160] | 0.929 [0.744,1.159] | 0.926 [0.742,1.156] | 0.928 [0.745,1.157] | 0.927 [0.742,1.158] | 0.928 [0.743,1.159] | 0.927 [0.743,1.157] |
| Middlesborough | 0.708^*^ [0.528,0.948] | 0.707^*^ [0.528,0.947] | 0.705^*^ [0.527,0.944] | 0.730^*^ [0.547,0.974] | 0.706^*^ [0.527,0.946] | 0.707^*^ [0.528,0.946] | 0.725^*^ [0.543,0.968] |
| Hounslow | 0.942 [0.741,1.198] | 0.937 [0.737,1.192] | 0.944 [0.742,1.200] | 0.959 [0.753,1.222] | 0.943 [0.742,1.199] | 0.961 [0.756,1.221] | 0.985 [0.773,1.254] |
| Croydon | 0.976 [0.773,1.232] | 0.970 [0.768,1.226] | 0.976 [0.772,1.234] | 1.024 [0.809,1.297] | 0.975 [0.772,1.232] | 0.996 [0.788,1.260] | 1.043 [0.823,1.322] |
| Birmingham | 0.930 [0.727,1.189] | 0.932 [0.729,1.191] | 0.929 [0.726,1.188] | 0.920 [0.719,1.178] | 0.924 [0.722,1.183] | 0.941 [0.736,1.204] | 0.927 [0.722,1.189] |
| **Ethnicity:** White British | 1.000 - | 1.000 - | 1.000 - | 1.000 - | 1.000 - | 1.000 - | 1.000 - |
| White Irish | 1.166 [0.877,1.549] | 1.165 [0.877,1.548] | 1.179 [0.889,1.565] | 1.133 [0.853,1.505] | 1.169 [0.877,1.556] | 1.150 [0.866,1.527] | 1.132 [0.851,1.507] |
| White Other | 1.093 [0.814,1.467] | 1.034 [0.737,1.452] | 1.014 [0.718,1.430] | 1.020 [0.718,1.448] | 1.045 [0.743,1.470] | 1.037 [0.739,1.454] | 1.020 [0.718,1.449] |
| Mixed | 0.985 [0.529,1.833] | 0.953 [0.505,1.799] | 0.958 [0.501,1.831] | 0.891 [0.471,1.683] | 0.959 [0.505,1.821] | 0.943 [0.507,1.753] | 0.896 [0.479,1.675] |
| South Asian | 1.490^***^ [1.189,1.868] | 1.384^*^ [1.011,1.894] | 1.387^*^ [1.011,1.903] | 1.270 [0.917,1.759] | 1.382^*^ [1.009,1.892] | 1.355 [0.974,1.885] | 1.279 [0.908,1.802] |
| Black | 1.489^***^ [1.215,1.825] | 1.405^*^ [1.075,1.836] | 1.416^*^ [1.085,1.847] | 1.324^*^ [1.011,1.734] | 1.388^*^ [1.062,1.813] | 1.355^*^ [1.031,1.781] | 1.289 [0.978,1.699] |
| Chinese | 2.683^***^ [1.635,4.402] | 2.479^**^ [1.436,4.281] | 2.564^***^ [1.479,4.445] | 2.460^**^ [1.398,4.330] | 2.402^**^ [1.357,4.251] | 2.377^**^ [1.367,4.131] | 2.373^**^ [1.326,4.246] |
| Other | 1.274 [0.895,1.812] | 1.189 [0.793,1.782] | 1.192 [0.792,1.795] | 1.116 [0.737,1.689] | 1.180 [0.786,1.772] | 1.165 [0.776,1.749] | 1.119 [0.735,1.702] |
| **Country of birth:** UK & Ireland |  | 1.000 - | 1.000 - | 1.000 - | 1.000 - | 1.000 - | 1.000 - |
| Elsewhere |  | 1.088 [0.850,1.392] | 1.068 [0.833,1.369] | 1.091 [0.848,1.404] | 1.092 [0.853,1.398] | 1.081 [0.843,1.386] | 1.094 [0.848,1.410] |
| **Healthcare worker status**: Non-healthcare worker |  |  | 1.000 - | 1.000 - |  |  | 1.000 - |
| Healthcare worker |  |  | 1.207^*^ [1.037,1.405] | 1.187^*^ [1.015,1.388] |  |  | 1.185^*^ [1.013,1.387] |
| Not in employment |  |  | 0.973 [0.852,1.112] | - |  |  | - |
| **Household size:** 1 |  |  |  | 1.000 - |  |  | 1.000 - |
| 2 |  |  |  | 1.111 [0.961,1.285] |  |  | 1.106 [0.957,1.279] |
| 3 |  |  |  | 1.062 [0.891,1.264] |  |  | 1.036 [0.869,1.234] |
| 4+ |  |  |  | 1.177 [0.983,1.409] |  |  | 1.172 [0.976,1.406] |
| **Education level:** College or University degree |  |  |  | 1.000 - |  |  | 1.000 - |
| A levels/AS levels |  |  |  | 1.060 [0.877,1.282] |  |  | 1.043 [0.862,1.262] |
| O levels/GCSEs/CSEs |  |  |  | 1.131 [0.976,1.311] |  |  | 1.092 [0.942,1.266] |
| Other |  |  |  | 1.236^*^ [1.045,1.461] |  |  | 1.207^*^ [1.019,1.428] |
| None of the above |  |  |  | 1.210^*^ [1.032,1.419] |  |  | 1.180^*^ [1.005,1.385] |
| **Socioeconomic deprivation:** Quartile 1 (most advantaged) |  |  |  | 1.000 - |  |  | 1.000 - |
| Quartile 2 |  |  |  | 1.023 [0.865,1.210] |  |  | 1.020 [0.862,1.207] |
| Quartile 3 |  |  |  | 1.012 [0.861,1.191] |  |  | 1.011 [0.860,1.190] |
| Quartile 4 (least advantaged) |  |  |  | 1.135 [0.962,1.340] |  |  | 1.114 [0.943,1.316] |
| **Housing tenure:** Own |  |  |  | 1.000 - |  |  | 1.000 - |
| Rent/Other |  |  |  | 1.030 [0.892,1.191] |  |  | 1.023 [0.882,1.186] |
| **Urban/rural:** Urban |  |  |  | 1.000 - |  |  | 1.000 - |
| Rural |  |  |  | 0.854 [0.700,1.040] |  |  | 0.868 [0.712,1.059] |
| **Employment status:** In paid employment or self-employed |  |  |  | 1.000 - |  |  | 1.000 - |
| Retired |  |  |  | 1.055 [0.891,1.250] |  |  | 1.038 [0.875,1.231] |
| Looking after home and/or family |  |  |  | 1.313 [0.929,1.855] |  |  | 1.309 [0.928,1.846] |
| Unable to work because of sickness or disability |  |  |  | 0.914 [0.718,1.163] |  |  | 0.919 [0.710,1.190] |
| Unemployed |  |  |  | 0.711 [0.461,1.098] |  |  | 0.723 [0.467,1.120] |
| Other |  |  |  | 0.867 [0.541,1.391] |  |  | 0.842 [0.523,1.355] |
| **Manual occupation:**  Non-manual |  |  |  | 1.000 - |  |  | 1.000 - |
| Manual |  |  |  | 1.221^*^ [1.046,1.424] |  |  | 1.206^*^ [1.033,1.408] |
| Not in employment |  |  |  | - |  |  | - |
| **Long-standing illness/disability:** No |  |  |  |  | 1.000 - |  | 1.000 - |
| Yes |  |  |  |  | 1.026 [0.904,1.164] |  | 1.046 [0.920,1.190] |
| **Number of chronic conditions:** 0 |  |  |  |  | 1.000 - |  | 1.000 - |
| 1 |  |  |  |  | 0.997 [0.868,1.145] |  | 0.998 [0.869,1.146] |
| 2 |  |  |  |  | 1.080 [0.921,1.267] |  | 1.059 [0.902,1.245] |
| 3 |  |  |  |  | 1.121 [0.919,1.366] |  | 1.093 [0.894,1.336] |
| 4+ |  |  |  |  | 0.984 [0.774,1.251] |  | 0.946 [0.739,1.211] |
| **Self-reported health:** Excellent |  |  |  |  | 1.000 - |  | 1.000 - |
| Good |  |  |  |  | 1.021 [0.863,1.207] |  | 0.990 [0.838,1.170] |
| Fair |  |  |  |  | 1.042 [0.861,1.261] |  | 0.972 [0.801,1.180] |
| Poor |  |  |  |  | 0.972 [0.757,1.249] |  | 0.921 [0.712,1.191] |
| **Body Mass Index:** Underweight (<18.5) |  |  |  |  |  | 1.191 [0.663,2.139] | 1.154 [0.645,2.064] |
| Normal weight (18.5-24.9) |  |  |  |  |  | 1.000 - | 1.000 - |
| Overweight (25.0-29.9) |  |  |  |  |  | 1.140 [0.995,1.307] | 1.114 [0.970,1.279] |
| Obese (>=30.0) |  |  |  |  |  | 1.235^**^ [1.072,1.423] | 1.203^*^ [1.038,1.394] |
| **Smoking status:** Never |  |  |  |  |  | 1.000 - | 1.000 - |
| Previous |  |  |  |  |  | 1.132^*^ [1.011,1.266] | 1.105 [0.988,1.237] |
| Current |  |  |  |  |  | 0.945 [0.795,1.123] | 0.942 [0.788,1.126] |
| **Alcohol consumption:** Daily or almost daily |  |  |  |  |  | 1.000 - | 1.000 - |
| Three or four times a week |  |  |  |  |  | 1.039 [0.871,1.239] | 1.014 [0.851,1.209] |
| Once or twice a week |  |  |  |  |  | 1.187^*^ [1.009,1.397] | 1.164 [0.987,1.373] |
| One to three times a month |  |  |  |  |  | 1.225^*^ [1.011,1.485] | 1.185 [0.977,1.437] |
| Special occasions only |  |  |  |  |  | 1.088 [0.898,1.320] | 1.054 [0.868,1.281] |
| Never (former drinker) |  |  |  |  |  | 1.232 [0.979,1.549] | 1.177 [0.925,1.498] |
| Never |  |  |  |  |  | 1.172 [0.920,1.495] | 1.094 [0.854,1.403] |
| **Observations** | 2658 | 2658 | 2658 | 2658 | 2658 | 2658 | 2658 |

Table S17: Socioeconomic deprivation and risk of testing positive amongst those tested

|  | **Model 1** | **Model 2** | **Model 3** | **Model 4** | **Model 5** | **Model 6** |
| --- | --- | --- | --- | --- | --- | --- |
| **Tested positive for SARS-CoV-2** | **RR**  **[95% CI]** | **RR**  **[95% CI]** | **RR**  **[95% CI]** | **RR**  **[95% CI]** | **RR**  **[95% CI]** | **RR**  **[95% CI]** |
| **Age group:** 40-44 | 1.000 - | 1.000 - | 1.000 - | 1.000 - | 1.000 - | 1.000 - |
| 45-49 | 0.973 [0.809,1.172] | 0.970 [0.806,1.167] | 0.967 [0.802,1.166] | 0.964 [0.801,1.162] | 0.974 [0.809,1.172] | 0.972 [0.806,1.174] |
| 50-54 | 0.882 [0.724,1.073] | 0.885 [0.727,1.079] | 0.907 [0.742,1.109] | 0.878 [0.719,1.072] | 0.892 [0.733,1.085] | 0.906 [0.741,1.109] |
| 55-59 | 0.890 [0.732,1.081] | 0.900 [0.741,1.094] | 0.956 [0.777,1.176] | 0.881 [0.722,1.076] | 0.890 [0.732,1.081] | 0.933 [0.755,1.151] |
| 60-64 | 0.799^*^ [0.670,0.954] | 0.821^*^ [0.687,0.982] | 0.851 [0.684,1.058] | 0.803^*^ [0.668,0.965] | 0.810^*^ [0.676,0.970] | 0.833 [0.666,1.040] |
| 65-69 | 0.935 [0.793,1.103] | 0.967 [0.818,1.142] | 0.986 [0.779,1.246] | 0.936 [0.786,1.114] | 0.946 [0.799,1.121] | 0.958 [0.754,1.219] |
| 70+ | 1.119 [0.707,1.770] | 1.142 [0.725,1.799] | 1.143 [0.697,1.872] | 1.085 [0.684,1.721] | 1.110 [0.698,1.764] | 1.084 [0.652,1.803] |
| **Sex:** Female | 1.000 - | 1.000 - | 1.000 - | 1.000 - | 1.000 - | 1.000 - |
| Male | 1.220^***^ [1.101,1.351] | 1.210^***^ [1.093,1.340] | 1.246^***^ [1.118,1.387] | 1.215^***^ [1.096,1.346] | 1.204^***^ [1.083,1.338] | 1.240^***^ [1.109,1.385] |
| **Assessment centre**: Manchester | 0.925 [0.697,1.227] | 0.905 [0.683,1.199] | 0.908 [0.687,1.200] | 0.908 [0.685,1.203] | 0.921 [0.696,1.220] | 0.921 [0.698,1.217] |
| Oxford | 0.669^*^ [0.452,0.992] | 0.641^*^ [0.432,0.953] | 0.665^*^ [0.449,0.985] | 0.642^*^ [0.432,0.954] | 0.680 [0.457,1.012] | 0.694 [0.468,1.030] |
| Stoke | 1.009 [0.762,1.336] | 0.995 [0.751,1.319] | 0.977 [0.741,1.287] | 1.002 [0.756,1.328] | 0.988 [0.747,1.306] | 0.980 [0.744,1.290] |
| Reading | 0.901 [0.679,1.196] | 0.860 [0.648,1.141] | 0.863 [0.651,1.145] | 0.861 [0.649,1.144] | 0.885 [0.668,1.173] | 0.884 [0.666,1.173] |
| Bury | 0.867 [0.667,1.128] | 0.842 [0.647,1.096] | 0.839 [0.646,1.090] | 0.841 [0.647,1.095] | 0.842 [0.648,1.095] | 0.837 [0.644,1.086] |
| Newcastle | 1.000 - | 1.000 - | 1.000 - | 1.000 - | 1.000 - | 1.000 - |
| Leeds | 1.755^***^ [1.443,2.135] | 1.669^***^ [1.369,2.036] | 1.703^***^ [1.396,2.078] | 1.680^***^ [1.376,2.050] | 1.673^***^ [1.369,2.044] | 1.713^***^ [1.401,2.096] |
| Bristol | 0.632^**^ [0.473,0.844] | 0.613^***^ [0.458,0.819] | 0.610^***^ [0.457,0.814] | 0.612^***^ [0.458,0.818] | 0.612^***^ [0.458,0.819] | 0.609^***^ [0.456,0.814] |
| Barts | 0.682^*^ [0.486,0.956] | 0.621^**^ [0.443,0.869] | 0.647^*^ [0.462,0.908] | 0.622^**^ [0.444,0.873] | 0.670^*^ [0.477,0.940] | 0.685^*^ [0.488,0.964] |
| Nottingham | 0.757 [0.573,1.001] | 0.739^*^ [0.559,0.977] | 0.756^*^ [0.572,0.998] | 0.736^*^ [0.556,0.976] | 0.745^*^ [0.562,0.987] | 0.754 [0.568,1.001] |
| Sheffield | 0.749^*^ [0.589,0.952] | 0.730^*^ [0.574,0.928] | 0.735^*^ [0.578,0.934] | 0.728^**^ [0.573,0.926] | 0.738^*^ [0.582,0.937] | 0.737^*^ [0.581,0.935] |
| Liverpool | 0.949 [0.760,1.184] | 0.934 [0.748,1.165] | 0.928 [0.745,1.157] | 0.934 [0.748,1.166] | 0.934 [0.748,1.166] | 0.927 [0.743,1.157] |
| Middlesborough | 0.730^*^ [0.545,0.977] | 0.717^*^ [0.535,0.959] | 0.730^*^ [0.547,0.974] | 0.716^*^ [0.535,0.959] | 0.715^*^ [0.534,0.956] | 0.725^*^ [0.543,0.968] |
| Hounslow | 1.034 [0.820,1.304] | 0.932 [0.732,1.186] | 0.959 [0.753,1.222] | 0.938 [0.737,1.193] | 0.958 [0.754,1.218] | 0.985 [0.773,1.254] |
| Croydon | 1.107 [0.889,1.379] | 0.972 [0.770,1.228] | 1.024 [0.809,1.297] | 0.976 [0.773,1.233] | 0.997 [0.789,1.261] | 1.043 [0.823,1.322] |
| Birmingham | 0.986 [0.774,1.258] | 0.930 [0.727,1.190] | 0.920 [0.719,1.178] | 0.923 [0.721,1.183] | 0.941 [0.735,1.204] | 0.927 [0.722,1.189] |
| **Socioeconomic deprivation:** Quartile 1 (most advantaged) | 1.000 - | 1.000 - | 1.000 - | 1.000 - | 1.000 - | 1.000 - |
| Quartile 2 | 1.035 [0.874,1.224] | 1.039 [0.878,1.229] | 1.023 [0.865,1.210] | 1.039 [0.878,1.229] | 1.035 [0.875,1.225] | 1.020 [0.862,1.207] |
| Quartile 3 | 1.050 [0.894,1.233] | 1.039 [0.884,1.220] | 1.012 [0.861,1.191] | 1.041 [0.885,1.223] | 1.028 [0.875,1.207] | 1.011 [0.860,1.190] |
| Quartile 4 (least advantaged) | 1.209^*^ [1.038,1.408] | 1.164 [0.997,1.358] | 1.135 [0.962,1.340] | 1.158 [0.989,1.355] | 1.133 [0.968,1.326] | 1.114 [0.943,1.316] |
| **Ethnicity:** White British |  | 1.000 - | 1.000 - | 1.000 - | 1.000 - | 1.000 - |
| White Irish |  | 1.158 [0.871,1.539] | 1.133 [0.853,1.505] | 1.164 [0.874,1.549] | 1.147 [0.864,1.524] | 1.132 [0.851,1.507] |
| White Other |  | 1.030 [0.734,1.444] | 1.020 [0.718,1.448] | 1.038 [0.739,1.459] | 1.034 [0.737,1.449] | 1.020 [0.718,1.449] |
| Mixed |  | 0.925 [0.492,1.739] | 0.891 [0.471,1.683] | 0.932 [0.493,1.760] | 0.919 [0.495,1.704] | 0.896 [0.479,1.675] |
| South Asian |  | 1.341 [0.981,1.834] | 1.270 [0.917,1.759] | 1.344 [0.983,1.839] | 1.327 [0.954,1.845] | 1.279 [0.908,1.802] |
| Black |  | 1.351^*^ [1.033,1.765] | 1.324^*^ [1.011,1.734] | 1.338^*^ [1.023,1.748] | 1.316^*^ [1.001,1.731] | 1.289 [0.978,1.699] |
| Chinese |  | 2.408^**^ [1.339,4.329] | 2.460^**^ [1.398,4.330] | 2.339^**^ [1.271,4.305] | 2.325^**^ [1.295,4.173] | 2.373^**^ [1.326,4.246] |
| Other |  | 1.156 [0.770,1.735] | 1.116 [0.737,1.689] | 1.148 [0.763,1.727] | 1.141 [0.758,1.717] | 1.119 [0.735,1.702] |
| **Country of birth:** UK & Ireland |  | 1.000 - | 1.000 - | 1.000 - | 1.000 - | 1.000 - |
| Elsewhere |  | 1.088 [0.852,1.389] | 1.091 [0.848,1.404] | 1.092 [0.855,1.395] | 1.082 [0.846,1.385] | 1.094 [0.848,1.410] |
| **Household size:** 1 |  |  | 1.000 - |  |  | 1.000 - |
| 2 |  |  | 1.111 [0.961,1.285] |  |  | 1.106 [0.957,1.279] |
| 3 |  |  | 1.062 [0.891,1.264] |  |  | 1.036 [0.869,1.234] |
| 4+ |  |  | 1.177 [0.983,1.409] |  |  | 1.172 [0.976,1.406] |
| **Education level:** College or University degree |  |  | 1.000 - |  |  | 1.000 - |
| A levels/AS levels |  |  | 1.060 [0.877,1.282] |  |  | 1.043 [0.862,1.262] |
| O levels/GCSEs/CSEs |  |  | 1.131 [0.976,1.311] |  |  | 1.092 [0.942,1.266] |
| Other |  |  | 1.236^*^ [1.045,1.461] |  |  | 1.207^*^ [1.019,1.428] |
| None of the above |  |  | 1.210^*^ [1.032,1.419] |  |  | 1.180^*^ [1.005,1.385] |
| **Housing tenure:** Own |  |  | 1.000 - |  |  | 1.000 - |
| Rent/Other |  |  | 1.030 [0.892,1.191] |  |  | 1.023 [0.882,1.186] |
| **Urban/rural:** Urban |  |  | 1.000 - |  |  | 1.000 - |
| Rural |  |  | 0.854 [0.700,1.040] |  |  | 0.868 [0.712,1.059] |
| **Employment status:** In paid employment or self-employed |  |  | 1.000 - |  |  | 1.000 - |
| Retired |  |  | 1.055 [0.891,1.250] |  |  | 1.038 [0.875,1.231] |
| Looking after home and/or family |  |  | 1.313 [0.929,1.855] |  |  | 1.309 [0.928,1.846] |
| Unable to work because of sickness or disability |  |  | 0.914 [0.718,1.163] |  |  | 0.919 [0.710,1.190] |
| Unemployed |  |  | 0.711 [0.461,1.098] |  |  | 0.723 [0.467,1.120] |
| Other |  |  | 0.867 [0.541,1.391] |  |  | 0.842 [0.523,1.355] |
| **Manual occupation:** Non-manual |  |  | 1.000 - |  |  | 1.000 - |
| Manual |  |  | 1.221^*^ [1.046,1.424] |  |  | 1.206^*^ [1.033,1.408] |
| Not in employment |  |  | - |  |  | - |
| **Healthcare worker status**: Non-healthcare worker |  |  | 1.000 - |  |  | 1.000 - |
| Healthcare worker |  |  | 1.187^*^ [1.015,1.388] |  |  | 1.185^*^ [1.013,1.387] |
| Not in employment |  |  | - |  |  | - |
| **Long-standing illness/disability:** No |  |  |  | 1.000 - |  | 1.000 - |
| Yes |  |  |  | 1.019 [0.897,1.157] |  | 1.046 [0.920,1.190] |
| **Number of chronic conditions:** 0 |  |  |  | 1.000 - |  | 1.000 - |
| 1 |  |  |  | 1.002 [0.872,1.150] |  | 0.998 [0.869,1.146] |
| 2 |  |  |  | 1.074 [0.916,1.260] |  | 1.059 [0.902,1.245] |
| 3 |  |  |  | 1.113 [0.913,1.358] |  | 1.093 [0.894,1.336] |
| 4+ |  |  |  | 0.970 [0.762,1.234] |  | 0.946 [0.739,1.211] |
| **Self-reported health:** Excellent |  |  |  | 1.000 - |  | 1.000 - |
| Good |  |  |  | 1.019 [0.862,1.204] |  | 0.990 [0.838,1.170] |
| Fair |  |  |  | 1.028 [0.849,1.244] |  | 0.972 [0.801,1.180] |
| Poor |  |  |  | 0.954 [0.743,1.226] |  | 0.921 [0.712,1.191] |
| **Body Mass Index:** Underweight (<18.5) |  |  |  |  | 1.186 [0.657,2.142] | 1.154 [0.645,2.064] |
| Normal weight (18.5-24.9) |  |  |  |  | 1.000 - | 1.000 - |
| Overweight (25.0-29.9) |  |  |  |  | 1.138 [0.993,1.304] | 1.114 [0.970,1.279] |
| Obese (>=30.0) |  |  |  |  | 1.218^**^ [1.056,1.405] | 1.203^*^ [1.038,1.394] |
| **Smoking status:** Never |  |  |  |  | 1.000 - | 1.000 - |
| Previous |  |  |  |  | 1.126^*^ [1.006,1.260] | 1.105 [0.988,1.237] |
| Current |  |  |  |  | 0.928 [0.779,1.105] | 0.942 [0.788,1.126] |
| **Alcohol consumption:** Daily or almost daily |  |  |  |  | 1.000 - | 1.000 - |
| Three or four times a week |  |  |  |  | 1.036 [0.869,1.235] | 1.014 [0.851,1.209] |
| Once or twice a week |  |  |  |  | 1.183^*^ [1.006,1.393] | 1.164 [0.987,1.373] |
| One to three times a month |  |  |  |  | 1.220^*^ [1.007,1.478] | 1.185 [0.977,1.437] |
| Special occasions only |  |  |  |  | 1.080 [0.891,1.310] | 1.054 [0.868,1.281] |
| Never (former drinker) |  |  |  |  | 1.200 [0.951,1.514] | 1.177 [0.925,1.498] |
| Never |  |  |  |  | 1.158 [0.908,1.476] | 1.094 [0.854,1.403] |
| **Observations** | 2658 | 2658 | 2658 | 2658 | 2658 | 2658 |

Table S18: Education level and risk of testing positive amongst those tested

|  | **Model 1** | **Model 2** | **Model 3** | **Model 4** | **Model 5** | **Model 6** |
| --- | --- | --- | --- | --- | --- | --- |
| **Tested positive for SARS-CoV-2** | **RR**  **[95% CI]** | **RR**  **[95% CI]** | **RR**  **[95% CI]** | **RR**  **[95% CI]** | **RR**  **[95% CI]** | **RR**  **[95% CI]** |
| **Age group:** 40-44 | 1.000 - | 1.000 - | 1.000 - | 1.000 - | 1.000 - | 1.000 - |
| 45-49 | 0.951 [0.789,1.148] | 0.949 [0.787,1.144] | 0.967 [0.802,1.166] | 0.944 [0.783,1.139] | 0.957 [0.794,1.154] | 0.972 [0.806,1.174] |
| 50-54 | 0.859 [0.706,1.045] | 0.863 [0.709,1.051] | 0.907 [0.742,1.109] | 0.856 [0.702,1.045] | 0.871 [0.716,1.060] | 0.906 [0.741,1.109] |
| 55-59 | 0.852 [0.700,1.037] | 0.864 [0.710,1.052] | 0.956 [0.777,1.176] | 0.845 [0.691,1.032] | 0.858 [0.704,1.045] | 0.933 [0.755,1.151] |
| 60-64 | 0.742^**^ [0.618,0.890] | 0.767^**^ [0.638,0.922] | 0.851 [0.684,1.058] | 0.748^**^ [0.620,0.903] | 0.762^**^ [0.632,0.918] | 0.833 [0.666,1.040] |
| 65-69 | 0.863 [0.726,1.027] | 0.898 [0.753,1.070] | 0.986 [0.779,1.246] | 0.868 [0.724,1.040] | 0.888 [0.743,1.061] | 0.958 [0.754,1.219] |
| 70+ | 1.014 [0.638,1.611] | 1.048 [0.663,1.657] | 1.143 [0.697,1.872] | 0.993 [0.624,1.581] | 1.029 [0.645,1.641] | 1.084 [0.652,1.803] |
| **Sex:** Female | 1.000 - | 1.000 - | 1.000 - | 1.000 - | 1.000 - | 1.000 - |
| Male | 1.219^***^ [1.100,1.350] | 1.209^***^ [1.092,1.339] | 1.246^***^ [1.118,1.387] | 1.214^***^ [1.095,1.345] | 1.200^***^ [1.079,1.334] | 1.240^***^ [1.109,1.385] |
| **Assessment centre:** Manchester | 0.946 [0.714,1.252] | 0.922 [0.697,1.219] | 0.908 [0.687,1.200] | 0.922 [0.698,1.219] | 0.935 [0.708,1.235] | 0.921 [0.698,1.217] |
| Oxford | 0.667^*^ [0.452,0.983] | 0.643^*^ [0.435,0.952] | 0.665^*^ [0.449,0.985] | 0.643^*^ [0.434,0.952] | 0.678 [0.458,1.005] | 0.694 [0.468,1.030] |
| Stoke | 0.969 [0.734,1.279] | 0.966 [0.732,1.274] | 0.977 [0.741,1.287] | 0.975 [0.738,1.287] | 0.964 [0.731,1.270] | 0.980 [0.744,1.290] |
| Reading | 0.873 [0.660,1.155] | 0.840 [0.636,1.110] | 0.863 [0.651,1.145] | 0.842 [0.637,1.114] | 0.868 [0.657,1.147] | 0.884 [0.666,1.173] |
| Bury | 0.847 [0.652,1.101] | 0.826 [0.635,1.074] | 0.839 [0.646,1.090] | 0.824 [0.634,1.071] | 0.828 [0.637,1.075] | 0.837 [0.644,1.086] |
| Newcastle | 1.000 - | 1.000 - | 1.000 - | 1.000 - | 1.000 - | 1.000 - |
| Leeds | 1.745^***^ [1.432,2.126] | 1.663^***^ [1.362,2.030] | 1.703^***^ [1.396,2.078] | 1.675^***^ [1.371,2.046] | 1.665^***^ [1.361,2.037] | 1.713^***^ [1.401,2.096] |
| Bristol | 0.617^**^ [0.462,0.825] | 0.603^***^ [0.451,0.805] | 0.610^***^ [0.457,0.814] | 0.602^***^ [0.450,0.804] | 0.604^***^ [0.452,0.808] | 0.609^***^ [0.456,0.814] |
| Barts | 0.779 [0.557,1.087] | 0.685^*^ [0.490,0.957] | 0.647^*^ [0.462,0.908] | 0.683^*^ [0.488,0.955] | 0.723 [0.517,1.011] | 0.685^*^ [0.488,0.964] |
| Nottingham | 0.739^*^ [0.560,0.976] | 0.725^*^ [0.549,0.957] | 0.756^*^ [0.572,0.998] | 0.721^*^ [0.545,0.953] | 0.732^*^ [0.553,0.968] | 0.754 [0.568,1.001] |
| Sheffield | 0.747^*^ [0.587,0.951] | 0.730^*^ [0.574,0.928] | 0.735^*^ [0.578,0.934] | 0.729^**^ [0.573,0.926] | 0.737^*^ [0.581,0.935] | 0.737^*^ [0.581,0.935] |
| Liverpool | 0.942 [0.755,1.175] | 0.928 [0.744,1.157] | 0.928 [0.745,1.157] | 0.928 [0.743,1.158] | 0.928 [0.744,1.159] | 0.927 [0.743,1.157] |
| Middlesborough | 0.725^*^ [0.541,0.971] | 0.715^*^ [0.534,0.957] | 0.730^*^ [0.547,0.974] | 0.714^*^ [0.533,0.957] | 0.714^*^ [0.533,0.955] | 0.725^*^ [0.543,0.968] |
| Hounslow | 1.084 [0.860,1.368] | 0.965 [0.758,1.228] | 0.959 [0.753,1.222] | 0.970 [0.762,1.235] | 0.983 [0.773,1.251] | 0.985 [0.773,1.254] |
| Croydon | 1.165 [0.934,1.454] | 1.010 [0.799,1.276] | 1.024 [0.809,1.297] | 1.012 [0.801,1.279] | 1.027 [0.812,1.299] | 1.043 [0.823,1.322] |
| Birmingham | 0.994 [0.780,1.266] | 0.931 [0.729,1.191] | 0.920 [0.719,1.178] | 0.924 [0.721,1.183] | 0.941 [0.736,1.204] | 0.927 [0.722,1.189] |
| **Education level:** College or University degree | 1.000 - | 1.000 - | 1.000 - | 1.000 - | 1.000 - | 1.000 - |
| A levels/AS levels | 1.049 [0.867,1.270] | 1.057 [0.873,1.279] | 1.060 [0.877,1.282] | 1.048 [0.866,1.269] | 1.045 [0.862,1.265] | 1.043 [0.862,1.262] |
| O levels/GCSEs/CSEs | 1.121 [0.971,1.295] | 1.135 [0.982,1.311] | 1.131 [0.976,1.311] | 1.132 [0.979,1.309] | 1.093 [0.945,1.263] | 1.092 [0.942,1.266] |
| Other | 1.310^**^ [1.111,1.544] | 1.301^**^ [1.104,1.533] | 1.236^*^ [1.045,1.461] | 1.306^**^ [1.107,1.541] | 1.257^**^ [1.066,1.482] | 1.207^*^ [1.019,1.428] |
| None of the above | 1.227^**^ [1.055,1.428] | 1.230^**^ [1.057,1.430] | 1.210^*^ [1.032,1.419] | 1.228^**^ [1.053,1.432] | 1.188^*^ [1.018,1.386] | 1.180^*^ [1.005,1.385] |
| **Ethnicity:** White British |  | 1.000 - | 1.000 - | 1.000 - | 1.000 - | 1.000 - |
| White Irish |  | 1.142 [0.861,1.515] | 1.133 [0.853,1.505] | 1.150 [0.865,1.529] | 1.131 [0.852,1.500] | 1.132 [0.851,1.507] |
| White Other |  | 1.071 [0.758,1.512] | 1.020 [0.718,1.448] | 1.079 [0.762,1.527] | 1.067 [0.756,1.504] | 1.020 [0.718,1.449] |
| Mixed |  | 0.969 [0.517,1.815] | 0.891 [0.471,1.683] | 0.972 [0.517,1.829] | 0.955 [0.516,1.767] | 0.896 [0.479,1.675] |
| South Asian |  | 1.358 [0.987,1.868] | 1.270 [0.917,1.759] | 1.360 [0.989,1.871] | 1.347 [0.963,1.885] | 1.279 [0.908,1.802] |
| Black |  | 1.377^*^ [1.047,1.810] | 1.324^*^ [1.011,1.734] | 1.362^*^ [1.036,1.791] | 1.339^*^ [1.013,1.769] | 1.289 [0.978,1.699] |
| Chinese |  | 2.560^**^ [1.416,4.630] | 2.460^**^ [1.398,4.330] | 2.483^**^ [1.343,4.592] | 2.475^**^ [1.374,4.459] | 2.373^**^ [1.326,4.246] |
| Other |  | 1.180 [0.786,1.772] | 1.116 [0.737,1.689] | 1.174 [0.780,1.765] | 1.169 [0.777,1.759] | 1.119 [0.735,1.702] |
| **Country of birth:** UK & Ireland |  | 1.000 - | 1.000 - | 1.000 - | 1.000 - | 1.000 - |
| Elsewhere |  | 1.101 [0.854,1.419] | 1.091 [0.848,1.404] | 1.106 [0.858,1.426] | 1.093 [0.847,1.410] | 1.094 [0.848,1.410] |
| **Household size:** 1 |  |  | 1.000 - |  |  | 1.000 - |
| 2 |  |  | 1.111 [0.961,1.285] |  |  | 1.106 [0.957,1.279] |
| 3 |  |  | 1.062 [0.891,1.264] |  |  | 1.036 [0.869,1.234] |
| 4+ |  |  | 1.177 [0.983,1.409] |  |  | 1.172 [0.976,1.406] |
| **Socioeconomic deprivation:** Quartile 1 (most advantaged) |  |  | 1.000 - |  |  | 1.000 - |
| Quartile 2 |  |  | 1.023 [0.865,1.210] |  |  | 1.020 [0.862,1.207] |
| Quartile 3 |  |  | 1.012 [0.861,1.191] |  |  | 1.011 [0.860,1.190] |
| Quartile 4 (least advantaged) |  |  | 1.135 [0.962,1.340] |  |  | 1.114 [0.943,1.316] |
| **Housing tenure:** Own |  |  | 1.000 - |  |  | 1.000 - |
| Rent/Other |  |  | 1.030 [0.892,1.191] |  |  | 1.023 [0.882,1.186] |
| **Urban/rural:** Urban |  |  | 1.000 - |  |  | 1.000 - |
| Rural |  |  | 0.854 [0.700,1.040] |  |  | 0.868 [0.712,1.059] |
| **Employment status:** In paid employment or self-employed |  |  | 1.000 - |  |  | 1.000 - |
| Retired |  |  | 1.055 [0.891,1.250] |  |  | 1.038 [0.875,1.231] |
| Looking after home and/or family |  |  | 1.313 [0.929,1.855] |  |  | 1.309 [0.928,1.846] |
| Unable to work because of sickness or disability |  |  | 0.914 [0.718,1.163] |  |  | 0.919 [0.710,1.190] |
| Unemployed |  |  | 0.711 [0.461,1.098] |  |  | 0.723 [0.467,1.120] |
| Other |  |  | 0.867 [0.541,1.391] |  |  | 0.842 [0.523,1.355] |
| **Manual occupation:** Non-manual |  |  | 1.000 - |  |  | 1.000 - |
| Manual |  |  | 1.221^*^ [1.046,1.424] |  |  | 1.206^*^ [1.033,1.408] |
| Not in employment |  |  | - |  |  | - |
| **Healthcare worker status:** Non-healthcare worker |  |  | 1.000 - |  |  | 1.000 - |
| Healthcare worker |  |  | 1.187^*^ [1.015,1.388] |  |  | 1.185^*^ [1.013,1.387] |
| Not in employment |  |  | - |  |  | - |
| **Long-standing illness/disability:** No |  |  |  | 1.000 - |  | 1.000 - |
| Yes |  |  |  | 1.038 [0.915,1.178] |  | 1.046 [0.920,1.190] |
| **Number of chronic conditions:** 0 |  |  |  | 1.000 - |  | 1.000 - |
| 1 |  |  |  | 0.989 [0.860,1.136] |  | 0.998 [0.869,1.146] |
| 2 |  |  |  | 1.066 [0.908,1.250] |  | 1.059 [0.902,1.245] |
| 3 |  |  |  | 1.111 [0.910,1.356] |  | 1.093 [0.894,1.336] |
| 4+ |  |  |  | 0.954 [0.749,1.214] |  | 0.946 [0.739,1.211] |
| **Self-reported health:** Excellent |  |  |  | 1.000 - |  | 1.000 - |
| Good |  |  |  | 1.004 [0.850,1.187] |  | 0.990 [0.838,1.170] |
| Fair |  |  |  | 1.006 [0.831,1.218] |  | 0.972 [0.801,1.180] |
| Poor |  |  |  | 0.929 [0.723,1.193] |  | 0.921 [0.712,1.191] |
| **Body Mass Index:** Underweight (<18.5) |  |  |  |  | 1.200 [0.654,2.203] | 1.154 [0.645,2.064] |
| Normal weight (18.5-24.9) |  |  |  |  | 1.000 - | 1.000 - |
| Overweight (25.0-29.9) |  |  |  |  | 1.131 [0.987,1.296] | 1.114 [0.970,1.279] |
| Obese (>=30.0) |  |  |  |  | 1.213^**^ [1.052,1.398] | 1.203^*^ [1.038,1.394] |
| **Smoking status:** Never |  |  |  |  | 1.000 - | 1.000 - |
| Previous |  |  |  |  | 1.123^*^ [1.003,1.257] | 1.105 [0.988,1.237] |
| Current |  |  |  |  | 0.923 [0.776,1.098] | 0.942 [0.788,1.126] |
| **Alcohol consumption:** Daily or almost daily |  |  |  |  | 1.000 - | 1.000 - |
| Three or four times a week |  |  |  |  | 1.026 [0.861,1.223] | 1.014 [0.851,1.209] |
| Once or twice a week |  |  |  |  | 1.163 [0.987,1.371] | 1.164 [0.987,1.373] |
| One to three times a month |  |  |  |  | 1.192 [0.982,1.446] | 1.185 [0.977,1.437] |
| Special occasions only |  |  |  |  | 1.064 [0.877,1.292] | 1.054 [0.868,1.281] |
| Never (former drinker) |  |  |  |  | 1.192 [0.946,1.502] | 1.177 [0.925,1.498] |
| Never |  |  |  |  | 1.130 [0.884,1.445] | 1.094 [0.854,1.403] |
| **Observations** | 2658 | 2658 | 2658 | 2658 | 2658 | 2658 |
